# Genomic characterization and radiation tolerance of *Naganishia kalamii* sp. nov. and *Cystobasidium onofrii* sp. nov. from Mars 2020 mission assembly facilities

**DOI:** 10.1186/s43008-023-00119-4

**Published:** 2023-08-11

**Authors:** Patrick Leo, Marcus de Melo Texeira, Atul M. Chander, Nitin K. Singh, Anna C. Simpson, Andrey Yurkov, Fathi Karouia, Jason E. Stajich, Christopher E. Mason, Kasthuri Venkateswaran

**Affiliations:** 1 Department of Environmental Sciences, Informatics and Statistics, University Ca’ Foscari of Venice, Via Torino 155, 30172 Mestre, Italy; 2 Department of Ecological and Biological Sciences, University of Tuscia, Largo dell’università snc, 01100 Viterbo, Italy; 3 NASA-Jet Propulsion Laboratory, Biotechnology and Planetary Protection Group, California Institute of Technology, M/S 245-103, 4800 Oak Grove Dr., Pasadena, CA 91109 USA; 4 NASA-Jet Propulsion Laboratory, Biotechnology and Planetary Protection Group, California Institute of Technology, M/S 245-104, 4800 Oak Grove Dr., Pasadena, CA 91109 USA; 5 Pathogen and Microbiome Institute, Northern Arizona University, Flagstaff, AZ 86011 USA; 6 Núcleo de Medicina Tropical, Faculdade de Medicina, Universidade de Brasília, Brasília, 70910-900 Brazil; 7 Leibniz Institute DSMZ-German Collection of Microorganisms and Cell Cultures, Brunswick, Germany; 8 Blue Marble Space Institute of Science, Exobiology Branch, NASA Ames Research Center, PO BOX 1 MS 239/4, Moffett Field, CA 94035 USA; 9 Space Research Within Reach, San Francisco, CA 941110 USA; 10 Department of Microbiology and Plant Pathology, University of CA–Riverside, Riverside, CA 92521 USA; 11 Department of Physiology and Biophysics and the WorldQuant Initiative for Quantitative Prediction, Weill Cornell Medicine, New York, NY 10021 USA

**Keywords:** 2 new species, *Naganishia kalamii*, *Cystobasidium onofrii*, Mars 2020 mission, NASA cleanrooms, UV resistance, Extremophiles

## Abstract

During the construction and assembly of the Mars 2020 mission components at two different NASA cleanrooms, several fungal strains were isolated. Based on their colony morphology, two strains that showed yeast-like appearance were further characterized for their phylogenetic position. The species-level classification of these two novel strains, using traditional colony and cell morphology methods combined with the phylogenetic reconstructions using multi-locus sequence analysis (MLSA) based on several gene loci (ITS, LSU, SSU, *RPB*1, *RPB2*, *CYTB* and *TEF1*), and whole genome sequencing (WGS) was carried out. This polyphasic taxonomic approach supported the conclusion that the two basidiomycetous yeasts belong to hitherto undescribed species. The strain FJI-L2-BK-P3^T^, isolated from the Jet Propulsion Laboratory Spacecraft Assembly Facility, was placed in the *Naganishia albida* clade* (Filobasidiales, Tremellomycetes),* but is genetically and physiologically different from other members of the clade. Another yeast strain FKI-L6-BK-PAB1^T^, isolated from the Kennedy Space Center Payload Hazardous and Servicing Facility, was placed in the genus *Cystobasidium* (*Cystobasidiales, Cystobasidiomycetes*) and is distantly related to *C. benthicum.* Here we propose two novel species with the type strains, *Naganishia kalamii* sp. nov. (FJI-L2-BK-P3^T^ = NRRL 64466 = DSM 115730) and *Cystobasidium onofrii* sp. nov. (FKI-L6-BK-PAB1^T^ = NRRL 64426 = DSM 114625). The phylogenetic analyses revealed that single gene phylogenies (ITS or LSU) were not conclusive, and MLSA and WGS-based phylogenies were more advantageous for species discrimination in the two genera. The genomic analysis predicted proteins associated with dehydration and desiccation stress-response and the presence of genes that are directly related to osmotolerance and psychrotolerance in both novel yeasts described. Cells of these two newly-described yeasts were exposed to UV-C radiation and compared with *N. onofrii,* an extremophilic UV-C resistant cold-adapted Alpine yeast. Both novel species were UV resistant, emphasizing the need for collecting and characterizing extremotolerant microbes, including yeasts, to improve microbial reduction techniques used in NASA planetary protection programs.

**Supplementary Information:**

The online version contains supplementary material available at 10.1186/s43008-023-00119-4.

## INTRODUCTION

Many fungal species, including yeasts, have been isolated and characterized from different extreme environments, such as the Antarctic continent (rocks, soil brines), Arctic ice-sheet and sea-ice, deep sea, deserts, etc. (Buzzini and Margesin 2014; Segal-Kischinevzky et al. 2022; Coleine et al. 2022). Yeasts and yeast-like fungi isolated from Antarctic environments (proposed as resembling the surface and conditions of Mars and/or Icy Moons) were reported to successfully resist a wide spectrum of stressors which mimic conditions on other planetary bodies, including ionizing radiation, UV-flux irradiation, extremely low and high temperatures, desiccation, high salinity and acidic conditions (Onofri et al. 2004, 2020; Selbmann et al. 2005; Pacelli et al. 2017, 2021; Buzzini et al. 2018). Since National Aeronautics and Space Administration (NASA) Planetary Protection (PP) seeks to prevent anthropogenic microbial transport (forward contamination) to other planets (Cockell 2005; Conley and Rummel 2010), evaluation of hitchhiking extremophilic yeasts on spacecraft and associated components is necessary.

A wide range of microbial taxa, including both bacterial and fungal species, have been detected in various NASA spacecraft assembly cleanrooms (La Duc et al. 2012), but historically NASA and European Space Agency (ESA) PP policy has focused on monitoring and eradicating bacteria, specifically those producing endospores. However, the presence of fungi in these cleanrooms has also recently been assessed, since fungi are tolerant of a wide range of extreme conditions (Parker et al. 2022; Blachowicz et al. 2022; Chander et al. 2022b). Characterization of fungi (including yeast) is important both for assessing the risk of forward contamination and updating appropriate cleaning and sterilization strategies (Benardini and Moissl-Eichinger 2022). Therefore, newly discovered fungal (including yeasts) species in cleanrooms must be evaluated not only for their morphology, but also at the genomic level so that new countermeasures can be developed to eradicate them based on their biology and resilient properties (Chander et al. 2022b).

To date, only filamentous fungi have been described from cleanrooms (Parker et al. 2022; Blachowicz et al. 2022; Chander et al. 2022b). In contrast to filamentous fungi, yeasts are microscopic and predominantly unicellular (Boekhout et al. 2021). It has been shown that yeast and yeast-like fungi are capable of adapting to and colonizing various ecological niches due to their genetic and phenotypic plasticity and robustness (Ene et al. 2015; Ribeiro et al. 2022). Several important adaptations facilitate survival of yeast cells in harsh environments, including production of thick external polysaccharide capsules, forming biofilm, and UV protective compounds, melanin, carotenoids and mycosporines (Buzzini et al. 2018). In this study, we describe for the first time two novel yeast species from NASA cleanrooms, which are oligotrophic extreme environments.

To explore yeast diversity in various environments including NASA cleanrooms, understanding their phylogenetic diversity and ecology is necessary. Phylogenies based on 16S ribosomal RNA or average nucleotide identity (ANI) are most commonly used for prokaryote taxonomy, but fungal species are identified with nucleotide sequences of multiple conserved gene regions (Lücking et al. 2021). To identify yeasts, both phenotypic traits and molecular analyses are required (Boekhout et al. 2021). The modern yeast taxonomy includes genealogical approaches based on single locus (ITS or LSU), multi-locus sequence analysis (MLSA) and recently whole genome sequencing (WGS) (Libkind et al. 2020; Boekhout et al. 2021; Chander et al. 2022b). Molecular phylogeny for species recognition in Fungi is gaining broad attention (Lücking et al. 2021), but understanding species ecology requires investigating morphology, physiology, and biochemistry leading to the Consolidated Species Concept based on the so-called polyphasic approach (Boekhout et al. 2021).

One of the objectives of this study was to characterize two novel yeast species by employing the polyphasic taxonomic approach, which is necessary to formally describe the two novel taxa. Colony morphology, microscopic features, and physiological properties were combined with MLSA phylogeny (seven loci). In addition, a phylogenetic analysis based on available WGS was applied to test its utility for species discrimination. The assembled and characterized genomes of *Naganishia kalamii* FJI-L2-BK-P3^T^ and *Cystobasidium onofrii* FKI-L6-BK-PAB1^T^ were compared with genomes of other closely related species to identify unique and shared evolutionary characters. Considering unique properties of the habitat challenging the microbial life, the third objective of this study was to assess the UV-C resistance of the two novel yeast species and compared with a UV-C resistant extremophilic yeast, *Naganishia onofrii* strain DBVPG 5303 from Miage glacier (Mont Blanc, Alps, Italy) at the altitude of 3892 m above sea level (Turchetti et al. 2015).

## MATERIALS AND METHODS

### Sample collection and isolation of yeasts

Steps for sample collection and isolation of yeasts at the Jet Propulsion Laboratory Spacecraft Assembly Facility (JPL-SAF) and the Kennedy Space Center Payload Hazardous and Servicing Facility (KSC-PHSF) cleanrooms were previously described (Parker et al. 2022; Blachowicz et al. 2022). Briefly, samples were collected from cleanroom surfaces using moist polyester wipes and were agitated in 200 mL sterile phosphate-buffered saline (PBS) and shaken for 1 min. The resulting PBS suspension was concentrated using an Innova Prep LLC concentrating pipette and spread onto plates containing potato dextrose agar (PDA, Difco, #213,400) mixed with chloramphenicol (25 µg/mL). Plates were incubated at 25 °C for 7–14 days, and yeast strains were isolated, purified, and stored for further characterization.

Steps for determination of microscopic traits and observation and microscopic morphology were followed as per previous protocol (Chander et al. 2022b). In a nutshell, a small block of agar was placed in the center of a sterile slide, and all four sides of the agar were inoculated with the yeast suspension. Subsequently, a sterile cover slip was gently placed on top of the agar block. The slide culture was kept in a moist Petri dish lined with filter paper soaked in sterile water. After 14–21 days, the yeast grew out onto the coverslip and the slide was tested. The cover slip was gently removed with sterile forceps and placed on a clean microscopic slide with a drop of water or lactophenol cotton blue (Hardy Diagnostics, Santa Maria, CA) for microscopic observations. Phase contrast images were captured on an Olympus BX53 microscope with an Olympus DP25 camera and Olympus cellSens software. Differential interference contrast (DIC) images were generated on a Nikon Ti2-E inverted microscope with Nikon NIS Elements software. Cell morphological characters were measured with the Olympus cellSens or Nikon NIS Elements software.

Assimilation tests were performed in liquid media following the procedures described by (Kurtzman et al. 2011), in custom-made microplates (Nunc 96-Well U-Bottom plate, Thermo Fisher Scientific) and 5 mL tubes (Falcon, Corning) using the same standard set of substrates and with YT MicroPlates (BioLog) as described elsewhere (Greetham et al. 2014; Mašínová et al. 2018; Passer et al. 2019). Tubes and microplates with suitable aliquots of test cultures were incubated at 25 °C. Utilization of substrates was examined in PDA and incubated for 7 days. The change in coloration and oxidation and/or assimilation profiles of 94 different carbon sources were documented by visual examination. The clear positives and negatives were scored within 7 days of incubation, and delayed reaction (weak) was documented by examining the tetrazolium violet color change after 14 days of incubation. Further incubation and observation on 14th day did not yield any difference in results compared to those that were observed at day 7. Tubes were examined after 7, 14 and 21 days, and the growth (changing turbidity) was documented by visual examination.

### Preliminary identification based on ITS sequencing

DNA was extracted from 7-day grown yeast cultures on PDA incubated at 25 °C using the tissue LEV purification kit with Maxwell-16 MDx automated system (Promega, Madison, WI, USA). The ITS region was amplified employing polymerase chain reaction (PCR), using primers ITS 1F (5′-CTT GGT CAT TTA GAG GAA GTA A-3′) (Lai et al. 2007)*,* and Tw13 (5′-GGT CCG TGT TTC AAG ACG-3′) (Taylor and Bruns 1999). PCR conditions and sample preparation steps for sequencing are described elsewhere (Blachowicz et al. 2017)*.* The ITS sequences were also included in the subsequent MLSA-based analyses.

### Whole genome sequencing

Genomic DNA was isolated from 1 g wet biomass scraped from PDA plates, using a ZymoBIOMICS MagBead DNA kit (Zymo Research) and bead beating, followed by a Precellys homogenizer (Bertin) treatment. The quality of genomic DNA was verified by gel electrophoresis and quantified by spectrophotometric measurements (Nanophotometer NP80 Mobile, Implen GmbH). Library preparation was performed using the Illumina Nextera Flex Protocol (Illumina document # 1000000025416 v07). The initial amount of DNA for library preparation was quantified, and 5–12 cycles of PCR amplification were carried out to normalize the output depending on the input DNA concentration. The amplified genomic DNA fragments were indexed and pooled in a 384-plex configuration with dual-index adapters. WGS was performed on a NovaSeq 6000 S4 flowcell PE 2 × 150 base pairs (bp) platform with a paired-end module. The data was filtered with NGS QC Toolkit v2.3 (Patel and Jain 2012)*,* for high-quality (HQ) vector and adaptor-free reads for genome assembly (cutoff read length for HQ, 80%; cutoff quality score, 20). The genomes for the two novel yeast species were reassembled using the Automatic Assembly For The Fungi (AAFTF v 0.3.3) pipeline (Stajich and Palmer 2019), as follows: (i)The Illumina sequencing reads underwent trimming via the BBDuk module within the BBMap software suite (v 38.95*)* (BBMap: A Fast, Accurate, Splice-Aware Aligner (Conference) OSTI.GOV); (ii) BBMap was employed to eliminate any contaminant reads, and (iii) the resulting high-quality reads were assembled using SPAdes v3.15.4 (Bankevich et al. 2012). (iv) Contaminant contigs were identified and removed, using nucleotide Basic Local Alignment Search Tool (BLASTn) (Johnson et al. 2008); (v) We purged duplicated contigs using minimap2 (Li 2018). Final assemblies were polished, using the Pilon v1.24 software (Walker et al. 2014); (vii) The final scaffolds were sorted by length and the fasta headers renamed for further annotation. The assembly quality was verified with QUAST 5.1.0 (Gurevich et al. 2013) and completeness using BUSCO and its basidiomycota_odb10 database (Seppey et al. 2019).

### Genome annotation and comparative genomic analyses

As described in Chander et al. (2022b), the funannotate v1.8 pipeline (Palmer and Stajich 2020) for fungal genome annotation was used. Masked repetitive DNA content were identified using TANTAN (Frith 2011) using the funannotate mask command. The funnanotate predict command was used for gene content prediction, via both evidence-based and ab initio (i.e. structure-based) prediction pathways. The basidiomycota_odb10 BUSCO database (Simão et al. 2015) was used to find conserved gene models for training the ab initio predictors Augustus (Seppey et al. 2019), glimmerhmm (Delcher 1999), and snap (Korf 2004). The GeneMark-ES was also evoked with the option for fungal genomes (Lomsadze 2005). Weighted consensus gene structure was obtained via EVidenceModeler (Haas et al. 2008) using weigh = 2 in the case of Augustus HiQ models and weight = 1 otherwise. Genes of length < 50aa and genes identified as transposable elements were filtered out of the dataset. tRNAscan-SE was used to predict tRNAs (Chan and Lowe 2019). Finally, the funannotate annotate function was used to annotate genes using Eggnog (Huerta-Cepas et al. 2019), Pfam (Mistry et al. 2021), carbohydrate-degrading enzymes (CAZYme) (Drula et al. 2022), Interproscan (Blum et al. 2021), Gene Ontology (GO) terms (Gene Ontology Consortium 2004), MEROPS (Rawlings et al. 2018), and Secreted proteins (Choi et al. 2010). Biosynthetic gene clusters (BGCs) were predicted in the two yeast genomes via fungiSMASH, the fungal-genome-specific version of antiSMASH (Blin et al. 2021). We next run the funannotate compare function in 7 *Naganishia* species (which ones) and in 5 *Cystobasidium* (which ones), to look for genomic trends of the above protein categories in those two groups of fungi. Next, we ran CAFE5 to infer changes in the size of Interpro gene families regarding their phylogenetic distribution using the gene birth and death procedure to infer gene gain and loss of specific yeast phenotypes. Assembled genomes and its respectively annotations were deposited at the National Center for Biotechnology Information (NCBI; Chander et al. 2022a).

### Multi-locus phylogenetic analyses

Following recent multi-gene analyses of basidiomycetous yeasts (Liu et al. 2015; Wang et al. 2015b), MLSA was used to obtain solid placement of the two yeast strains. The gene sequences of all available *Naganishia* strains were retrieved from NCBI, except for *N. tulchinskyi* (Bijlani et al. 2022) and FJI-L2-BK-P3^T^ strains, which were obtained from using blast tools (Additional file 1: Table S1). Likewise, the gene sequence of all available *Cystobasidium* strains were retrieved from NCBI, except for the FKI-L6-BK-PAB1^T^ strain, which was obtained from its WGS during this study (Additional file 1: Table S2). Phylogenetic analyses were carried out using seven genes (ITS*,* LSU*,* SSU*, RPB*1, *RPB*2, *CYTB*, and *TEF1*) with all available *Naganishia* (*n* = 20) and *Cystobasidium* species (*n* = 21). The individual gene sequences for *Cystobasidium* and *Naganishia* datasets were aligned separately using the R package DECIPHER (Wright 2020). Briefly, sequences from a fasta file were extracted and loaded using *ReadDNAStringSet* function. The *removeGaps* argument was set to "all" to remove all gaps in the input sequences. For this reason, *OrientNucleotides* function was used to reorient sequences by changing their directionality and/or complementarity to match specified reference sequences in the same set. By default, the first sequence with maximum width was used as a reference sequence. Character string indicating the allowed reorientation of non-reference sequences was set to "*both*" (for reverse complement). The algorithm works by finding the *k-mer* distance between the reference sequence. The more the sequences look similar, smaller is the evolutionary distance. Numeric threshold giving the decrease in *k-mer* distance required to adopt the alternative orientation was set to 0.05. *AlignSeqs* function was performed for the generation of the alignment. The profile-to-profile method aligns a sequence set by merging profiles along a guide tree until all the input sequences are aligned. Guide tree was automatically constructed based on a distance matrix of shared *k-mers* using the setting *guideTree* = NULL. *iterations* = 2 and *refinement* = 1 were set to specify the number of iteration steps to regenerate the guide tree based and the re-alignment. Thus, the best alignment was chosen based on its sum-of-pairs score. Construction of phylogenetic trees utilizing the seven gene sequences listed above for MLSA was performed via IQ-TREE v 2.1.1 using the concatenation approach (Minh et al. 2020). ModelFinder approach was used for the evaluation of the best DNA substitution model (Kalyaanamoorthy et al. 2017). Ultrafast bootstraps and SH-like approximate likelihood ratio test (SH-aLRT) were assumed for branch support (Anisimova et al. 2011). The tree topologies were then visualized with the Interactive Tree Of Life v5 (https://itol.embl.de/).

### Whole genome-based phylogenetic analyses

Phylogenomic trees based on universal barcodes for fungi were constructed following the PHYling pipeline (GitHub - stajichlab/PHYling_unified: Unified PHYling pipeline for species tree building from annotated genomes; see https://github.com/stajichlab/AAFTF and https://github.com/nextgenusfs/funannotate for assembly and annotation steps). The protein models representing the *Tremellomycetes* class (for phylogenomic analysis of FJI-L2-BK-P3^T^) and the *Cystobasidiales* order (for phylogenomic analysis of FJI-L2-BK-P3^T^) were downloaded from the Mycocosm portal (Grigoriev et al. 2014)*.* Additional unannotated genomes representing the *Cystobasidium* and *Naganishia* species were downloaded from NCBI and submitted to the same gene prediction papiline described above (See Genome Annotation and Comparative Genomic Analyses section). We used the hmmsearch tool from HMMER v3.3.2, to compare protein sequences to the fungi_odb10 database, a collection of benchmarking protein sequences that are conserved across a wide range of fungal species, and identify potential homologous sequences (Simão et al. 2015). The tool utilizes a statistical model of protein sequence conservation to identify the degree of similarity between the query sequence and the protein sequences contained in the database. To create the protein alignments, the HMMER v3.3.2 hmmbuild function was employed. Spurious positions were purged with the ClipKIT v 1.3.0 tool, using the smartgap function. We then generated individual protein Maximum Likelihood (ML) phylogenetic trees employing IQ-TREE2 using partitions from each protein marker; the best protein model was calculated individually utilizing standard model selection (-m TEST option) (Kalyaanamoorthy et al. 2017). Tree reconstruction was performed with the supermatrix approach (Chernomor et al. 2016) and branch fidelity was tested using SH-aLRT (Anisimova and Gascuel 2006) and ultrafast bootstrap methods (Hoang et al. 2018). The final tree for each species was visualized with FigTree v 1.4.4 software.

### UV-C resistance

The evaluation of UV-C resistance was carried out via methods described previously (Blachowicz et al. 2019). Briefly, yeast cells (n = 3 species) were counted using a hemocytometer (Double Neubauer Counting Chamber, Hausser Scientific, Horsham, PA, United States) from 7-day-old cultures grown at 25 °C on PDA. Cell suspensions were diluted in molecular grade sterile water (Fisher Scientific, Waltham, MA, United States) and ∼ 2 × 10^4^ cells/mL were added to precision-cleaned aluminum coupons followed by drying overnight at room temperature in a bio-hood (Chander et al. 2022a, b). Coupons with dried yeast cells were placed in plastic Petri dishes, without a lid, and exposed to UV-C using a Crosslinker CL-1000 (UVP Inc). The lamp was placed above the sample with an exposure time required to produce the following doses: 0, 500, 1000, 2000 and 3000 J/m^2^. The dose rate was 100 μW cm^−2^. After exposure to UV-C, 100 μL of 10% sterile polyvinyl alcohol (PVA) was applied on each coupon and dried at 37 °C for 60 min. Dried PVA along with yeast cells was peeled using sterile forceps and added to 1 mL of molecular grade sterile water. The PVA extraction step was repeated twice. Once PVA was dissolved, serial dilutions were prepared and plated on PDA in quadruplicates. Colony forming units (CFUs) were counted after 7 days of incubation at 25 °C.

## TAXONOMY

***Naganishia kalamii*** K. Venkateswaran, P. Leo & N. K. Singh**, sp. nov.**

MycoBank: MB 846830.

*Etymology*: After Avul Pakir Jainulabdeen Abdul Kalam, an Indian aerospace scientist in whose honor the species is named.

*Diagnosis: N. kalamii* can be readily differentiated from its phylogenetically closest relative *N. albida* by the growth at 30 °C and 35 °C, whereas *N. albida* did not exhibit growth at temperatures higher than 25 °C. Since *N. kalamii* assimilate imidazole and exhibited growth at 10% NaCl, these tests can be used to differentiate *N. kalamii* from other member of the *N. albida* clade.

*Type:* **USA**: *California*: Pasadena, 34.1478° N, 118.1445° W, JPL-SAF cleanroom floor, where Mars 2020 mission components were assembled, isolated 17 Apr 2018, *Kasthuri Venkateswaran* (NRRL 64466 – holotype stored in a metabolically inactive state as a lyophilized culture; DSM 115730 – isotype cryopreserved in a metabolically inactive state).

**Fig. 1:**
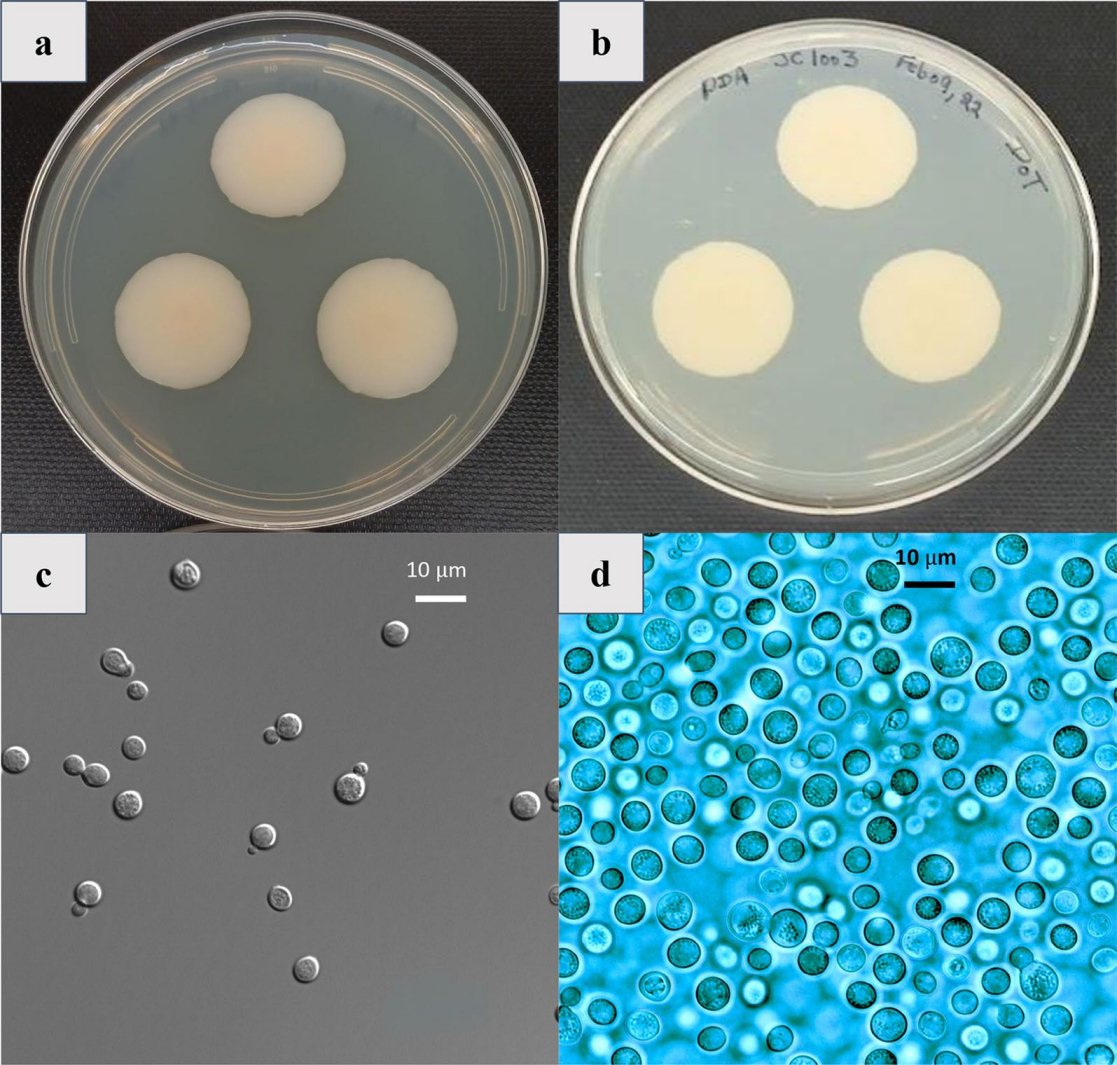
*Naganishia kalamii* FJI-L2-BK-P3^T^ **a** Colonies on PDA are cream colored, round, and 30 mm in diameter. **b** Reverse view of colonies on PDA after 14 days of incubation at room temperature (24 °C). **c** Cells are not observed to cluster or form pseudohyphae. **d** Small vacuoles are observed, cells are round, vary widely in size, and reproduce through budding. (Scale bar = 10 µm)

*Description:* After 5 d at 25 °C on PDA, streak culture white to cream-colored (becoming brownish upon aging), viscous to butyrous with a smooth surface. Margins smooth and entire, profile raised (Fig. 1a). Cells round, ovoid to ellipsoidal (Fig. 1a, b), 4.8 ± 0.97 μm, occurring singly or in pairs, proliferating by polar budding (Fig. 1c, d). Pseudohyphae true hyphae, ballistoconidia, and sexual morph not observed. Glucose not fermented. D-glucose, D-galactose (weak and slow), D-xylose, L-arabinose, sucrose, maltose, methyl-α-D-glucoside (weak), lactose (weak), raffinose, melezitose (weak), starch, D-glucitol, D-mannitol, galactitol, myo-inositol, D-gluconate, D-glucoronate, succinate (weak), ethanol, D-glucarate, and L-malic acid. No growth on L-sorbose, D-glucosamine, D-ribose, D-arabinose, L-rhamnose, α,α-trehalose, salicin, melibiose, inulin, glycerol, erythritol, ribitol, L-arabinitol, 5-keto-gluconate, D-galacturonate, lactate, citrate, methanol, and L-tartaric and D-tartaric acids.

Positive growth observed on potassium nitrate, and sodium nitrite, L-lysine, and imidazole, but not on ethylamine, cadaverine, creatine, creatinine, glucosamine, imidazole, and D-tryptophan. Urea hydrolysis and Diazonium Blue B reaction positive. Also shows positive growth on 5% glucose medium with 5%, 8% and 10% (weak) NaCl (w/v). Growth absent on 50% and 60% (w/w) glucose-yeast extract agar. Starch-like compounds not produced. Growth occurs at 4, 25, 30, and at 35 °C and not at 37 °C on PDA.

Differential biochemical characteristics of *N. kalamii* with other closely related *Naganishia* species are shown in Additional file 1: Table S3.

*Molecular characteristics* (holotype): nucleotide sequences of ITS and LSU (D1/D2 domains) rRNA are deposited in NCBI/EMBL under accession numbers OQ107186 and OQ107185, respectively.

*Ecology:* The only known strain was isolated from the cleanroom floor at JPL-SAF.

***Cystobasidium onofrii*** K. Venkateswaran, P. Leo & N. K. Singh, **sp. nov.**

MycoBank MB: 844481.

*Etymology*: Named in honour of Silvano Onofri, mycologist at the University of Tuscia, Viterbo, Italy, and a leading scientist in research on black yeasts and similar fungi in extreme habitats.

*Diagnosis*: Darker pink to orange-colored colonies and the ability to grow in medium supplemented with 5% NaCl are useful to differentiate *C. onofrii* from other recognized species of *Cystobasidium*.

*Type:* **USA**: Florida: Cape Canaveral, 28° 23′ 48.6″ N, 80° 36′ 20.4″ W, KSC-PHSF isolated from cleanroom floor where Mars 2020 mission components were assembled, 12 Jun 2018, *Kasthuri Venkateswaran* (NRRL 64426—holotype stored in a metabolically inactive state as a lyophilized culture; DSM 114625—isotype metabolically inactive cryopreserved culture).

*Description:* After 7 d at 25 °C on PDA, streak culture pink to orange (becoming brownish upon aging), butyrous with a smooth surface. Margins smooth and entire, and the profile raised (Fig. 4a, b). Cells round, ovoid to ellipsoidal, 4.1 ± 0.97 μm, occurring singly or in pairs, proliferating by polar budding (Fig. 4c, d). Pseudohyphae, true hyphae, ballistoconidia, and sexual morph were not observed.

Sugars not fermented: d-glucose, d-galactose (sometimes weak), l-sorbose (sometimes weak), d-xylose, l-arabinose, sucrose, a,a-trehalose, lactose, melezitose, gentiobiose, inulin (sometimes weak), starch (weak), glycerol, ribitol, d-glucitol (weak), d-mannitol (weak), 5-keto-d-gluconate, d-gluconate, d-glucuronate, ethanol, methanol, d-glucarate and l-malic acid are assimilated. No growth on salicin, melibiose, raffinose, l-arabinitol, galactitol, myo-inositol, d-galacturonate, l-tartaric acid and d-tartaric acid.

No growth observed on potassium nitrate and sodium nitrite and ethylamine, but positive growth on l-lysine exhibited. Urea hydrolysis and Diazonium Blue B reaction positive. Also shows positive growth on 5% glucose medium with 5%, 8% and 10% (weak) NaCl (w/v). Growth absent on 50% and 60% (w/w) glucose-yeast extract agar. Starch-like compounds not produced. Growth occurs at 4, 25, and 30 °C and not at 35 °C.

Differentiating biochemical characteristics from other closely related *Cystobasidium* species are shown in Additional file 1: Table S5. Among the *Cystobasidium* species, *C. tubakii* and *C. pallidum* were phylogenetically related but differ in assimilation of l-arabinose and cellobiose, and growth in the presence of cycloheximide.

*Molecular characteristics* (holotype): nucleotide sequences of ITS and LSU (D1/D2 domains) rRNA deposited in NCBI/EMBL under accession numbers MT704910 and OQ107184, respectively.

*Ecology:* The only known strain was isolated from the cleanroom floor at the Kennedy Space Center (KSC-PHSF).

## RESULTS

### Naganishia kalamii

#### Molecular phylogeny

**Fig. 2:**
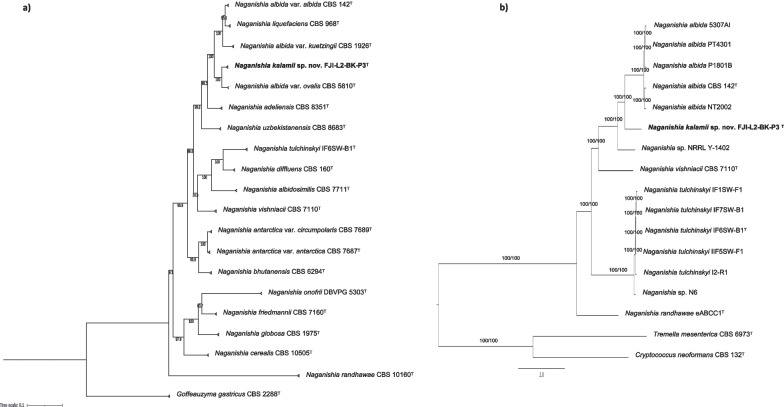
**a** Maximum likelihood phylogenetic tree, based on concatenated seven gene sequences (*ITS, LSU, SSU, CYTB, RPB1, RPB2, TEF1*), extracted from WGS of the respective type strains (superscript T) or from the available genomes, showing the phylogenetic relationship of *N. kalamii* with closely related members of *Tremellomycetes*. The gene fragment lengths in base pairs (bp) are given in parenthesis: ITS (621 bp); LSU (611 bp); SSU (517 bp); *CYTB* (339 bp); *RPB1* (317 bp); *RPB2* (1282 bp); *TEF1* (1084 bp). Bootstrap values from 1000 replications are shown at branch points. Bar = 0.1 substitution per site. GenBank accession numbers of the sequences are listed in Additional file 1: Table S1. **b** WGS Maximum likelihood phylogeny of *Naganishia kalamii* reconstructed using a concatenated alignment of universally distributed yeast genes. Branch fidelity used two different approaches, Ultrafast bootstraps and Gene Concordance Factors, which were added next to its corresponding branches

*Naganishia kalamii* belongs to *Basidiomycota*, class *Tremellomycetes*, and order *Filobasidiales*. ITS sequence similarity of the strain FJI-L2-BK-P3^T^ with the majority of *Naganishia* species was > 98.5% suggesting that the ITS marker alone may not be useful to identify *Naganishia* species (see also Scorzetti et al. 2002; Fonseca et al. 2011). Furthermore, combined sequence analyses of both ITS and LSU fragments placed *N. kalamii* in the *N. albida* clade, in a distinct position next to *N. albida* var. *ovalis* CBS 5810^T^ (two substitutions in the LSU). Although the *N. albida* clade is represented in the literature, collections and databases by an array of strains showing highly variable physiological and biochemical characteristics, the pair-wise distances matrix clearly shows the difficulties to discriminate the members of *N. albida* clade when only ITS and LSU markers were used (Additional file 2: Fig. S2). This problem was highlighted in the comments to the species by Fonseca et al. (2011), but in 2021 two *Naganishia* species were described largely based on sequence dissimilarity in the clade (Kachalkin et al. 2019; Tan et al. 2021). However, it is important to note that the analyzed protein-coding (*CYTB*, *TEF1, RPB1* and *RPB2*) individual phylogenetic trees of gene fragments other than SSU (invariable for the whole clade), LSU and ITS exhibited higher resolution in differentiating the *Naganishia* species (Additional file 2: Fig. S3–S7). Furthermore, seven-loci phylogenetic tree (Fig. 2a) convincingly showed that FJI-L2-BK-P3^T^ occupies a distinct position in the clade with high bootstrap support (100%). The novel species *N. kalamii* is most closely related to *N. albida* var. *ovalis* CBS 5810^T^, *N. albida* var. *kuetzingii* CBS 1926^T^ (type of *Cryptococcus kuetzingii*), *N. albida* var. *albida* CBS 142^T^, *N. liquefaciens* CBS 968^T^ and *N. adeliensis* CBS 8351^T^ (Fig. 2a). Another species *N. nivalis* was not included in the MLSA-analysis because protein-coding sequences were not available for the analysis. The tree based on conserved genes from the *N. kalamii* FJI-L2-BK-P3^T^ genome (Fig. 2b) showed that *N. kalamii* is phylogenetically distinct from *N. albida* var. *albida* CBS 142^T^ and *Naganishia* sp. NRRL Y-1402 that was earlier misidentified as *N. albida* var. *albida*.

#### Genomic characteristics of *N. kalamii* FJI-L2-BK-P3^T^ and comparative genomic analysis

**Fig. 3:**
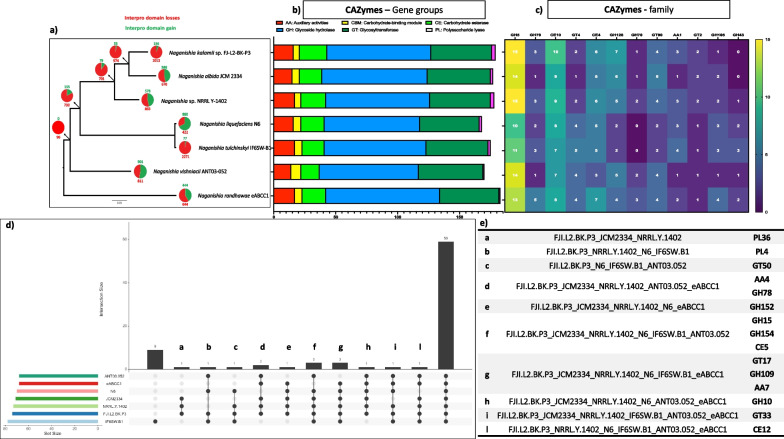
Comparative genomic analyses. **a** Phylogenetic tree showing gain and loss of Interpro domains of *Naganishia kalamii* and other *Naganishia* species. **b**–**c** Bar plot showing CAZYme group and family domains among the *Naganishia* species. **d**–**e** UpSet plot showing the distribution (shared and unique) of the CAZYme family terms among the *Naganishia* species and the relative table

The estimated genome size of *N. kalamii* FJI-L2-BK-P3^T^ is 27.86 Mb and assembled into 478 scaffolds (N50 = 190,774, L50 = 38) with a largest scaffold as 1,000,601 bp. The GC content is 53.9% and the raw sequences are submitted with an accession #: JAKLLX000000000. A total of 8,085 genes were identified, divided into 7902 mRNAs and 183 tRNAs. We have found 1,904 GO annotated and 6007 Eggnog terms, 2548 Interpro and 4783 Pfam domains, 177 CAZYmes, 219 MEROPS peptidases, 396 secreted proteins, and 1624 BUSCO orthologs. When compared with other *Naganishia* species, *N. kalamii* has the highest number of genes (8085) and 7902 proteins products (Additional file 1: Table S4). Among the seven species of *Naganishia* analyzed, three species belonging to the *N. albida* clade (*N. kalamii*, *N. albida* JCM 2334 and NRRL Y-1402) had similar number of predicted genes and proteins, of which *N. kalamii* exhibited the highest number of secreted proteins (Additional file 2: Fig. S8) as well the number of peptidases (MEROPS; Additional file 2: Fig. S9) but not CAZymes (Fig. 3). Lowest numbers of CAZymes terms were seen in *Naganishia* sp. N6 (168; deep sea isolate) and *N. vishniacii* ANT03-052 (170; Antarctic soil isolate), that were isolated from natural extreme environments.

Based on the detailed research in the MEROPS peptide database, 219 different membrane associated peptidases were identified within the families: aspartic (10), cycteine (52), metallo (76), serine (62), threonine (17) and protease inhibitors (2). Serine peptidase, S9 prolyl oligopeptidase (S09X subfamily), and the prolyl aminopeptidases (S33) were the most abundant families detected in the *Naganishia* species. Moreover, the metallo protease thimet oligopeptidase (M03A), pitrilysin (M16A), aminopeptidase N (M01) and the serine protease d-Ala-d-Ala carboxypeptidase B (S12) were found to be overrepresented in the genome of *N. kalamii* (Additional file 2: Fig. S9).

Detailed analysis into CAZy database revealed 49 genes associated with assembly (GT: glycosyltransferase) and 109 (84 GH: glycoside hydrolase, 3 PL: polysaccharide lyase, 22 CE: carbohydrate esterase) involved in the breakdown of carbohydrate complexes (Fig. 3). In addition, 16 genes coding for initial lignin degradation (AA: auxiliary activities) and 5 involved in carbohydrate-binding module (CBM) were also predicted. According to genome-wide comparison, *N. kalamii* shared 59 CAZy families with other *Naganishia* species analyzed. No unique CAZy families have been recorded for *N. kalamii*. However, the polysaccharide lyase family 36 (PL36) was shared only among the members of the *N. albida* clade. GH5 and CE10 were the largest family (both with 15 predicted genes in *N. kalamii*) shared also by the other *Naganishia* members. GH5 consisted of genes related to cellulolytic activity while genes encoding CE10 family members act on non-carbohydrate substrates. Genes involved in the degradation of endogenous fungal cell wall polysaccharides (GH128) and lignin (AA1) were also detected.

Since survival of yeasts on UV-C exposure was performed during this study, Pfam and Interpro terms associated with dehydration, desiccation, rehydration and UV resistance have been investigated (Additional file 2: Fig. S10). Results did not reveal significant differences in Pfam terms among the *Naganishia* species analyzed. Protein tyrosine and serine/threonine kinase (PF07714), WD domain, G-beta repeat (PF00400), Ras family (PF00071), Cytochrome P450 (PF00067), Ankyrin repeats (PF12796) and ABC transporter (PF00005) were the most presented Pfam terms in all the species. Heat shock protein 70 and dehydration stress-response Pfam terms were also commonly present in all the species analyzed. The psychrophilic *N. vishniacii* ANT03-052 showed presence of genes involved in raffinose synthesis (PF05691).

The Interpro analysis filtered for UV resistance terms for *N. kalamii* showed the absence of protein directly involved in the synthesis of carotenoids (IPR004294). *N. kalamii* also lack the UV radiation resistance protein (IPR018791), short coiled – coil protein (IPR019357), and the XPG conserved site (IPR019974) involved in the catalytic mechanism of DNA excision repair, but XPG, N-terminal (IPR006085) and XPG/Rad2 endonuclease (IPR006084) are present, both not conserved residues. The only strain exhibiting all the terms involved in the UV resistance was Antarctic *N. vishniacii* ANT03-052 strain.

The profile of fungal-specific transcriptional factors was compared among the *Naganishia* species (Additional file 2: Fig. S11). Fungal Zn(2)-Cys(6) binuclear cluster domain (IPR001138), fungal-specific TF domain (IPR007219), basic region leucine zipper 2 (IPR004827) and bZIP TF 1 (IPR004827) were commonly enriched in all the species considered. However, it was not prominent in *N. kalamii* when compared to the Antarctic strain *N. vishniacii* ANT03-052 and avian guano in South Africa strain *N. randhawae* eABCC1.

CAFE5 analysis revealed that *N. kalamii* has experienced an expansion of 10 Interpro domain categories while a reduction of 43 Interpro domains (*p* < 0.05) was witnessed (Fig. 3a). The GO term conversion of the acquired Interpro domains are related to protein N-linked glycosylation and inositol phosphate dephosphorylation biological processes. In contrary, the contracted GO terms are related to regulation of DNA-templated transcription, regulation of transcription by RNA polymerase II, protein folding, proteolysis, ubiquitin-dependent protein catabolic process, intracellular protein transport and vesicle-mediated transport. This suggested that *N. kalamii* have lost domains related to the control of transcription as well as protein degradation, folding and transport.

**Fig. 4:**
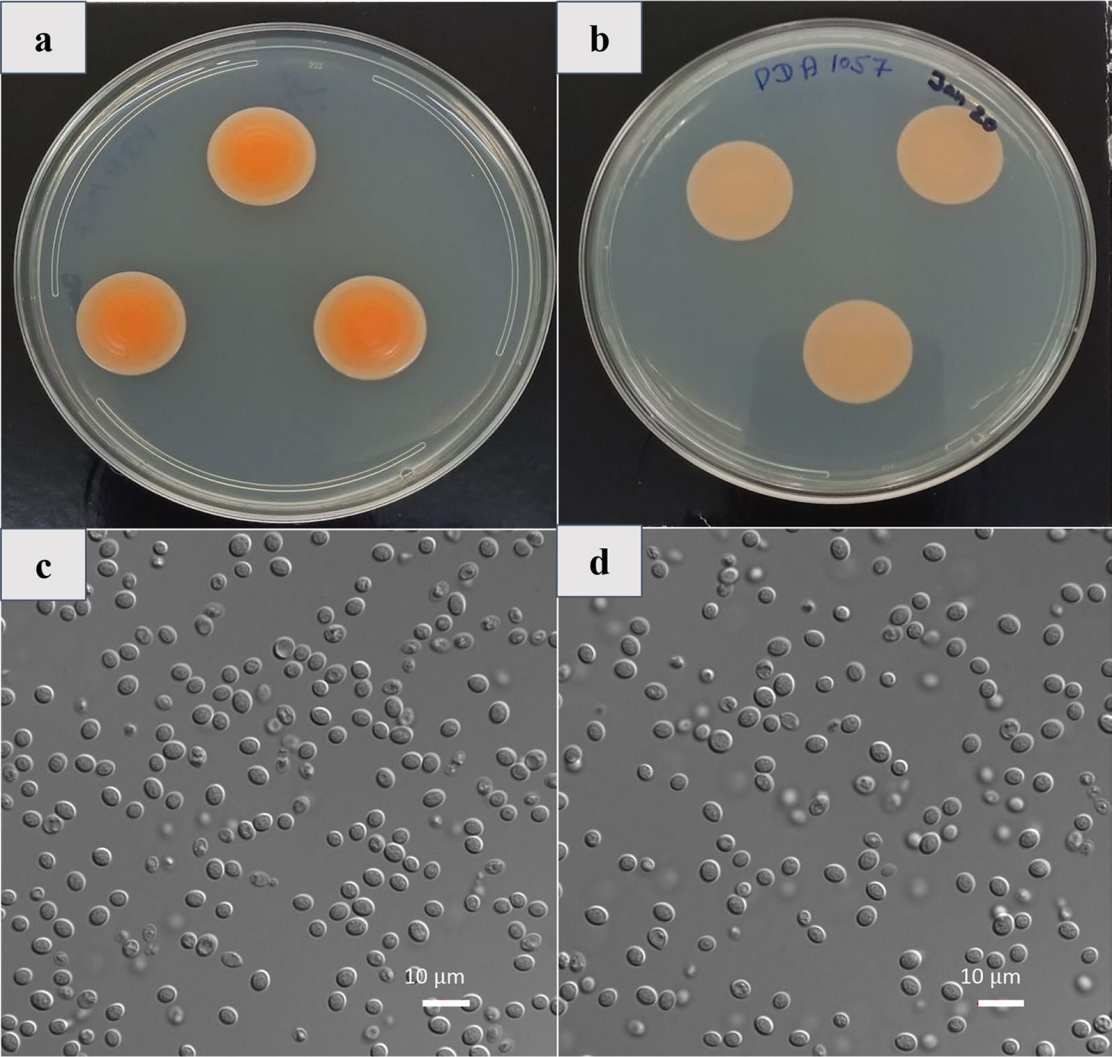
*Cystobasidium onofrii* FKI-L6-BK-PAB1^T^. **a** After 21 days of incubation at room temperature, the colonies on PDA are bright orange, round, and 20 mm in diameter, and are light orange. **b** Reverse view of colonies on PDA. **c**–**d** Individual yeast cells are ovoid in shape and observed to reproduce through budding. (Scale bar = 10 µm)

Six biosynthetic gene clusters (BGCs) were predicted in the genome of *N. kalamii* FJI-L2-BK-P3^T^, five of which do not match with any known BGCs. These included three non-ribosomal peptide synthase-like (NRPS-like) clusters, one cluster encoding for a Type III polyketide synthase, and two clusters encoding for terpene synthesis, one of which was identified as the BGC for the synthesis of clavaric acid. Similar sets of BGCs were predicted for the genomes of *Naganishia* sp. strain N6 and *N. albida* JCM 2334, with two differences: one, neither of the terpene synthesis clusters were a match for clavaric acid in the genome of *N. albida* JCM 2334; and two, *Naganishia* sp. strain N6 was predicted to have a NRPS cluster in place of one of the three NRPS-like clusters (Fig. 4).

### Cystobasidium onofrii

#### Phylogenetic placement

**Fig. 5:**
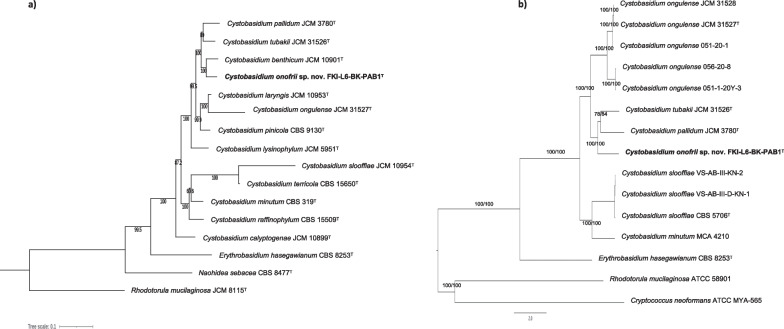
**a** Maximum likelihood phylogenetic tree, based on concatenated seven gene sequences (ITS, LSU, SSU, *CYTB*, *RPB1*, *RPB2*, *TEF1*), extracted from WGS of the respective type strains (superscript T) or from the available genomes, showing the phylogenetic relationship of *C. onofrii* with closely related members of *Cystobasidiomycetes*. The gene fragment lengths in base pairs are as given in Fig. 2. Bootstrap values from 1000 replications are shown at branch points. Bar = 0.1 substitution per site. GenBank accession numbers of the sequences are listed in Additional file 1: Table S2. **b** WGS Maximum likelihood phylogeny of *Cystobasidium onofrii* reconstructed using a concatenated alignment of universally distributed yeast genes. Branch fidelity used two different approaches, Ultrafast bootstraps and Gene Concordance Factors, which were added next to its corresponding branches

This novel yeast belongs to the phylum *Basidiomycota*, class *Cystobasidiomycetes*, and order *Cystobasidiales.* According to the phylogenetic analysis based on ITS and LSU sequences, *C. onofrii* is distantly related to *C. benthicum* JCM 10901^T^ (16 base substitutions and 6 gaps, 97.01% of similarity in ITS and 5 base substitutions, 99.22% in LSU). The similarity of *C. onofrii* with other *Cystobasidium* species are shown in Additional file 1: Table S6 and the two ribosomal genetic markers were found to be good to discriminate *Cystobasidium* species. The individual phylogenetic trees using ITS*,* LSU, SSU*, CTYB, TEF1, RPB1, RPB2,* (Additional file 2: Fig. S12–S18; Additional file 1: Table S6) were generated followed by a concatenated seven-loci phylogenetic tree (Fig. 5a) for *Cystobasidiales* species. All the seven loci investigated exhibited good resolution in delimiting the analysed *Cystobasidium* species. Based on the MLSA tree, strain FKI-L6-BK-PAB1^T^ constitutes a well-supported clade with *C. benthicum, C. tubakii,* and *C. pallidum,* each of the species representing a well-separated lineage (Fig. 5a). The WGS-based analysis further supported a distinct phylogenetic position of the strain FKI-L6-BK-PAB1^T^ (Fig. 5b) and formed a triad along with *C. tubakii* 9A-1–0 and *C. pallidum* JCM 3780.

#### Genomic characteristics of *C. onofrii* FKI-L6-BK-PAB1^T^and comparative genomic analysis

The estimated genome size of *C. onofrii* FKI-L6-BK-PAB1^T^ is 20.99 Mb and assembled into 162 scaffolds (N50 = 616,502, L50 = 10) with a largest scaffold as 3,147,288 bp. The GC content is 48.8% and the raw sequences were submitted with an Accession#: JAKLNA000000000. The final annotation of the *C. onofrii* yielded a total of 6889 genes, divided into 6831 mRNAs and 58 tRNAs. The genome annotation revealed 2934 GO and 5820 Eggnog terms, 3930 Interpro and 4803 Pfam domains, 172 CAZYmes, 221 MEROPS peptidases, 335 secreted proteins, and 1540 BUSCO orthologs.

**Fig. 6:**
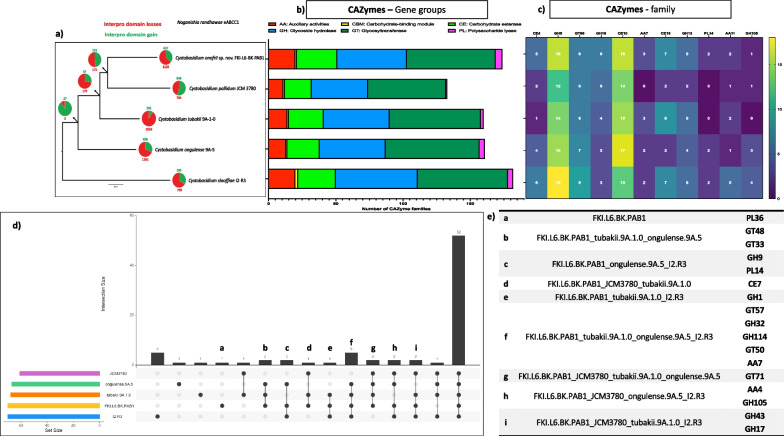
Comparative genomic analyses. **a** Phylogenetic tree showing gain and loss of Interpro domains of *Cystobasidium onofrii* and other *Cystobasidium* species. **b**–**c** Bar plot showing CAZYme group and family domains among the *Cystobasidium* species. **d**–**e** UpSet plot showing the distribution (shared and unique) of the CAZYme family terms among the *Cystobasidium* species and the relative table

Comparative genome analyses of *Cystobasidium* species showed that *C. onofrii* has the second highest number of genes (6889) and predicted protein products (6831) after *C. slooffiae* (Additional file 1: Table S7). The genomic annotations revealed that *C. onofrii* FKI-L6-BK-PAB1^T^ secreted larger number of proteins and second to *C. slooffiae* among the *Cystobasidium* species (Additional file 2: Fig. S19). Similar trend is observed for the number of secreted proteins that are peptidases (Additional file 2: Fig. S20), CAZymes (Fig. 6), and MEROPS classes. However, no significant changes in the number of each group have been observed. Among the CAZymes of *C. onofrii*, 30 genes associated with assembly of glycosyltransferase (GT) and 87 genes involved in the breakdown of carbohydrate complexes (52 GH: glycoside hydrolase, 5 PL: polysaccharide lyase, 30 CE: carbohydrate esterase) were predicted. In addition, 20 genes coding for initial lignin degradation (AA: auxiliary activities) and 1 involved in carbohydrate-binding module (CBM) were also noticed. According to genome-wide comparison, *C. onofrii* shared 52 CAZy families with the other 4 *Cystobasidium* species while PL Family 36 (PL36) was unique in *C. onofrii* (Fig. 6). GH5 and CE10 were the largest family (both with 16 predicted genes in *C. onofrii*) shared also by the other *Cystobasidium* members. GH5 presents genes related to the degradation of cellulose while genes encoding CE10 family members act on non-carbohydrate substrates. CE family 16 is another family well represented in *C. onofrii* along with *C. tubakii* and *C. slooffiae.*

Since *C. onofrii* FKI-L6-BK-PAB1^T^ strain could utilize several organic substrates, a search in the MEROPS peptide database was performed in order to survey the genome for membrane associated peptidases that could initiate the degradation of complex protein molecules. In total, 221 different peptidases were identified, which were classified within the families: aspartic (13), cycteine (48), metallo (68), serine (76), threonine (19), protease inhibitors (2) and mixed peptidase (1). Serine and metallo peptidase are the predominant families in all the *Cystobasidium* species. In *C. onofrii* S9 prolyl oligopeptidase (S09X subfamily) and the prolyl aminopeptidases (S33) are the more abundant terms. S33 includes some hydrolases, which additionally degrade other substrates like lipids. The *exo*-keratinolytic enzymes M38 and the A01A subfamily members, are found abundantly in *C. onofrii* compare to the other *Cystobasidium* species (Additional file 2: Fig. S20).

Pfam and Interpro terms associated with dehydration, desiccation, rehydration and UV resistance have been investigated as stress parameters (Additional file 2: Fig. S21). Results did not reveal significant differences in Pfam terms among the *Cystobasidium* analyzed. Protein tyrosine and serine/threonine kinase (PF07714), WD domain, G-beta repeat (PF00400), Ras family (PF00071), Cytochrome P450 (PF00067), Ankyrin repeats (3 copies) (PF12796) and ABC transporter (PF00005) are the most presented Pfam terms in all the species.

Heat shock protein 70 and other Pfam terms for predicted proteins involved in the dehydration stress-response are also commonly present in all the *Cystobasidium* species analyzed. The Interpro analysis filtered for UV resistance terms, seen in the genome of *C. onofrii* of proteins related to the synthesis of carotenoids (IPR004294; IPR002060) and DNA-repair (eg. IPR006084). However, *C. onofrii* lacks the UV radiation resistance protein (IPR018791) and short coiled protein (IPR019357) but both of these proteins are present in the genome of the Antarctic *C. ongulense* and in *C. slooffiae* (Additional file 2: Fig. S21).

Fungal-specific transcriptional factors among the *Cystobasidium* species (Additional file 2: Fig. S22) showed presence of Fungal Zn(2)-Cys(6) binuclear cluster domain (IPR001138), Fungal-specific TF domain (IPR007219), Basic region leucine zipper 2 (IPR004827), and bZIP TF 1 (IPR004827) and are more prominent in *C. onofrii* (Additional file 2: Fig. S22), when compared with the strains isolated from natural extreme environments (Antarctic soil and deep sea).

Data from CAFE analysis of *Cystobasidium* species showed that *C. onofrii* has experienced an expansion of 31 Interpro domain categories while a reduction of 9 Interpro domains (*p* < 0.05) (Fig. 6a). The expansions were related to acetyl-CoA biosynthetic process from pyruvate, DNA repair, regulation of DNA-templated transcription, protein phosphorylation, nitrogen compound metabolic process, mono-atomic ion transport, phospholipid biosynthetic process, iron ion transmembrane transport, and positive regulation of transcription by RNA polymerase II. In contrary, the contracted GO terms were related to DNA repair, sulfate transport, and transmembrane transport.

A single BGC, an NRPS-like cluster of unknown function, was predicted in the genome of *C. onofrii* FKI-L6-BK-PAB1^T^. The genomes of neighboring species *C. tubakii* JCM 31526^T^ and *C. pallidum* JCM 3780^T^ also contained a single NRPS-like cluster, with an additional unknown cluster encoding for terpene synthesis predicted for the genome of *C. pallidum* JCM 3780^T^*.* The genome of *C. benthicum* was not available for comparison.

### Resistance to UV irradiation of the novel yeasts

**Fig. 7:**
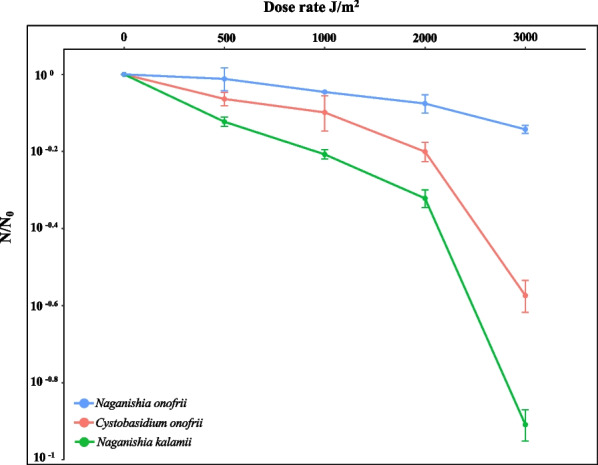
Survivability of various yeasts exposed to UV-C radiation (doses: 0, 500, 1000, 2000 and 3000 J/m^2^. UV-C survival rates calculated using formula: N/N_0_, where N is the number of cells surviving at any given dose and N_0_ the number of cells of original seed culture (~ 2 × 10^4^ cells/ml). The ratio of survival rates from three different species are plotted. The gray box shows the effect of desiccation and PVA treatment. The experiments were repeated twice

Survival rates of two novel species and the high-altitude cold-adapted *N. onofrii* exposed to UV-C are presented in Fig. 7. The cultivability of all three yeasts dramatically decreased due to desiccation before exposing them to UV-C irradiation conditions (Additional file 2: Fig. S23). The survival of yeasts after desiccation was 22% for *N. kalamii*, 23% for *C. onofrii*, and 33% for *N. onofrii*. This observed trend might be not only due to the desiccation but also the effect of polyvinyl alcohol (PVA) that was used to peel off the dried cells could not be ruled out. The number of cultivable cells after desiccation and PVA treatment were considered as baseline (N_0_) while calculating the effect of different doses of radiation (N).

In general, all three yeasts displayed a similar resistance pattern up to 2000 J/m^2^ with a gradual increase of mortality recorded with increasing radiation doses (Fig. 7). Among the three yeasts tested, only *N. onofrii* exhibited higher resistance (~ 5.5 × 10^3^ CFU at 2000 J/m^2^ and ~ 4.7 × 10^3^ CFU at 3000 J/m^2^ [*p* < 0.05], with 72% of conidia were cultivable after exposure to the highest dosage [3000 J/m^2^]). Even though *C. onofrii* (~ 2.1 × 10^3^ CFU) and *N. kalamii* (~ 2.9 × 10^3^ CFU) exhibited a linear decrease in cultivability up to 2000 J/m^2^, a significant decrease in viability was noticed at the 3000 J/m^2^ dosage for both *C. onofrii* (~ 5.6 × 10^2^ CFU; *p* < 0.05; Additional file 2: Fig. S23) and *N. kalamii* (~ 1.2 × 10^2^ CFU; *p* < 0.005; Additional file 2: Fig. S24). The pink-colored *C. onofrii* retained 27% of the cultivability after UV-C radiation exposure whereas the non-pigmented *N. kalamii* was the most sensitive, with only 13% of cultivable cells present after 3000 J/m^2^ of UV-C irradiation. Exposure experiments were repeated twice and showed the same trends.

## DISCUSSION

Microorganisms associated with NASA spacecraft assembly cleanrooms might hitchhike on spacecraft surfaces, contaminate extraterrestrial environments, or compromise the life-detection missions. Due to stringent maintenance and cleaning, SAFs can be considered as extreme environments since microorganisms found in these cleanrooms could withstand unfavorable conditions and survive low nutrient availability, desiccation, controlled temperature, etc. Indeed, bacteria and fungi isolated from these SAFs are poly-resistant and have been reported to withstand harsh and unfavorable conditions. Therefore, characterization of yeast species from NASA spacecraft assembly cleanrooms is essential.

The identification of yeasts has been shifted from phenotypic characterization to sequence-based identification and DNA-markers were utilized for defining their phylogenetic affiliation (Fonseca et al. 1999; Scorzetti et al. 2002). Since the 2000s, *Naganishia* species have been identified by considering differences among ribosomal gene sequences (ITS and partial LSU), which sometimes leads to ambiguous identification in subsequent revisions of the genus (Turchetti et al. 2015; Ruiz and Radwan 2021). Circumscription of the species *Cryptococcus albidus* has a long and complicated history of splitting and lumping using the ever-growing array of available tests and tools, including whole-cell protein patterns, DNA-DNA hybridization, and nucleotide sequencing (Fonseca et al. 2011). The species has long been known as a variable species and included, as varieties, yeasts that are presently placed in other genera and families of *Filobasidiales* (Fonseca et al. 2011; Liu et al. 2015). As the result of reclassification of the *C. albidus* species complex using LSU and later ITS sequences, many previous varieties were promoted to the taxonomic level of species, e.g. *Naganishia diffluens*, *N. globosa*, *N. liquefaciens*, *Solicoccozyma terrea*, *Solicoccozyma terricola*. Delineation of species using a single ribosomal gene region is not always reliable in basidiomycetous yeasts, because different lineages strongly vary in their rates of nucleotide substitution (Fell et al. 2000; Scorzetti et al. 2002). The case of the genus *Naganishia* is challenging as species were delimited based on very few substitutions in the two fragments, ITS and LSU. Based on ITS and LSU, the *albida* clade of the genus *Naganishia* currently include three varieties of *N. albida* (*N. albida* var. *albida* CBS 142^T^, *N. albida* var. *kuetzingii* CBS 1926^T^ (formerly *Cryptococcus kuetzingii*), *N. albida* var. *ovalis* CBS 5810^T^) and three other species (*N. nivalis* DBVPG 5706^T^, *N. liquefaciens* CBS 968^T^, *N. brisbanensis* BRIP 28244; Additional file 2: Fig. S1; Scorzetti et al. 2002; Kurtzman et al. 2011; Li et al. 2020). However, according to our results, combined sequence analysis of the LSU and ITS sequences, it is not possible to differentiate members of *N. albida* clade using the approach due to low discriminatory power and lack of bootstrap support (Additional file 2: Fig. S1). Our results suggest that the recently described *N. brisbanensis* (Tan et al. 2021) might be a taxonomic synonym of *Cryptococcus kuetzingii*, the species that have not been included in the original analysis by Tan et al. (2021). To improve as much as possible poor species resolution in the ITS-LSU tree, a seven-loci (MLSA) and WGS-based phylogenies were generated for the discrimination of *N. albida* clade members. The MLSA tree convincingly separated *N. kalamii* from the other members of the *N. albida* clade suggesting that it should be placed in a new species rather than keeping as a *N. albida* variety which would further increasing the heterogeneity of the species. Furthermore, our MLSA- and WGS-based trees suggest that all the varieties of the *N. albida* clade are likely to be independent species (Fig. 2a). However, the complete reclassification of *N. albida* must include (either as the seven-gene dataset, but preferably WGS) recently described *N. nivalis* and *N. brisbanensis*, the varieties *N. albida* var. *kuetzingii* and *N. albida* var. *ovalis*, as well as some present synonyms of the *N. albida*, namely *Torulopsis rotundata* and *Torulopsis nadaensis* (Fonseca et al. 2011). In addition, WGS-based phylogeny of the strains NY-1402, N6, and I2-R1 from public databases revealed that strain NY-1402 is potentially a novel species and two other strains (N6, isolated from deep-sea sediment and I2-R1, isolated from an ISS HEPA filter for a rodent experiment) should be assigned to *N. tulchinskyi*. These examples show that MLSA and WGS analyses are necessary to avoid misidentification (Taylor and Fisher 2003; Liu et al. 2015; Wang et al. 2015a, b; Vu et al. 2016).

*Naganishia* strains phylogenetically close to *N. kalamii* are well-adapted to cold environments. Extreme conditions might have a selective pressure leading to an increase in mutation rates, allowing microbial species to adapt to extreme conditions (Turchetti et al. 2015). Most of the *Naganishia* species (*N. vaughanmartiniae*, *N. onofrii*, *N. friedmannii*, *N. cerealis*, *N. randhawae*, *N. globosa, N. adeliensis*, *N. liquefaciens* and *N. albida*) are able to grow at low temperatures (4 °C) but only *N. kalamii* and *N. qatarensis* can grow at 35 °C. All the psychrophilic or psychrotolerant *Naganishia* species exhibit the presence of cold-adapted genes which might have allowed them to colonize cold environments such as Antarctica and the deep sea (Selbmann et al. 2005, 2013; Turchetti et al. 2015; Fotedar et al. 2018; Han et al. 2020; Segal-Kischinevzky et al. 2022). *N. qatarensis,* a halophilic yeast isolated from a warm hypersaline marine environment in Qatar, is reported to grow at 30–35 °C (Fotedar et al. 2018). Growth of *N. qatarensis* at lower temperature (~ 25 °C) has been reported but it took 8 weeks after incubation. Conversely, *N. kalamii* is able to grow in a temperature range of 15–35 °C after 7 days, and growth was noticed within 14 days when incubated at 4–10 °C. Similar to *Naganishia* species, the *C. onofrii* clade consists of three species that were also isolated from cold environments (Tsuji et al. 2017; de Menezes et al. 2019) and other members of the genus *Cystobasidium* (n = 12 species) are among the most common cold-adapted species (Buzzini et al. 2018). All members of the genus *Cystobasidium*, including *C. onofrii*, are psychrophilic or psychrotolerant, with optimum growth temperatures ranging from 15 to 20 °C.

Cryosphere harbors abundant microbial diversity, e.g. Antarctica, Greenland, cold lakes in Argentina, and deep sea (Buzzini and Margesin 2014). Cold-adapted yeast species are reported to survive while embedded in the ice layers of glaciers or in permafrost (Zucconi et al. 2012; Turchetti et al. 2013), finding micro-conditions that are isolated from the surrounding environments (Price 2000). Low nutrient availability is another limiting factor typical of Antarctic ecosystems. The oligotrophic Antarctic yeast *C. tubakii*, which closely related to *C. onofrii,* does not require amino acids and vitamins for growth, and is also able to grow at − 3 °C (Tsuji 2018). Since future space missions—for example, ESA JUpiter ICy moons Explorer and NASA Europa Clipper missions—are designed to explore the potential habitability of icy moons, the presence of cold-adapted yeasts should be monitored in the NASA cleanrooms. For future missions, monitoring psychrophiles, xerophiles and halophiles in SAFs is of particular interest because they may be capable of surviving inside or under the icy crust of Europa due to its extremely cold and saline environment (Marion et al. 2003). For instance, *C. lysiniphilum* and *C. slooffiae* are reported as halotolerant species (Mokhtarnejad et al. 2016). Similarly, *C. onofrii* shows moderately halophilic aptitude since it exhibits growth at 10% NaCl.

In general, SAF surfaces are decontaminated with reagents and also kept dry. Our genomic analysis reveals that both *Naganishia* and *Cystobasidium* species have predicted proteins (Pfam and Interpro terms) associated with dehydration and desiccation stress-response. Halotolerance is another adaptation required for yeasts typically active in hyper-arid regions, and so the halophilic traits detected in the genomes of these species also likely contributes to their survival in the cleanroom environment (Buzzini and Margesin 2014; Logan et al. 2021; Yurkov et al. 2022). As reported by Yurkov et al. 2022, members of both genera *Naganishia* and *Cystobasidium* (*N. albida* and *C. minutum*) were nearly the only yeasts isolated from hot and dry sandy soil of the Namib desert. *N. albida* is well-adapted to dry conditions due to a thick polysaccharides capsules, which might be responsible for its remarkable ability to survive in air-dried autoclaved loamy sand (Vishniac 1985). Highly desiccation tolerance has also been reported in *N. vishniacii,* showing almost 100% survival after 27 days at a relative humidity of < 1% (Connell et al. 2014). Another species of the genus, *N. friedmannii* tested for desiccation (along with stratospheric low pressure and temperature and UV exposure) during stratospheric balloon flights, recorded larger numbers of viable cells than bacterial spores. Presence of the genetic traits associated with desiccation and oligotrophy were present in *N. kalamii* and *C. onofrii*, promoting their presence to the stringent conditions found in cleanrooms. Our genomic analysis revealed that along with genes related to desiccation tolerance, genes related to UV resistance are present in both *Naganishia* and *Cystobasidium* members. However, the only strain exhibiting all gene ontology terms involved in UV resistance is *N. vishniacii* ANT03-052, firstly isolated from the McMurdo Dry Valleys (Antarctica), a terrestrial Mars analogue. We may assume that local physical–chemical parameters of the environment might be one of the evolutionary drivers that induce the stabilization of the genes required to survive in the extreme conditions (Aureli et al. 2023).

UV-C resistance may be a critical factor for microbial survival during interplanetary transport. Most work correlates increased UV-C resistance with microbial pigmentation (Kreusch and Duarte 2021). Few studies recognized the existence of non-pigmented yeasts with similar resistance (Moliné et al. 2010; Schmidt et al. 2017; Pulschen et al. 2018; Kreusch and Duarte 2021). Our results showed UV-C survival of the non-pigmented *N. kalamii* and the pigmented pink-colored *C. onofrii* at up to 3000 J/m^2^ UV-C irradiation. Multiple species from the non-pigmented *Naganishia* species are highly resistant organisms to UV radiation (Pulschen et al. 2015; Schmidt et al. 2017) and our results are in line with this finding. Indeed, we found that the polar yeast *N. onofrii* is more resistant to UV-C radiation compared to the pigmented *C. onofrii,* which is capable of synthesizing carotenoids with UV shielding properties (Moliné et al. 2010). Furthermore, cold-adapted yeasts, such *N. onofrii* are characterized by a thick cell wall and the ability to produce an extracellular polysaccharide capsule surrounding their cells (Turchetti et al. 2015), providing robustness to the organism when exposed to stress conditions. This adaptation is typically found in extremophiles. Indeed, it is already demonstrated that deinococcal polysaccharide capsule (DeinoPol) has antioxidant properties involved in resistance to severe environmental stresses, including UV radiation and hydroxyl radicals (Lin et al. 2020). During atmospheric transportation, microorganisms must cope up with not only with intense UV, but also low temperatures and dehydration (Selbmann et al. 2005, 2013).

The serine proteases of *N. kalamii* also include some hydrolases which were reported to degrade lipids (Leger et al. 1997; Muszewska et al. 2017). Protein tyrosine and serine/threonine kinase (PF07714), WD domain, G-beta repeat (PF00400), Ras family (PF00071), Cytochrome P450 (PF00067), Ankyrin repeats (PF12796) and ABC transporter (PF00005) are the most abundant Pfam terms in all the *Naganishia* species and are usually associated with survival of multiple dessication-rehydration cycles (Gao et al. 2017). Heat shock protein 70 and other Pfam terms for predicted proteins involved in the dehydration stress-response were also present in all the *Naganishia* species analyzed. However, only the psychrophilic *N. vishniacii* ANT03-052 had genes involved in raffinose synthesis (PF05691). Raffinose accumulation is one of the adaptive strategies involved against cold shock (Imanishi et al. 1998; Egert et al. 2013; Yang et al. 2022).

By comparing the gene conservation and functional annotation in the different species analyzed, we identified that both *Naganishia* and *Cystobasidium* species present a consistent number of genes related to a saprotrophic lifestyle of decaying organic and plant materials (PL36; GH5; CE10; GH128; AA1). Moreover, serine proteases S33 and S09X found in *N. kalamii,* are essential hydrolytic enzymes with several functions such as protein maturation, signal peptide cleavage and transduction, and intracellular protein turnover. Although both are typically understudied in yeast, they have been suggested as a marker for saprotrophy in fungi as they are theorized to be less expressed in fungi with a pathogenic or animal-associated lifestyle (Muszewska et al. 2017). Furthermore, it has also been suggested that the relatively high abundance of the aminopeptidases M01 and the serine proteases S12, which are both detected in abundance in the genome of *N. kalamii,* may be an indicator of saprotrophy (Muszewska et al. 2017). The same trend has been detected in *C. onofrii.* Furthermore, genes encoding a wide range of glycosyl hydrolases that have the capacity to degrade the major plant cell wall polymers (eg. AA1 for lignin and GH5 for cellulose breakdown) were found in both *N. kalamii* and *C. onofrii*. Acetyl esterases, found in *C. onofrii,* are an important component of the enzymatic machinery for fungal degradation of plant biomass and break down xylan-derived oligosaccharides and on galactoglucomannan (Venegas et al. 2022). In particular, the Acetyl esterases subfamily members are preproenzymes, acting in the lysosomal/endosomal system and may be involved in the external digestion of proteins in saprophytic organisms (Chan et al. 2014). The synthesis of extracellular enzymes which degrade organic matter and release nutrients is crucial for the survival of saprotrophs. However, by comparing the gene conservation and functional annotation of the two yeasts described here, we observed a loss in gene family (PFAM terms and GO-term counts) in both species, which is usually associated with a host/substrate shift from plants to animals. It is worth mentioning that the loss is more remarkable in *N. kalamii* (Muszewska et al. 2017).

## CONCLUSIONS

This study underscores the extreme, oligotrophic conditions of meticulously maintained NASA cleanrooms, which could potentially house various microbes, including significant yeast species. Traditional yeast identification methods are deemed inadequate, highlighting the need for more precise techniques like MLSA and WGS, particularly for complex genera like *Naganishia*. The study also warns about these UV-C resistant yeasts, including high-altitude, cold-adapted *N. onofrii* and two other NASA cleanroom species, to potentially contaminate extraterrestrial environments, thereby posing risks to life-detection missions. The presence of these cold-adapted yeasts in SAF bears implications for future missions to icy moons. Genomic analysis further reveals the potential for survival in harsh conditions, pointing to genes linked with desiccation tolerance and UV resistance.

### Supplementary Information

**Additional file 1**: **Table S1**. List of accession numbers of genes used for the generation of phylogenetic trees for the strain *N. kalamii*. **Table S2**. List of accession numbers of genes used for the generation of phylogenetic trees for the strain *C. onofrii*. **Table S3**. Physiological characteristics of *Naganishia kalamii*, and closely related species. **Table S4**. Genomic features of the 7 Naganishia species considered in the following study. **Table S5**. Physiological characteristics of *Cystobasidium onofrii*, and closely related species. **Table S6**. The identity reports on the percentage of base pairs of the seven marker loci sequence between *C. onofrii* and all available *Cystobasidium strains*. **Table S7**. Genomic features of the 5 Cystobasidium species considered in the following study. **Table S8**. Data summary table from multiple variables.

**Additional file 2**: **Fig. S1**. Maximum likelihood phylogenetic tree, based on concatenated ITS and LSU gene sequences of *N. kalamii* and related *Naganishia* species, class *Tremellomycetes*, order *Filobasidiales*. The tree was rooted with *G. gastricus* type strains. Bootstrap percentages from 1000 replications are shown on the branches (values below 50.00 are not shown). GenBank accession numbers of the sequences are listed in Additional file 1: Table S1. **Fig. S2**. Pairwise sequence genetic distance matrix based on LSU (below the diagonal) and ITS (above the diagonal) gene sequences of *N. kalamii* and related *Naganishia* species (albida clade highlighted). The function dist.dna in the ape: Analyses of Phylogenetics and Evolution package (R software) with the Kimura 2-parameter substitution model was used for the generation of the matrix. **Fig. S3**. SSU phylogenetic tree of *N. kalamiii* and related taxa of *N. albida *clade (shown only in part), class *Tremellomycetes*, order *Filobasidiales*. The tree was rooted with *G. gastricus* type strains. Bootstrap percentages from 1000 replications are shown on the branches (values below 50.00 are not shown). GenBank accession numbers of the sequences are listed in Additional file 1: Table S1. **Fig. S4**. CYTB phylogenetic tree of *N. kalamiii* and related taxa of *N. albida* clade (shown only in part), class *Tremellomycetes*, order *Filobasidiales*. The tree was rooted with G. gastricus type strains. Bootstrap percentages from 1000 replications are shown on the branches (values below 50.00 are not shown). GenBank accession numbers of the sequences are listed in Additonal file 1: Table S1. **Fig. S5**. TEF1 phylogenetic tree of *N. kalamiii* and related taxa of *N. albida* clade (shown only in part), class *Tremellomycetes*, order *Filobasidiales*. The tree was rooted with *G. gastricus* type strains. Bootstrap percentages from 1000 replications are shown on the branches (values below 50.00 are not shown). GenBank accession numbers of the sequences are listed in Additional file 1: Table S1. **Fig. S6**. RPB1 phylogenetic tree of *N. kalamiii* and related taxa of *N. albida* clade (shown only in part), class *Tremellomycetes*, order *Filobasidiales*. The tree was rooted with *G. gastricus* type strains. Bootstrap percentages from 1000 replications are shown on the branches (values below 50.00 are not shown). GenBank accession numbers of the sequences are listed in Additional file 1: Table S1. **Fig. S7**. RPB2 phylogenetic tree of *N. kalamiii* and related taxa of *N. albida* clade (shown only in part), class *Tremellomycetes*, order *Filobasidiales*. The tree was rooted with *G. gastricus* type strains. Bootstrap percentages from 1000 replications are shown on the branches (values below 50.00 are not shown). GenBank accession numbers of the sequences are listed in Additional file 1: Table S1. **Fig. S8**. Comparative genomic analysis. a) Phylogenetic tree showing gain and loss Interpro domain among 7 *Naganishia* genomes and b) the relative predicted secreted proteins. **Fig. S9**. Comparative genomic analysis. a) Phylogenetic tree showing gain and loss of Interpro domains among 7 *Naganishia* genomes and b) the relative distribution of the predicted protease (MEROPS) groups and, the most present c) families and subfamilies. **Fig. S10**. Comparative genomic analysis. a) Phylogenetic tree showing gain and loss of Interpro domains among 7 *Naganishia* genomes and b) the relative distribution of the predicted proteins terms filtered by dehydration, desiccation, rehydration and UV resistance into Pfam and Interpro database. **Fig. S11**. Comparative genomic analysis. a) Phylogenetic tree showing gain and loss of Interpro domains among 7 *Naganishia* genomes and b) the relative distribution of the fungal-specific transcriptional factors. Factors with a standard deviation >1 was plotted in a heat map. **Fig. S12**. ITS phylogenetic tree of C. onofrii. The tree was rooted with *R. mucilaginosa* and *N. sebacea* type strains. Bootstrap percentages from 1000 replications are shown on the branches (values below 50.00 are not shown). GenBank accession numbers of the sequences are listed in Additional file 1: Table S2. **Fig. S13**. LSU phylogenetic tree of *C. onofrii*. The tree was rooted with *R. mucilaginosa* and *N. sebacea* type strains. Bootstrap percentages from 1000 replications are shown on the branches (values below 50.00 are not shown). GenBank accession numbers of the sequences are indicated after strain numbers and listed in Additional file 1: Table S2. **Fig. S14**. SSU phylogenetic tree of *C. onofrii*. The tree was rooted with *R. mucilaginosa* and *N. sebacea* type strains. Bootstrap percentages from 1000 replications are shown on the branches (values below 50.00 are not shown). GenBank accession numbers of the sequences are listed in Additional file 1: Table S2. **Fig. S15**. CYTB phylogenetic tree of *C. onofrii*. The tree was rooted with *R. mucilaginosa* and *N. sebacea* type strains. Bootstrap percentages from 1000 replications are shown on the branches (values below 50.00 are not shown). GenBank accession numbers of the sequences are listed in Additional file 1: Table S2. **Fig. S16**. TEF1 phylogenetic tree of *C. onofrii*. The tree was rooted with *R. mucilaginosa* and *N. sebacea* type strains. Bootstrap percentages from 1000 replications are shown on the branches (values below 50.00 are not shown). GenBank accession numbers of the sequences are listed in Additional file 1: Table S2. **Fig. S17**. RPB1 phylogenetic tree of *C. onofrii*. The tree was rooted with *R. mucilaginosa* and *N. sebacea* type strains. Bootstrap percentages from 1000 replications are shown on the branches (values below 50.00 are not shown). GenBank accession numbers of the sequences are listed in Additional file 1: Table S2. **Fig. S18**. RPB2 phylogenetic tree of *C. onofrii*. The tree was rooted with *R. mucilaginosa* and *N. sebacea* type strains. Bootstrap percentages from 1000 replications are shown on the branches (values below 50.00 are not shown). GenBank accession numbers of the sequences are listed in Additional file 1: Table S2. **Fig. S19**. Comparative genomic analysis. a) Phylogenetic tree showing gain and loss Interpro domain among 5 Cystobasidium genomes and b) the relative predicted secreted proteins. **Fig. S20**. Comparative genomic analysis. a) Phylogenetic tree showing gain and loss of Interpro domains among 5 Cystobasidium genomes and b) the relative distribution of the predicted protease (MEROPS) groups and, the most present c) families and subfamilies. **Fig. S21**. Comparative genomic analysis. a) Phylogenetic tree showing gain and loss of Interpro domains among 5 Cystobasidium genomes and b) the relative distribution of the predicted proteins terms filtered by dehydration, desiccation, rehydration and UV resistance into Pfam and Interpro database. **Fig. S22**. Comparative genomic analysis. a) Phylogenetic tree showing gain and loss of Interpro domains among 5 Cystobasidium genomes and b) the relative distribution of the fungal-specific transcriptional factors. Factors with a standard deviation > 1 was plotted in a heat map. **Fig. S23**. Survivability of various yeasts exposed to UV-C radiation (doses: 0, 500, 1000, 2000 and 3000 J/m^2^. UV-C survival rates calculated using formula: N/N0, where N is the number of cells surviving at any given dose and N0 is the number of cultivable cells after desiccation and PVA treatment considered as baseline (0). The ratio of survival rates from three different species are plotted. The gray box shows the effect of desiccation and PVA treatment. The experiments were repeated twice. **Fig. S24**. Cultivation test performed in colonies exposed to UV-C radiation (doses: 0, 500, 1000, 2000 and 3000 J/m^−2^). Bar-plot showing number of viable cells after UV-C treatment. Significant differences were calculated by t-test with ∗*p* < 0.05; ∗∗*p* < 0.001; ∗∗∗*p* < 0.0001 and ∗∗∗∗*p* < 0.00001 (see Additional file 1: Table S7).

## Data Availability

The draft genomes JAKLLX000000000 and JAKLNA000000000 and raw data (SAMN25226776 and SAMN25226807), have been deposited in GenBank under the BioProject accession number PRJNA800051. C.E.M. thanks the Scientific Computing Unit (SCU) at WCM, the WorldQuant and GI Research Foundation, NASA (80NSSC22K0254, 80NSSC23K0832, the National Institutes of Health (R01MH117406), and the LLS (MCL7001-18, LLS 9238-16, 7029-23).

## Abbreviations

A01A: Aspartic peptidases subfamily 1A
AA: Auxiliary activities
AA1: Auxiliary activities family 1
AAFTF: Automatic assembly for the fungi
ABC: ATP-binding cassette transporters
ANI: Average nucleotide identity
BGC: Biosynthetic gene cluster
Blastn: Basic local alignment search tool
BUSCO: Benchmarking Universal single-copy orthologs
Bzip TF1: Basic leucine zipper domain transcription factor 1
CAFE5: Computational analysis of gene family evolution v.5
CAZymes: Carbohydrate active enzymes
CBM: Carbohydrate binding module
CBS: CBS-KNAW culture collection, Westerdijk Fungal Biodiversity Institute, Utrecht, The Netherlands.
CE: Carboxyl esterases
CE10: Carboxyl esterases family 10
CE16: Carboxyl esterases family 16
CFUs: Colony forming units
*CYTB*: Cytochrome B gene
DBVPG: Collection of yeasts, Dipartimento di Biologia Vegetale, Perugia, Italy
DNA: Deoxyribonucleic acid
DSM: DSMZ-Deutsche Sammlung von Mikroorganismen und Zellkulturen GmbH, Braunschweig, Germany.
EMBL: European molecular biology laboratory
ESA: European space agency
GH: Glycoside hydrolase
GH128: Glycoside hydrolase family 128
GH5: Glycoside hydrolase family 5
GO: Gene ontology
GT: Glycosyl transferase
HEPA: High efficiency particulate air
HQ: High quality
ISS: International space station
ITS: Internal transcribed spacer
JCM: Japan collection of microorganisms
JPL-SAF: Jet propulsion laboratory spacecraft assembly facility
KSC-PHSF: Kennedy space center payload hazardous and servicing facility
LEV: Low elution volume
LSU: Large-subunit ribosomal DNA
M01: Metallo peptidase family 1
M03A: Metallo peptidase subfamily 3A
M16A: Metallo peptidase subfamily 16A
M38: Metallo peptidase family 38
MLSA: Multi-locus sequence analysis
NASA: National aeronautics and space administration
NCBI: National center for biotechnology information
NRPS-Like: Non-ribosomal peptide synthase-like
NRRL: Northern regional research laboratory
PBS: Phosphate-buffered saline
PCR: Polymerase chain reaction
PDA: Potato dextrose agar
PL: Polysaccharide lyase
PL36: Polysaccharide lyase family 36
PP: Planetary protection
PVA: Polyvinyl alcohol
QUAST: Quality assessment tool for genome assemblies
RAD2: DNA repair protein RAD2
RNA: Ribonucleic acid
*RPB1*: RNA polymerase II large subunit I gene
*RPB2*: RNA polymerase II large subunit II gene
S09X: Serine peptidases subfamily 9X
S12: Serine peptidases family 12
S33: Serine peptidases family 33
SAF: Spacecraft assembly facility
SSU: Small-subunit ribosomal DNA
*TEF1*: Translation elongation factor EF-1 Alpha gene
USDA: United States department of agriculture
UV-C: Ultraviolet-C
WD domain: Tryptophan-aspartic acid domain
WGS: Whole genome sequencing
XPG: Xeroderma pigmentosum complementation group G
