# Supplementary material for: Genomic characterization and radiation tolerance of Naganishia kalamii sp. nov. and Cystobasidium onofrii sp. nov. from Mars 2020 mission assembly facilities
Source: IMA Fungus. 2023 Aug 11;14:15. doi: 10.1186/s43008-023-00119-4 (PMC10422843; doi:10.1186/s43008-023-00119-4)
Supplement: Supplementary file 1 — Additional file 1: Table S1. List of accession numbers of genes used for the generation of phylogenetic trees for the strain N. kalamii. Table S2. List of accession numbers of genes used for the generation of phylogenetic trees for the strain C. onofrii. Table S3. Physiological characteristics of Naganishia kalamii, and closely related species. Table S4. Genomic features of the 7 Naganishia species considered in the following study. Table S5. Physiological characteristics of Cystobasidium onofrii, and closely related species. Table S6. The identity reports on the percentage of base pairs of the seven marker loci sequence between C. onofrii and all available Cystobasidium strains. Table S7. Genomic features of the 5 Cystobasidium species considered in the following study. Table S8. Data summary table from multiple variables. [file 43008_2023_119_MOESM1_ESM.pdf]

Supplemental Table ST1. List of accession number of genes used for the generation of phylogenetic trees for the strain *N. kalamii*

Strain number	Taxon name	ITS	LSU	SSU	*RPB1*	*RPB2*	*TEF1*	*CYTB*
CBS2288	*Goffeauzyma gastricus*	AF145323.1	AF137600.1	AB032633.1	KF036373.1	KF036785.1	KF037057.1	AB040652.1
CBS8351	*Naganishia adeliensis*	AF145328	AF137603	KF036610	KF036335	KF036747	KF037018	KF423159
CBS142	*Naganishia albida* var. *albidus*	AF145321	AF075474	AB032616	/	KF036751	KF037022	KF423163
CBS1926	*Naganishia albida* var. *kuetzingii*	AF145327	AF137602	AB032639	KF036340	KF036753	KF037024	KF423165
CBS5810	*Naganishia albida* var. *ovalis*	AF145329	AF137605	KF036614	/	KF036754	KF037025	KF423166
CBS7711	*Naganishia albidosimilis*	AF145325.1	AF137601.1	KF036612.1	KF036338.1	KF036750.1	KF037021.1	KF423162.1
CBS7687	*aganishia antarctica* var. *antarctic*	AF145326	AF075488	AB032620	KF036345	/	KF037030	KF423169
CBS7689	*ganishia antarctica* var. *circumpola*	NR152963	KM079157	KF036618	KF036346	KF036759	KF037031	KF423170
CBS6294	*Naganishia bhutanensis*	AF145317.1	AF137599.1	NG063459.1	KF036352.1	KF036765.1	KF037037.1	KF423176.1
BRIP 28244	*Naganishia brisbanensis*	MZ766444.1	/	/	/	/	/	/
CBS 10505	*Naganishia cerealis*	FJ473371.1	FJ473376.1	KF036624.1	KF036356.1	/	KF037041.1	KF423180.1
CBS160	*Naganishia diffluens*	AF145330.1	AF075502.1	KF036630.1	KF036363.1	KF036775.1	KF037048.1	KF423187.1
CBS 10D4	*Naganishia floricola*	MK942576.1	MK942558	/	/	/	/	/
CBS7160	*Naganishia friedmannii*	AF145322.1	AF075478.1	AB032630.1	KF036371.1	KF036783.1	KF037055.1	KF423194.1
CBS1975	*Naganishia globosa*	AF444372.1	AF181540	KF036651.1	KF036400.1	KF036814.1	KF037085.1	KF423222.1
RNF 072	*Naganishia indica*	/	MF929073	/	/	/	/	/
CBS 968	*Naganishia liquefaciens*	AF444345	AF181515	KF036638	KF036381	KF036794	KF037066	KF423203
DBVPG 5693	*Naganishia nivalis*	MK070337	KC433768	/	/	/	/	/
DBVPG 5303	*Naganishia onofrii*	KC455900.1	KC433831.1	/	/	/	/	/
QCC-Y17/17	*Naganishia qatarensis*	MG852088.1	KY744128	/	/	/	/	/
CBS10160	*Naganishia randhawae*	KY104335	KY108617	KF036650.1	/	/	/	/
J11	*Naganishia randhawae*	AJ876528.1	AJ876599.1	/	/	/	/	/
CBS 8683	*Naganishia uzbekistanensis*	AF444339.1	AF181508.1	KF036660.1	KF036412.1	KF036826.1	KF037096.1	KF423232.1
DBVPG5325	*Naganishia vaughanmartiniae*	KC455904	KC433840	/	/	/	/	/
CBS 7110	*Naganishia vishniacii*	AF145320.1	AF075473.1	AB032650.1	KF036414.1		KF037098.1	KF423234.1
CBS 6294	*Naganishia bhutanensis*	AF145317	AF137599	AB032623	KF036352	KF036765	KF037037	KF423176
IF6SW-B1	*Naganishia tulchinskyi*	KY218715.1	*	*	*	*	*	*
FJI-L2-BK-P3	*Naganishia kalamii*	*	*	*	*	*	*	*

*** gene sequences extracted from WGS**
/ Not Available

Supplemental Table ST2. List of accession number of genes used for the generation of phylogenetic trees for the strain *C. onofrii*

Strain number	Taxon name	ITS	LSU	SSU	*RPB1*	*RPB2*	*TEF1*	*CYTB*
JCM 10901	*Cystobasidium benthicum*	AB026001	AB026001	AB126647	KJ708081	KJ708214	KJ707842	KJ707691
JCM 10899	*Cystobasidium calyptogenae*	AB025996	AB025996	AB126648	KJ708075.1	KJ708218.1	KJ707840.1	KJ707690.1
JCM 10953	*Cystobasidium laryngis*	AB078500	AB078500	AB126649	KJ708055.1	KJ708240.1	KJ707824.1	KJ707619.1
JCM 5951	*Cystobasidium lysinophilum*	AB078501	AB078501	AB126650	KJ708074.1	KJ708243.1	KJ707845	KJ707721.1
CBS 319	*Cystobasidium minutum*	AF190011	AF189945	D45367	KJ708059.1	KJ708246.1	KJ707825.1	KJ707562.1
JCM 3780	*Cystobasidium pallidum*	AB078492	AF189962	AB126651	KJ708056.1	KJ708253.1	KJ707826	KJ707621.1
CBS 9130	*Cystobasidium pinicola*	AF444292	AF444293	AB126652	KJ708057.1	KJ708257.1	KJ707827.1	KJ707579.1
CBS 15509	*Cystobasidium raffinophilum*	NR_174780.1	MK050389.1	MK050389.1	MK849191.1	MK849329.1	MK849058.1	MK848927.1
JCM 10954	*Cystobasidium slooffiae*	AF444627	AF444722	AB126653	KJ708266.1	KJ708058.1	KJ707828.1	KJ707629.1
CBS 15650	*Cystobasidium terricola*	NR_174781.1	MK050391.1	MK050390.1	MK849330.1	MK849331.1	MK849059.1	MK848928.1
JCM 31527	*Cystobasidium ongulense*	LC155915.1	LC203680.1	LC158351.1	/	/	LC158353.1	LC158355.1
JCM 31526	*Cystobasidium tubakii*	LC155914.1	LC155913.1	LC158350.1	/	/	LC158352.1	LC158354.1
KM 1106	*Cystobasidium oligophagum*	MN244409.1	ON644561.1	NG063083.1	/	/	/	/
DBVPG 10041	*Cystobasidium alpinum*	NR_159815.1	KC433879.1	/	/	/	/	/
CBS 8253	*Erythrobasidium hasegawianum*	AF444522	AF189899	D12803	KF706506	KF706534	KJ707776	KJ707563
JCM 8115	*R. mucilaginosa*	AF444541	AF070432	AB021668	/	KJ708247	KJ707861	KJ707731
CBS 8477	*Naohidea sebacea*	DQ911616	DQ831020	KP216515	KF706508	KF706535	KF706487	KJ707654
PYCC 6649	*Cystobasidium fimetarium*	LM644067.1	AY512843	AY124479	/	/	LM644071.1	/
CBS 11769	*Cystobasidium psychroaquaticum*	NR_171727.1	KY107444.1	KY103148.1	/	/	LM644068.1	/
MUCL 53589	*Cystobasidium ritchiei*	NR_154854.1	KY107445.1	LM644066.1	/	/	LM644069.1	/
FJI-L9-BK-P1	*Cystobasidium onofrii*	*	*	*	*	*	*	*

*** gene sequences extracted from WGS**
/ Not Available

Supplementary Table S3. Physiological characteristics of *Naganishia kalamii* , and closely related species.

	N. kalamii	*N. albida*	*N.* var. *kuetzingi*	*N.* var. *ovalis*	*N. nivalis*	*N. adeliensis*	*N. tulchinskyi*	*N. vaughanmartiniae*	*N. onofrii*	*N. saitoi*	*N. friedmannii*	*N. qatarensis*	*N. cerealis*	*N. randhawae*	*N. globosa*
**Assimilation of carbon compounds**															
D-glucose	+	+	+	+	+	+	+	+	+	+	+	-	NT	NT	NT
D-galactose	w	-	-	-	+	w	-	+	+	-/w	-	+	+	+	w
D-xylose	+	+	+	+	+	+	+/w	+	+	+	+	+	+	+	+
D-glucosamine	-	-	-	-	+	-	-	-	-	NT	NT	-	NT	NT	NT
D-ribose	-	w	w/ -	d	+	-	+	+	v	v	-	+	v	-	-
L-sorbose	-	-	-	-	-	-	+	v	v	-	-	+	+	-	-
L-Arabinose	+	+	+	+	w	+	+	+	+	+	+	+	w	w	-
D-Arabinose	-	-	-	d	d	-	+	+	+	-	-	+	+	w	-
L-Rhamnose	-	-	-	w/ -	-	w	-	+	+	+	-	+	+	+	+
Sucrose	+	+	+	+	d	+	+	+	+	+	v	-	NT	NT	NT
Maltose	+	+	-	+	+	+	+	+	+	+	+	-	NT	NT	NT
a,a-Trehalose	-	+	+	+	+	+	+	+	+	+	+	+	+	+	+
Methyl a-glucoside	w	+	-	-	+	-	-	+	+	+	v	-	NT	NT	NT
Cellobiose	+	+	+	+	+	+	-	+	+	+	+	-	NT	NT	NT
Salicin	-	+	+	+	+	+	-	+	+	+	+	-	NT	NT	NT
Melibiose	-	-	-	-	+	-	-	-	-	-	-	+	-	-	-
Lactose	w	w/d	-	+	+	w	NT	NT	NT	NT	NT	NT	NT	NT	NT
Raffinose	+	+	+	+	+	+	-	-/w	+	-/w	-	+	w	+	w
Melezitose	w	+	-	+	+	+	-	+	+	+	+	-	NT	NT	NT
D-Trehalose	+	+	NT	NT	+	NT	+	+	+	+	+	-	NT	NT	NT
D-Xylose	+	+	NT	NT	+	NT	+	+	+	+	+	-	NT	NT	NT
D-Mannitol	+	+	NT	NT	+	NT	+	NT	NT	NT	-	+	+	+	+
Inulin	-	-	-	-	NT	-	-	NT	NT	NT	NT	+	W	-	+
Starch	+	+	-	-	+	+									
Glycerol	-	-	-	w/d	+	-	-	v	+	-/d	-	+	-	-	-
erythritol	-	-	-	-	+	-	-	-	-	-	-	w	-	-	-
Ribitol	-	w/ -	w/-	w/d	+	-									
Xylitol	-	+	w	d	+	+	-	+	+	-	-	+	+	+	-
D-Glucitol	+	+	+	+	+	+									
D-Mannitol	+	+	+	+	+	+	+	+	+	+	-	+	+	+	+
Galactitol	+	-	-	-	+	w/-	NT	NT	NT	NT	NT	NT	NT	NT	NT
Myo-inositol	+	+	+	+	+	+	-	v	+	+	-	+	+	+	+
5-Keto-D-Gluconate	-	NT	NT	NT	NT	NT	NT	NT	NT	NT	NT	NT	NT	NT	NT
D-Gluconate	+	+	w	d	-	-	NT	NT	NT	NT	NT	NT	NT	NT	NT
D-Glucuronate	+	+	+	+	-	+	NT	NT	NT	NT	NT	NT	NT	NT	NT
D-Galacturonate	-	+	-	-	-	-	NT	NT	NT	NT	NT	NT	NT	NT	NT
DL-Lactate	-	+	+	w	-	-	NT	NT	NT	NT	NT	NT	NT	NT	NT
Succinate	w	+	+	+	-	-	NT	NT	NT	NT	NT	NT	NT	NT	NT
Citrate	-	+	w	w	-	+	NT	NT	NT	NT	NT	NT	NT	NT	NT
Methanol	-	-	-	-	+	-	NT	NT	NT	NT	NT	NT	NT	NT	NT
Ethanol	+	+	+	w	+	+	NT	NT	NT	NT	NT	NT	NT	NT	NT
D-glucarate	+	+	+	+	NT	+	NT	NT	NT	NT	NT	NT	NT	NT	NT
L-Malic acid	+	+	+	NT	-	+	-	-	-	+	v	NT	NT	NT	NT
L-Tartaric acid	-	-	+	-	NT	+	NT	NT	NT	NT	NT	NT	NT	NT	NT
D-Tartaric acid	-	NT	NT	NT	NT	NT	NT	NT	NT	NT	NT	NT	NT	NT	NT
**Assimilation of nitrogen compounds**															
potassium Nitrate	+	+	+	+	+	+	+	NT	NT	NT	NT	NT	NT	NT	NT
sodium Nitrite	+	+	+	+	+	+	+	NT	NT	NT	NT	NT	NT	NT	NT
Ethylamine	-	-	-	-	+	-	-	+	+	NT	NT	-	NT	NT	NT
L-Lysine	+	+	+	+	+	-	+	NT	NT	NT	NT	NT	NT	NT	NT
Cadaverine	-	+	+	+	w	+	+	+	+	+	+	+	+	+	+
Creatine	-	-	-	-	-	-	+	+	+	NT	NT	NT	NT	NT	NT
Creatinine	-	-	-	-	-	-	+	NT	NT	NT	NT	NT	NT	NT	NT
Glucosamine	-	-	-	-	-	-	+/w	NT	NT	NT	NT	NT	NT	NT	NT
Imidazole	-	-	-	-	-	-	+/w	NT	NT	NT	NT	NT	NT	NT	NT
D-Tryptophan	-	w	-	-	-	-	+/w	NT	NT	NT	NT	NT	NT	NT	NT
**Other tests:**															
Growth with 10% NaCl	w	-	-	-	-	-	NT	NT	NT	NT	NT	NT	NT	NT	NT
Growth with 8% NaCl	+	NT	NT	NT	NT	NT	NT	NT	NT	NT	NT	NT	NT	NT	NT
Growth with 5% NaCl	+	NT	NT	NT	NT	NT	NT	NT	NT	NT	NT	NT	NT	NT	NT
Growth with 60% glucose	-	-	w	-	-	-	NT	NT	NT	NT	NT	NT	NT	NT	NT
Growth with 50% glucose	-	NT	NT	NT	NT	NT	NT	NT	NT	NT	NT	NT	NT	NT	NT
Growth at 4 °C	+	+	NT	NT	+	NT	+	+	+	NT	+	NT	NT	NT	NT
Growth at 25 °C	+	+	NT	NT	+	NT	+	+	+	+	-	+	+	+	+
Growth at 30 °C	+	-	NT	NT	NT	NT	+	-	-	+	-	+	+	+	+
Growth at 35 °C	+	-	NT	NT	NT	NT	-	-	-	-	-	+	-	-	-

Data from Sugita et al. 2001. Turchetti et al. 2015. Data obtained from Fonseca et al. 2011. Data obtained from Fotedar et al. 2018. Data obtained from Passoth et al. 2009. Data obtained from Khan et al. 2010. Data obtained from Parker et al. (2022) Genomic Characterization of the Titan-like Cell Producing Naganishia tulchinskyi, the First Novel Eukaryote Isolated from the International Space Station (2022) Abbreviation: “+” positive; “-” negative; “NT” not tested; “w” weak reaction; “v” variable reaction; “d” delayed.

Supplementary Table ST4. Genomic features of the *Naganishia* species

**Taxon name**	***Naganishia kalamii***	***Naganishia* sp.**	***Naganishia vishniacii***	***Naganishia* sp.**	***Naganishia* sp.**	***Naganishia* sp.**	***Naganishia tulchinskyi***
**Isolate**	FJI-L2-BK-P3	N6	ANT03-052	eABCC1	NRRL-Y-1402	JCM_2334	IF6SW-B1
**GeneBank ID**	GCA_022813745.1	GCA_013423385.1	GCA_015708705.1	GCA_013461525.1	GCA_001444555.1	GCA_001599735.1	GCA_012922605.1
**locus_tag**	OHC07	N6N66	ANT03	ABCC1	NRRL1	JCM233	L6Q86
**Assembly Size (bp)**	27,861,060	19,480,866	19,679,091	20,269,202	24,654,795	20,695,791	19,422,953
**Largest Scaffold (bp)**	1,000,601	1,630,525	1,840,649	793,440	477,187	1,576,301	1,169,194
**Average Scaffold (bp)**	58,287	463,830	517,871	53,061	40,089	283,504	151,742
**Num Scaffolds**	478	42	38	382	615	73	128
**Scaffold N50 (bp)**	190,774	1,033,977	1,080,776	191,697	115,800	975,819	506,783
**Percent GC (%)**	53.93	53.32	52.86	51.71	52.70	53.82	53.35
**Num Genes**	8,085	6,015	6,376	6,252	7,244	6,413	6,222
**Num Proteins**	7,902	5,844	6,216	6,083	7,067	6,241	6,053
**Num tRNA**	183	171	160	169	177	172	169
**Unique Proteins**	1,495	483	579	704	1,056	499	359
**Prots atleast 1 ortholog**	5,946	5,231	5,474	5,185	5,726	5,548	5,537
**Single-copy orthologs**	3,423	3,423	3,423	3,423	3,423	3,423	3,423

Supplementary Table ST5. Physiological characteristics of *C. onofrii* , and closely related species.

	C. onofrii	*C. halotoleranse*	*C. sloofiae*	*C. minutum*	*C. fimetarium*	*C. psychroaquaticum*	*C. ritchiei*	*C. benthicum*	*C. calyptogenae*	*C. laryngis*	*C. lysinophilum*	*C. oligophagum*	*C. ongulense*	*C. tubakii*	*c. pallidum*	*C. pinicola*	*C. alpinum*
**Assimilation of carbon compounds**																	
D-Glucose	+	+	+	+	+	+	+	+	+	+	NT	NT	+	+	+	NT	+
D-Galactose	w	+	–	–/w/S	–	–	–	+	+	–/D	NT	NT	–	w	–	+	w
L-Sorbose	w	+/D	+/(S*)	+(+/S*)	–	V	w	w	–	V(–/w*)	–	–	–	–	+(w/S*)	–	–
D-Glucosamine	-	–	–	–	–	–	w	–	–	–	–	–	–	–	–	–	+
D-Ribose	-	–	S	+/S	+	V	+	+	+	–	NT	NT	–	–	+	w	–
D-Xylose	+	+	+	+	+	+	+	+	+	+	NT	NT	+	+	+	NT	+
L-Arabinose	+	+	+/S	+	+	+	+	+	+	+	NT	NT	+	–	–	NT	+
D-Arabinose	-	–	+/(+/s*)	+	+	+	+	+	+	+	+	–	+	–	–	+	w
L-Rhamnose	-	–	–	–	–	–	–	+	w/(–*)	–	NT	NT	–	–	–	w	–
Sucrose	+	+	+	+	V	+	+	+	–	+	+	+	+	+	–	–	V
Maltose	-	–	–	–	–	–	–	+	+	–	+	+	–	–	–	+	–
Trehalose	+	+	V	+	+	+	w	+	+	+	NT	NT	+	w	+	NT	+
Methyl a-D-glucoside	-	–	–	–	–	–	+	w	w/(–*)	NT	NT	–	–	–	–	w	
Cellobiose	+	+	+	+	+	V	+	w	+	+	+	–	–	w	–	+	+
Salicin	-	–	V	+/(S*)	+	+	+	–	+	+	–	–	+	+	–	+	+
Melibiose	-	–	–	–	–	–	–	+	+	–	NT	NT	–	–	–	NT	w
Gentiobiose	+	+	+(w/S*)	+	+/D	–	–	+/(w*)	+	–	+	–	–	–	–	+	w
Lactose	+	NT	NT	NT	NT	-	-	NT	NT	NT	NT	NT	NT	NT	NT	NT	NT
Raffinose	-	–	–	–	–	–	–	+	+	–	+	–	–	–	–	+	w
Melezitose	+	+	+	+	–	+	+	+	+	+	+	+	+	+	–	+	w
Inulin	w	–	–	–	–	–	–	+	+	–	NT	NT	–	–	–	NT	NT
Soluble starch	w	+	–	–	–	–	–	+	w	–	w	+	w	w	–	–	NT
Glycerol	+	+	+	+	+	+	+	+	+	+	NT	NT	+	+	+	NT	+
Erythritol	-	–	–	–	–	–	–	+	+/(w*)	–	+	+	–	–	–	–	w
Sorbitol	-	–	S	+/S	+	+	+	+	+	+/w/S	NT	NT	+	+	S	NT	w
Ribitol	w	-	S	+/S	+	+	+	+	+	+/w/S	NT	NT	+	+	S	NT	w
D-Glucitol	w	+/D	+/w/S	–/w/S	V	V	+	–	–	+	NT	NT	+	–	w/S	–	w
D-Mannitol	w	+	+/w/S	–/w/S	V	+	+	–	–	+	NT	NT	+	–	+	+	+
Galactitol	-	w	–	–	–	–	–	+	w	–	NT	NT	–	–	–	NT	–
5-Keto-D-Gluconate	-	NT	NT	NT	NT	NT	NT	NT	NT	NT	NT	NT	NT	NT	NT	NT	NT
D-Gluconate	+	NT	NT	NT	NT	NT	NT	NT	NT	NT	NT	NT	NT	NT	NT	NT	NT
D-Glucoronate	+	+	+	+	+	+	+	+	+	+	+	+	+	+	+	+	+
D-Galacturonate	-	NT	NT	NT	NT	NT	NT	NT	NT	NT	NT	NT	NT	NT	NT	NT	NT
myo-Inositol	–	–	–	–	–	–	–	+	+/(w*)	–	–	–	–	–	–	+	–
Potassium-2-ketogluconate	+	+	NT	NT	NT	+	+	NT	NT	NT	NT	NT	NT	NT	NT	NT	NT
DL-Lactate	-	+	S	V(–/w/S*)	+	V	w	–	–	+(+/S*)	–	+	–	w	D	–	w
Succinate	w	–	+	+	+/w	+	+	+	w	+	NT	NT	w	+	+	NT	+
Citrate	-	–	–	–	–	–	–	–	–	–	NT	NT	–	–	–	NT	–
Methanol	w	D	–	–	–	–	NT	–	–	–	NT	NT	–	–	–	NT	–
Ethanol	+	–	+	+	+	V	w	+	+	+	NT	NT	+	+	+	NT	+
D-Glucarate	+	NT	NT	NT	NT	NT	NT	NT	NT	NT	NT	NT	NT	NT	NT	NT	NT
L-malic acid	+	NT	NT	NT	NT	NT	NT	NT	NT	NT	NT	NT	NT	NT	NT	NT	NT
L-tartaric acid	-	NT	NT	NT	NT	-	-	NT	NT	NT	NT	NT	NT	NT	NT	NT	NT
D-tartaric acid	-	NT	NT	NT	NT	NT	NT	NT	NT	NT	NT	NT	NT	NT	NT	NT	NT
Cycloheximide (0.01%)	+	+	S	S	–	NT	+/D	+	+	w/S	NT	NT	–	–	–	NT	w
**Assimilation of nitrogen compounds**																	
Nitrate	-	d/w	-	-	-	-	-	-	-	-	-	-	-	-	-	-	-
Nitrite	-	-	-	-	-	-	-	-	-	-	NT	NT	-	-	-	NT	w
L-lysine	+	+	NT	NT	NT	-	-	NT	NT	NT	NT	NT	NT	NT	NT	NT	+
Ethylamine hydrochloride	-	d	NT	NT	NT	-	-	NT	NT	NT	NT	NT	NT	NT	NT	NT	+
**other tests:**																	
Growth with 10% NaCl	w	NT	NT	NT	NT	-	NT	NT	NT	NT	NT	NT	NT	NT	NT	NT	NT
Growth with 8% NaCl	-	NT	NT	NT	NT	+	NT	NT	NT	NT	NT	NT	NT	NT	NT	NT	NT
Growth with 5% NaCl	+	NT	NT	NT	NT	+	NT	NT	NT	NT	NT	NT	NT	NT	NT	NT	NT
Growth with 60% glucose	-	NT	NT	NT	NT	NT	NT	NT	NT	NT	NT	NT	NT	NT	NT	NT	NT
Growth with 50% glucose	-	NT	NT	NT	NT	-	NT	NT	NT	NT	NT	NT	NT	NT	NT	NT	NT
Growth at 25C	+	+	+	+	+	+	+	+	+	+	+	+	+	+	+	+	Vw
Growth at 30C	+	+	+	+	+	–	–	+	+	V	+	+	+/w	–	+	+	–
Growth at 35C	w	+	–	V	–	–	NT	+	+	–	NT	NT	NT	NT	–	–	NT
Growth at 37C	-	-	–	–	–	–	–	+	+	–	–	+	–	NT	–	–	NT

*Data from Sampaio et al. 2011; Yurkov et al. 2015; Tsuji et al. 2017; Turchetti et al. 2018. +, Positive; –, negative; D, delayed positive; W, weak positive; S, slow; V, variable.

Supplemental Table ST6. The identity reports on the percentage of base pairs of the seven marker loci sequence between *C. onofrii* and all available *Cystobasidium* strains

		**Percent identity**						
		Cystobasidium onofrii sp. nov. FKI-L6-BK-PAB1						
**Strain number**	**Taxon name**	**ITS**	**LSU**	**SSU**	***CYTB***	***RPB1***	***RPB2***	***TEF1***
JCM 10901	*Cystobasidium benthicum*	97,01%	99,22%	99,63%	91,29%	83,65%	87,23%	92,89%
JCM 31526	*Cystobasidium tubakii*	96,45%	97,95%	99,39%	90,31%	83,16%	87,23%	90,37%
CBS9 130	*Cystobasidium pinicola*	95,66%	98,41%	99,20%	92,91%	79,60%	85,82%	90,27%
CBS 12733	*Cystobasidium portillinense*	/	98,33%	/	/	/	/	/
JCM 10953	*Cystobasidium laryngis*	95,51%	97,65%	99,20%	93,18%	<75%	84,04%	90,19%
CBS 12324	*Cystobasidium ritchiei*	95,32%	97,40%	<75%	/	/	/	88,51%
JCM 31527	*Cystobasidium ongulense*	94,94%	97,29%	99,14%	93,18%	<75%	<75%	89,01%
CBS 12623	*Cystobasidium oligophagum*	93,82%	97,19%	98,83%	/	/	/	/
JCM 5951	*Cystobasidium lysinophilum*	94,76%	95,92%	99,08%	92,47%	<75%	82,42%	90,88%
JCM 10899	*Cystobasidium calyptogenae*	93,84%	95,77%	98,71%	<75%	<75%	<75%	89,81%
JCM 3780	*Cystobasidium pallidum*	93,31%	98,57%	98,96%	91,76%	81,04%	86,88%	91,11%
CBS 15650	*Cystobasidium terricola*	93,06%	94,57%	98,96%	90,85%	<75%	84,05%	87,90%
JCM 10954	*Cystobasidium slooffiae*	92,48%	95,40%	98,89%	88,47%	<75%	<75%	88,51%
CBS 15509	*Cystobasidium raffinophilum*	92,11%	95,81%	98,83%	89,88%	<75%	<75%	86,69%
DBVPG 10041	*Cystobasidium alpinum*	94,75%	96,39%	/	/	/	/	/
PYCC 6649	*Cystobasidium fimetarium*	93,43%	95,91%	98,53%	/	/	/	83,19%
CBS 11769	*Cystobasidium psychroaquaticum*	93,12%	93,43%	100,00%	/	/	/	87,25%
CBS 319	*Cystobasidium minutum*	92,09%	96,51%	98,28%	<75%	<75%	84,26%	89,54%
CBS 11769	*Rhodotorula mucilaginosa*	90,21%	81,57%	91,08%	<75%	<75%	<75%	82,92%
CBS 8477	*Naohidea sebacea*	89,38%	89,15%	95,22%	<75%	<75%	<75%	90,86%
CBS 8253	*Erythrobasidium hasegawianum*	85,54%	94,30%	96,81%	<75%	<75%	<75%	82,31%

/ Not Available

Supplementary Table ST7. Genomic features of the *Cystobasidium* species

Taxon name	*Cystobasidium onofrii*	*Cystobasidium ongulense*	*Cystobasidium pallidum*	*Cystobasidium tubakii*	*Cystobasidium slooffiae*
**Isolate**	FKI-L6-BK-PAB1	9A-5	JCM_3780	9A-1_01	I2-R3
**GeneBank ID**	GCA_022813105.1	GCA_022835575.1	GCA_001599955.1	GCA_024345325.1	GCA_019775285.1
**locus_tag**	OHC18	9A500	JCM378	9A101	I2R33
**Assembly Size (bp)**	20,994,657	19,884,178	21,702,704	21,538,963	22,069,978
**Largest Scaffold (bp)**	3,147,288	3,150,687	2,538,488	5,533,463	1,614,568
**Average Scaffold (bp)**	129,597	397,684	328,829	4,307,793	204,352
**Num Scaffolds**	162	50	66	5	108
**Scaffold N50 (bp)**	616,502	2,125,609	1,290,484	5,242,860	1,100,933
**Percent GC (%)**	48.76	49.22	49.66	50.09	49.33
**Num Genes**	6,889	6,732	6,293	6,564	7,067
**Num Proteins**	6,831	6,694	6,242	6,529	7,006
**Num tRNA**	58	38	51	35	61
**Unique Proteins**	691	754	728	610	983
**Prots atleast 1 ortholog**	5,891	5,739	5,353	5,718	5,736
**Single-copy orthologs**	3,461	3,461	3,461	3,461	3,461

Supplementary Table ST8. Data summary table from multiple variables.

Strain	group1	group2	df	p	p.adj	p.adj.signif
Cystobasidium onofrii	1000 J/m^2	2000 J/m^2	3.34548	3.80E-02	2.66E-01	ns
Cystobasidium onofrii	1000 J/m^2	3000 J/m^2	2.637825	3.00E-03	4.20E-02	*
Cystobasidium onofrii	1000 J/m^2	500 J/m^2	2.495751	2.58E-01	7.74E-01	ns
Cystobasidium onofrii	1000 J/m^2	N	2.134989	3.80E-02	2.66E-01	ns
Cystobasidium onofrii	2000 J/m^2	3000 J/m^2	3.436264	7.40E-04	1.18E-02	*
Cystobasidium onofrii	2000 J/m^2	500 J/m^2	3.177608	4.00E-03	4.80E-02	*
Cystobasidium onofrii	2000 J/m^2	N	2.346866	3.00E-03	4.20E-02	*
Cystobasidium onofrii	3000 J/m^2	500 J/m^2	3.93291	1.52E-05	3.93E-04	***
Cystobasidium onofrii	3000 J/m^2	N	2.791687	6.84E-05	1.50E-03	**
Cystobasidium onofrii	500 J/m^2	N	3.001291	4.00E-03	4.80E-02	*
Naganishia onofrii	1000 J/m^2	2000 J/m^2	2.203532	1.13E-01	4.52E-01	ns
Naganishia onofrii	1000 J/m^2	3000 J/m^2	3.433146	1.27E-04	2.54E-03	**
Naganishia onofrii	1000 J/m^2	500 J/m^2	2.049583	2.61E-01	7.74E-01	ns
Naganishia onofrii	1000 J/m^2	N	3.834591	3.34E-04	6.01E-03	**
Naganishia onofrii	2000 J/m^2	3000 J/m^2	2.476449	2.30E-02	2.07E-01	*
Naganishia onofrii	2000 J/m^2	500 J/m^2	2.917868	8.60E-02	4.30E-01	**
Naganishia onofrii	2000 J/m^2	N	2.309176	1.50E-02	1.50E-01	**
Naganishia onofrii	3000 J/m^2	500 J/m^2	2.117376	2.90E-02	2.32E-01	ns
Naganishia onofrii	3000 J/m^2	N	3.820182	1.51E-05	3.93E-04	***
Naganishia onofrii	500 J/m^2	N	2.075563	6.09E-01	7.74E-01	ns
Naganishia kalamii	1000 J/m^2	2000 J/m^2	3.82412	8.91E-04	1.34E-02	*
Naganishia kalamii	1000 J/m^2	3000 J/m^2	3.537664	6.31E-06	1.77E-04	***
Naganishia kalamii	1000 J/m^2	500 J/m^2	3.531983	6.20E-04	1.05E-02	*
Naganishia kalamii	1000 J/m^2	N	3.531983	1.62E-05	3.93E-04	***
Naganishia kalamii	2000 J/m^2	3000 J/m^2	3.1111	9.75E-05	2.05E-03	**
Naganishia kalamii	2000 J/m^2	500 J/m^2	3.105774	2.05E-04	3.90E-03	**
Naganishia kalamii	2000 J/m^2	N	3.105774	2.88E-05	6.62E-04	***
Naganishia kalamii	3000 J/m^2	500 J/m^2	3.999967	2.81E-07	8.15E-06	****
Naganishia kalamii	3000 J/m^2	N	3.999967	7.51E-08	2.25E-06	****
Naganishia kalamii	500 J/m^2	N	4	1.19E-05	3.21E-04	***

Significant differences were calculated by t-test with $*p > 0.05$; $**p > 0.001$; $***\ p > 0.0001$ and $****\ p > 0.00001$
