# Supplementary material for: Genomic characterization and radiation tolerance of Naganishia kalamii sp. nov. and Cystobasidium onofrii sp. nov. from Mars 2020 mission assembly facilities
Source: IMA Fungus. 2023 Aug 11;14:15. doi: 10.1186/s43008-023-00119-4 (PMC10422843; doi:10.1186/s43008-023-00119-4)
Supplement: Supplementary file 2 — Additional file 2: Fig. S1. Maximum likelihood phylogenetic tree, based on concatenated ITS and LSU gene sequences of N. kalamii and related Naganishia species, class Tremellomycetes, order Filobasidiales. The tree was rooted with G. gastricus type strains. Bootstrap percentages from 1000 replications are shown on the branches (values below 50.00 are not shown). GenBank accession numbers of the sequences are listed in Additional file 1: Table S1. Fig. S2. Pairwise sequence genetic distance matrix based on LSU (below the diagonal) and ITS (above the diagonal) gene sequences of N. kalamii and related Naganishia species (albida clade highlighted). The function dist.dna in the ape: Analyses of Phylogenetics and Evolution package (R software) with the Kimura 2-parameter substitution model was used for the generation of the matrix. Fig. S3. SSU phylogenetic tree of N. kalamiii and related taxa of N. albida clade (shown only in part), class Tremellomycetes, order Filobasidiales. The tree was rooted with G. gastricus type strains. Bootstrap percentages from 1000 replications are shown on the branches (values below 50.00 are not shown). GenBank accession numbers of the sequences are listed in Additional file 1: Table S1. Fig. S4. CYTB phylogenetic tree of N. kalamiii and related taxa of N. albida clade (shown only in part), class Tremellomycetes, order Filobasidiales. The tree was rooted with G. gastricus type strains. Bootstrap percentages from 1000 replications are shown on the branches (values below 50.00 are not shown). GenBank accession numbers of the sequences are listed in Additonal file 1: Table S1. Fig. S5. TEF1 phylogenetic tree of N. kalamiii and related taxa of N. albida clade (shown only in part), class Tremellomycetes, order Filobasidiales. The tree was rooted with G. gastricus type strains. Bootstrap percentages from 1000 replications are shown on the branches (values below 50.00 are not shown). GenBank accession numbers of the sequences are listed in Addition [file 43008_2023_119_MOESM2_ESM.pdf]

Supplementary figures

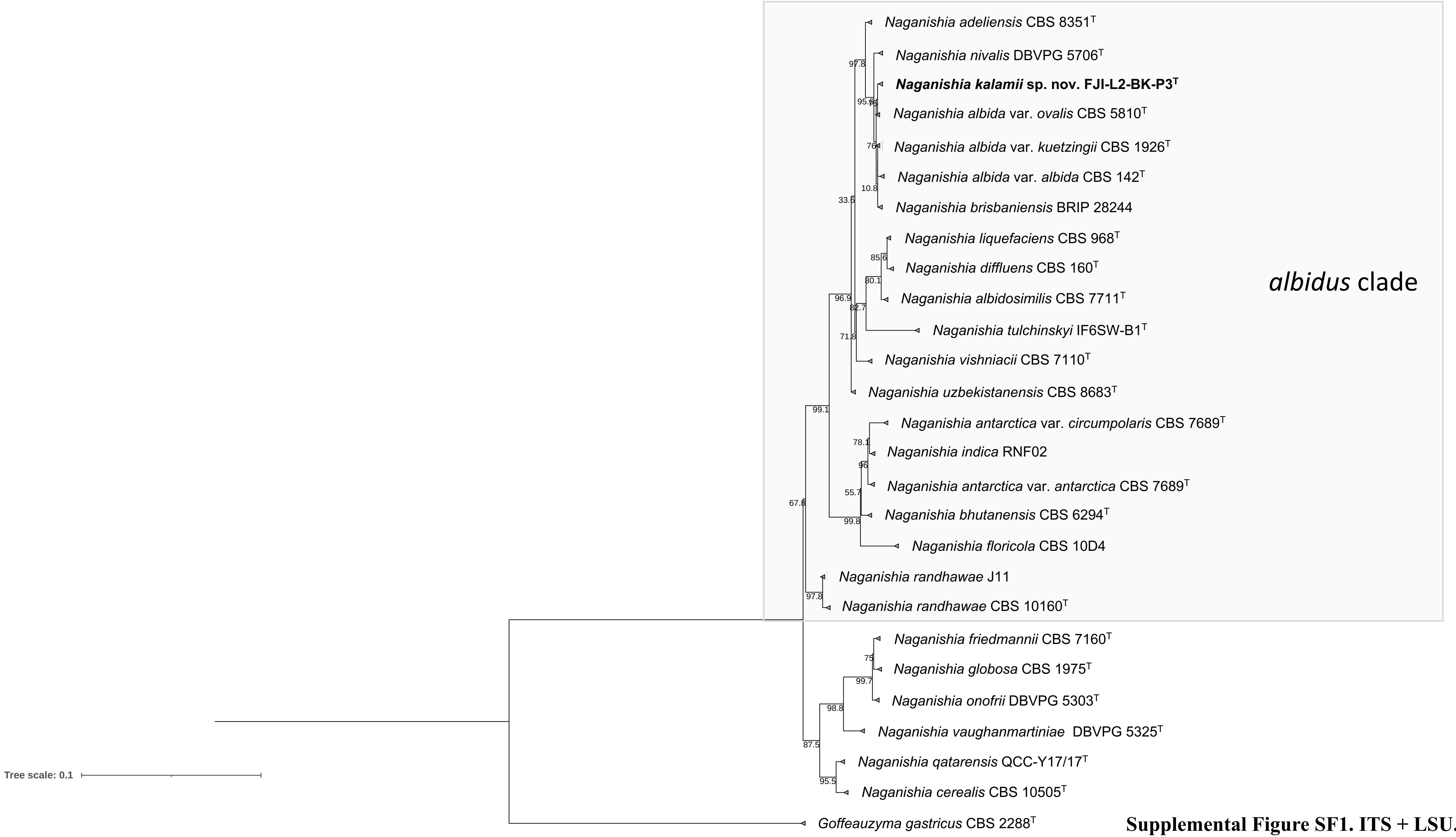

Supplemental Figure SF1. ITS + LSU.

Supplemental Figure SF2

LSU

ITS

	Naganishia_kalamii_FJI-L2-BK-P3	Naganishia_albida_var_ovalis_CBS5810	Naganishia_albida_var_kuetzingii_CBS1926	Naganishia_albida_var_albida_CBS142	Naganishia_adeliensis_CBS8351	Naganishia_nivalis_DBVPG5706	Naganishia_brisbanensis_BRIP_28244	Naganishia_albidosimilis_CBS7711	Naganishia_liquefaciens_CBS968	Naganishia_diffluens_CBS160	Naganishia_friedmannii_CBS7160	Naganishia_globosa_CBS1975	Naganishia_tulchinskyi_IF6SW-B1	Naganishia_randhawae_J11	Naganishia_uzbekistanensis_CBS8683	Naganishia_onofrii_DBVPG5303	Naganishia_vishniacii_CBS7110	Naganishia_qatarensis_QCC-Y17/17	Naganishia_randhawae_CBS10160	Naganishia_vaughanmartiniae_DBVPG4736	Naganishia_cerealis_CBS10505	Naganishia_floricola_10D4	Naganishia_bhutanensis_CBS6294	Naganishia_antarctica_var_circumpolaris_CBS7689	Naganishia_antarctica_var_antarcticus_CBS7687	Goffeauzyma_gastricus_CBS2288
Naganishia_kalamii_FJI-L2-BK-P3		**0.000**	**0.002**	**0.005**	**0.008**	**0.002**	**0.003**	**0.025**	**0.025**	**0.023**	0.104	0.104	0.121	0.054	0.000	0.102	0.015	0.091	0.062	0.103	0.084	0.035	0.028	0.032	0.038	0.350
Naganishia_albida_var_ovalis_CBS5810	**0.044**		0.002	0.005	0.008	0.002	0.003	0.025	0.025	0.023	0.104	0.104	0.121	0.054	0.000	0.102	0.015	0.091	0.062	0.103	0.084	0.035	0.028	0.032	0.038	0.350
Naganishia_albida_var_kuetzingii_CBS1926	**0.045**	0.042		0.003	0.006	0.004	0.002	0.025	0.025	0.023	0.106	0.106	0.121	0.056	0.000	0.104	0.015	0.093	0.064	0.105	0.086	0.037	0.029	0.034	0.040	0.352
Naganishia_albida_var_albida_CBS142	**0.047**	0.044	0.042		0.010	0.007	0.005	0.028	0.028	0.026	0.108	0.108	0.125	0.059	0.000	0.106	0.015	0.093	0.067	0.107	0.086	0.040	0.033	0.037	0.044	0.352
Naganishia_adeliensis_CBS8351	**0.055**	0.052	0.054	0.055		0.009	0.008	0.028	0.028	0.026	0.106	0.106	0.123	0.056	0.000	0.106	0.018	0.093	0.064	0.105	0.086	0.037	0.031	0.037	0.040	0.350
Naganishia_nivalis_DBVPG5706	**0.055**	0.051	0.053	0.055	0.059		0.006	0.021	0.021	0.019	0.092	0.089	0.057	0.035	0.003	0.095	0.013	0.075	0.042	0.090	0.073	0.033	0.013	0.018	0.023	0.348
Naganishia_brisbanensis_BRIP_28244	**NA**	NA	NA	NA	NA	NA		0.027	0.027	0.026	0.111	0.113	0.126	0.060	NA	0.108	0.017	0.095	0.068	0.110	0.089	0.038	0.032	0.037	0.043	0.372
Naganishia_albidosimilis_CBS7711	**0.060**	0.064	0.066	0.067	0.064	0.064	NA		0.000	0.002	0.101	0.100	0.109	0.059	0.000	0.102	0.023	0.089	0.067	0.101	0.089	0.037	0.039	0.037	0.040	0.357
Naganishia_liquefaciens_CBS968	**0.064**	0.066	0.067	0.069	0.066	0.066	NA	0.052		0.002	0.101	0.100	0.109	0.059	0.000	0.102	0.023	0.089	0.067	0.101	0.089	0.037	0.039	0.037	0.040	0.357
Naganishia_diffluens_CBS160	**0.066**	0.069	0.071	0.072	0.069	0.070	NA	0.054	0.044		0.102	0.102	0.109	0.058	0.000	0.104	0.021	0.089	0.066	0.101	0.089	0.038	0.041	0.039	0.042	0.354
Naganishia_friedmannii_CBS7160	0.081	0.078	0.079	0.081	0.045	0.085	NA	0.088	0.090	0.093		0.011	0.182	0.071	0.088	0.011	0.101	0.079	0.077	0.077	0.083	0.090	0.092	0.086	0.085	0.363
Naganishia_globosa_CBS1975	0.079	0.076	0.078	0.079	0.044	0.085	NA	0.086	0.088	0.092	0.017		0.180	0.071	0.000	0.014	0.100	0.077	0.079	0.083	0.079	0.088	0.090	0.086	0.085	0.364
Naganishia_tulchinskyi_IF6SW-B1	NA	NA	NA	NA	NA	NA	NA	NA	NA	NA	NA	NA		0.135	0.047	0.181	0.119	0.172	0.146	0.200	0.186	0.101	0.119	0.124	0.136	0.459
Naganishia_randhawae_J11	0.088	0.084	0.086	0.084	0.047	0.095	NA	0.097	0.099	0.098	0.032	0.030	NA		0.000	0.075	0.052	0.055	0.006	0.085	0.057	0.036	0.039	0.040	0.044	0.346
Naganishia_uzbekistanensis_CBS8683	0.057	0.060	0.062	0.064	0.024	0.068	NA	0.062	0.062	0.064	0.044	0.042	NA	0.049		0.000	0.000	NA	NA	NA	NA	0.060	0.000	NA	NA	0.000
Naganishia_onofrii_DBVPG5303	0.080	0.077	0.078	0.080	0.043	0.085	NA	0.088	0.090	0.093	0.013	0.013	NA	0.029	0.041		0.100	0.082	0.082	0.089	0.088	0.084	0.090	0.090	0.089	0.369
Naganishia_vishniacii_CBS7110	0.059	0.060	0.062	0.062	0.030	0.064	NA	0.062	0.062	0.067	0.050	0.049	NA	0.059	0.030	0.048		0.089	0.060	0.099	0.080	0.033	0.033	0.027	0.033	0.350
Naganishia_qatarensis_QCC-Y17/17	0.081	0.078	0.077	0.081	0.047	0.083	NA	0.084	0.086	0.086	0.029	0.027	NA	0.032	0.045	0.025	0.045		0.060	0.076	0.015	0.072	0.072	0.072	0.070	0.377
Naganishia_randhawae_CBS10160	0.088	0.084	0.086	0.084	0.047	0.095	NA	0.097	0.099	0.098	0.032	0.030	NA	0.014	0.049	0.029	0.059	0.031		0.087	0.057	0.044	0.046	0.046	0.046	0.361
Naganishia_vaughanmartiniae_DBVPG4736	0.076	0.073	0.076	0.076	0.030	0.083	NA	0.085	0.088	0.090	0.004	0.002	NA	0.013	0.028	0.002	0.030	0.011	0.013		0.080	0.090	0.092	0.084	0.078	0.400
Naganishia_cerealis_CBS10505	0.087	0.083	0.085	0.087	0.051	0.084	NA	0.090	0.092	0.092	0.032	0.030	NA	0.035	0.049	0.030	0.049	0.022	0.035	0.017		0.066	0.066	0.062	0.060	0.394
Naganishia_floricola_10D4	0.109	0.106	0.107	0.106	0.079	0.108	NA	0.115	0.117	0.116	0.078	0.076	NA	0.081	0.078	0.082	0.081	0.081	0.084	0.062	0.072		0.013	0.010	0.011	0.356
Naganishia_bhutanensis_CBS6294	0.097	0.093	0.095	0.093	0.063	0.103	NA	0.102	0.102	0.102	0.068	0.067	NA	0.068	0.061	0.068	0.065	0.061	0.068	0.060	0.067	0.041		0.012	0.014	0.345
Naganishia_antarctica_var_circumpolaris_CBS7689	0.127	0.123	0.125	0.123	0.094	0.124	NA	0.131	0.131	0.131	0.099	0.098	NA	0.099	0.092	0.092	0.096	0.092	0.099	0.064	0.096	0.062	0.047		0.004	0.366
Naganishia_antarctica_var_antarcticus_CBS7687	0.095	0.092	0.093	0.092	0.066	0.101	NA	0.099	0.099	0.099	0.071	0.069	NA	0.071	0.064	0.069	0.067	0.064	0.071	0.062	0.069	0.047	0.026	0.044		0.398
Goffeauzyma_gastricus_CBS2288	0.183	0.179	0.181	0.183	0.191	0.198	NA	0.191	0.185	0.187	0.191	0.189	NA	0.191	0.193	0.197	0.195	0.185	0.191	0.124	0.200	0.226	0.217	0.256	0.217	

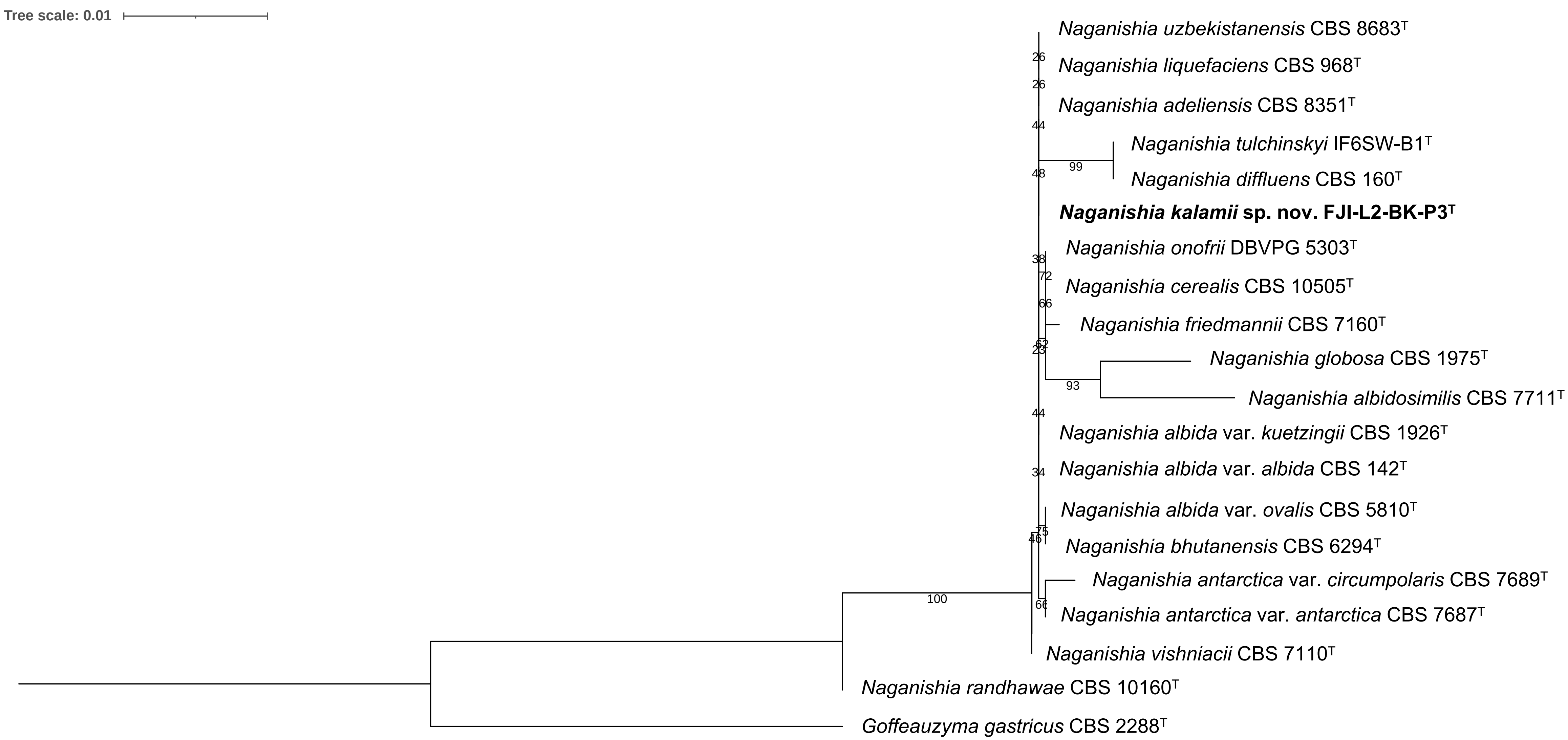

Supplemental Figure SF3. SSU

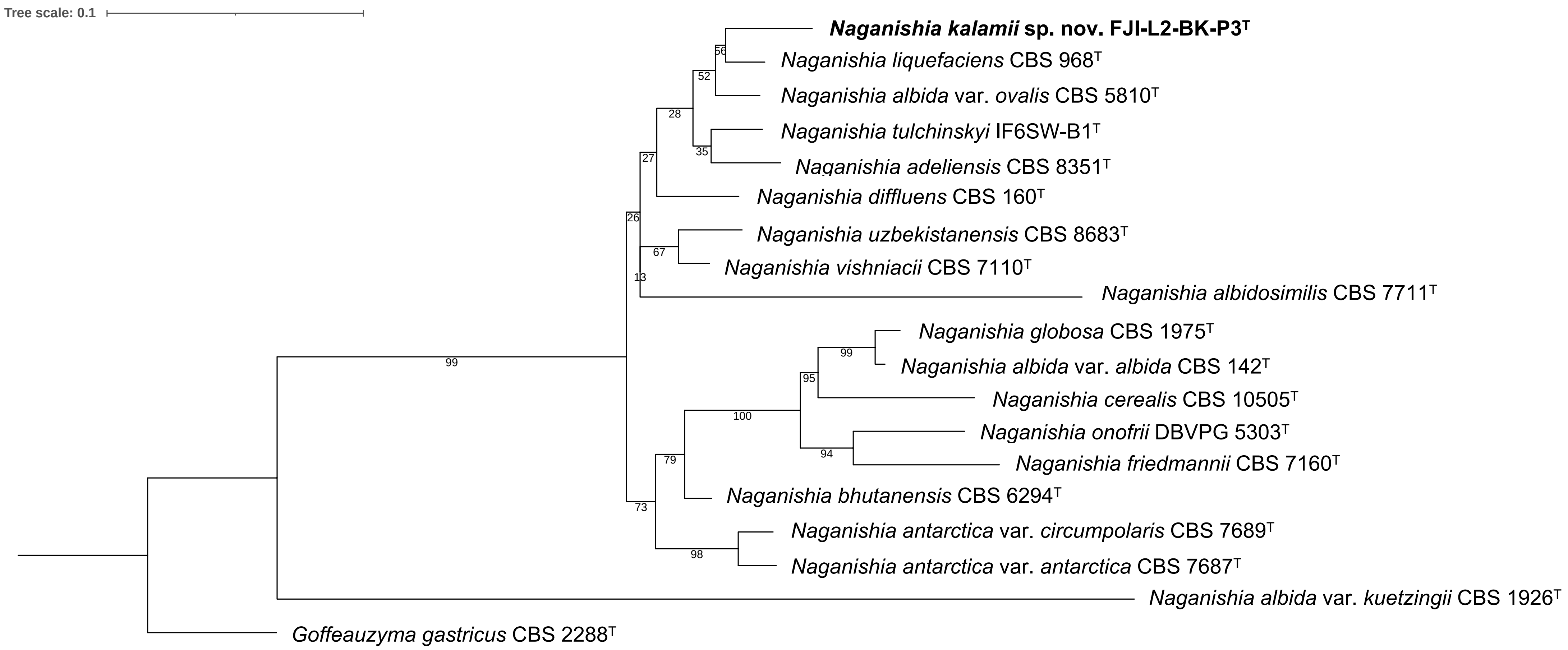

Supplemental Figure SF4. ***CYTB***

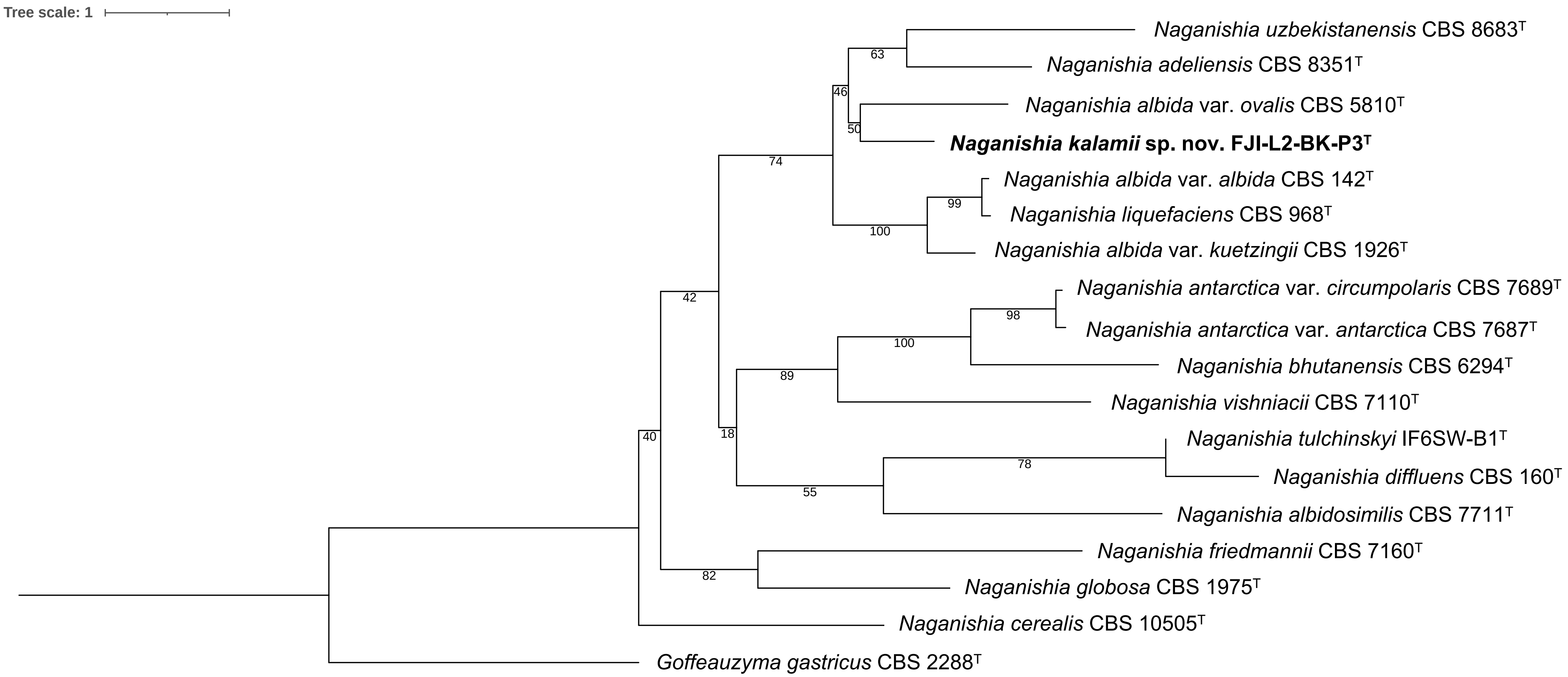

Supplemental Figure SF5. ***TEF1***

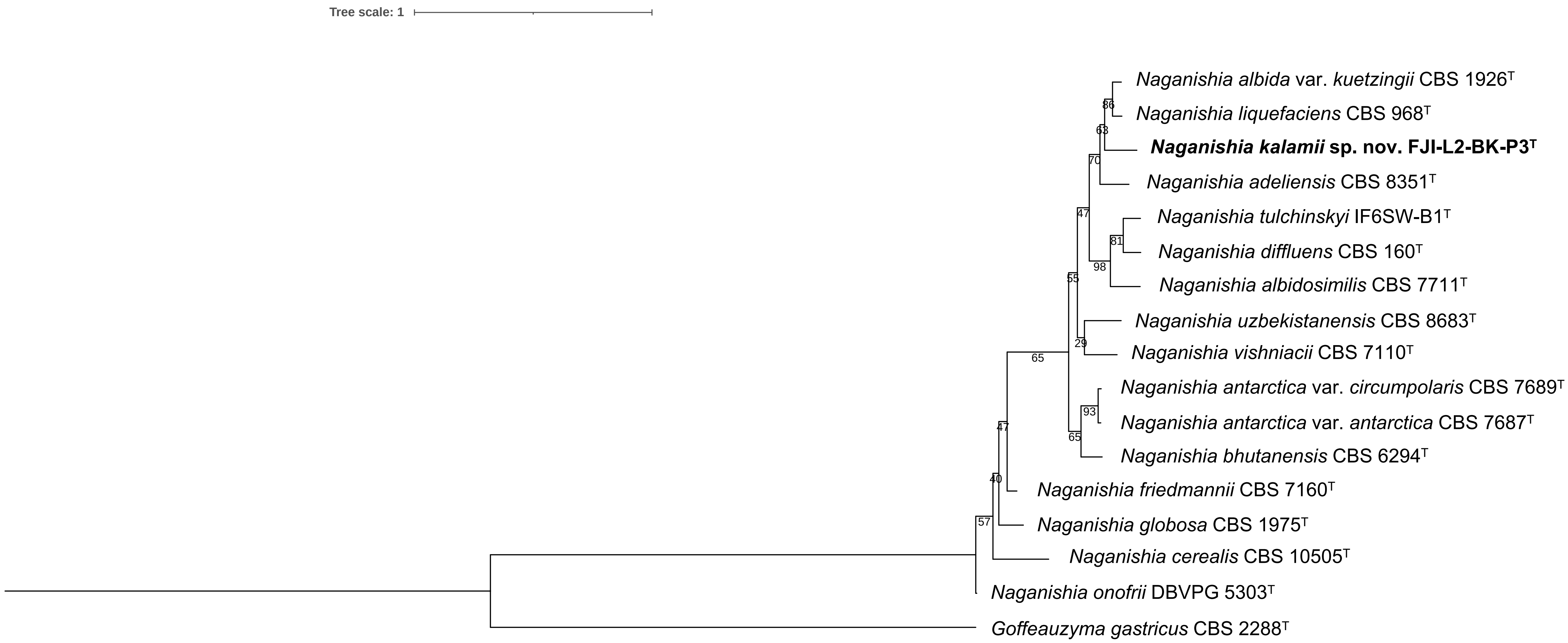

Supplemental Figure SF6. ***RPB1***

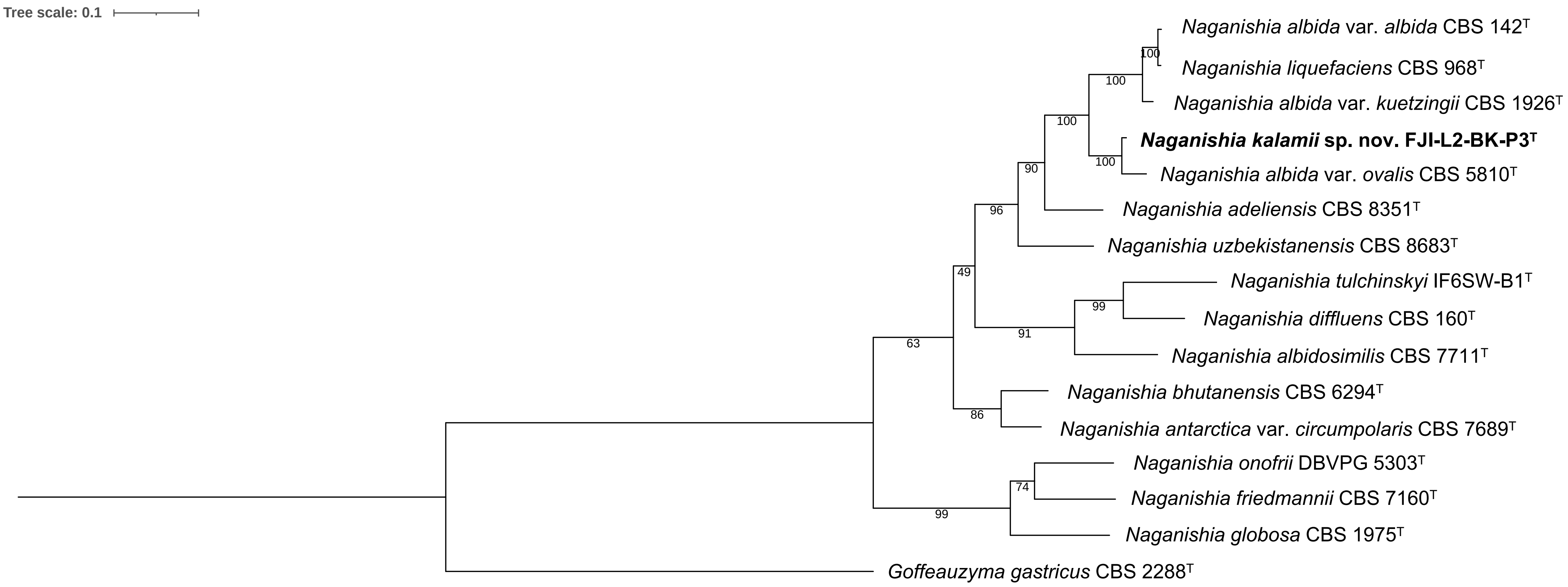

Supplemental Figure SF7. ***RPB2***

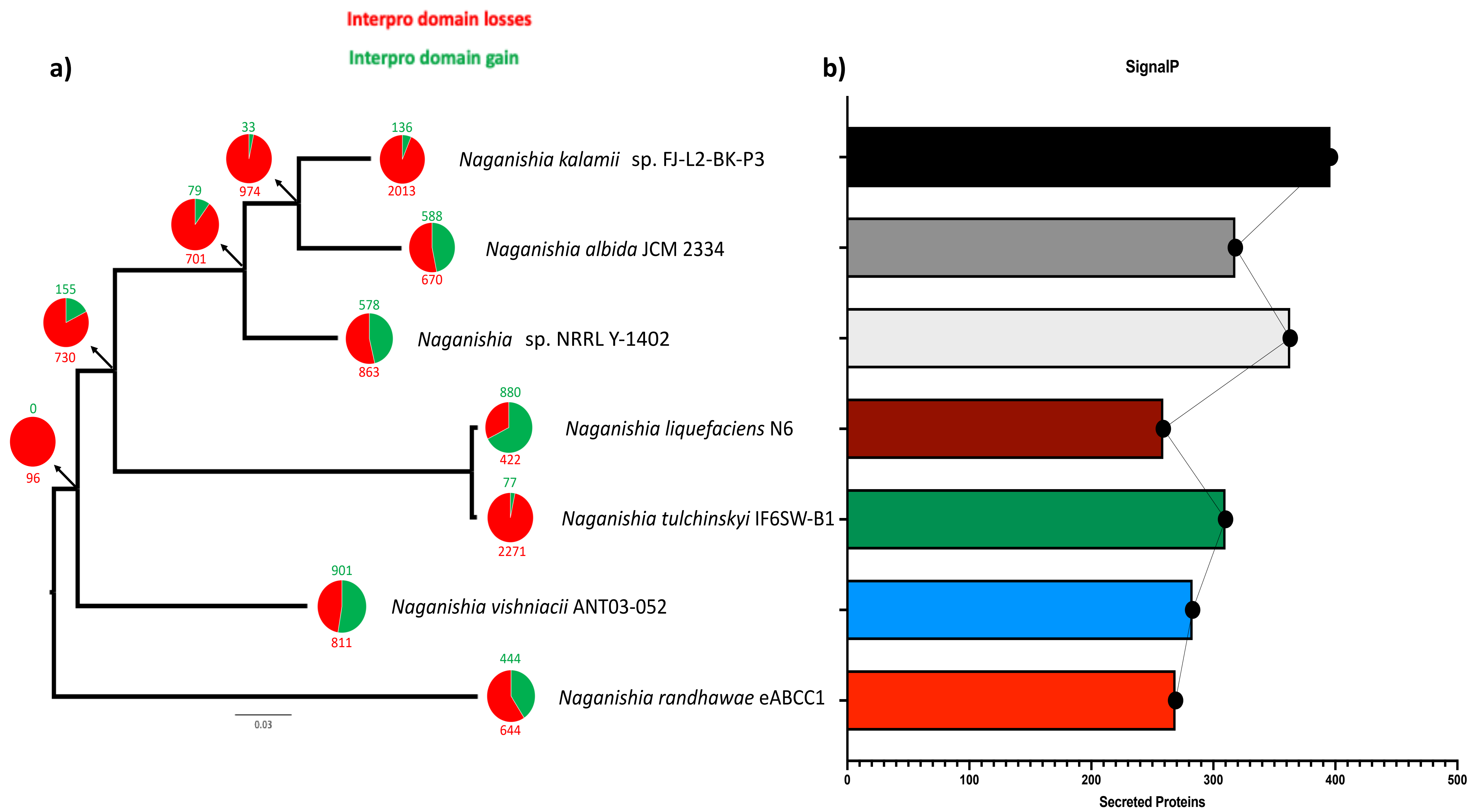

Supplemental Figure SF8

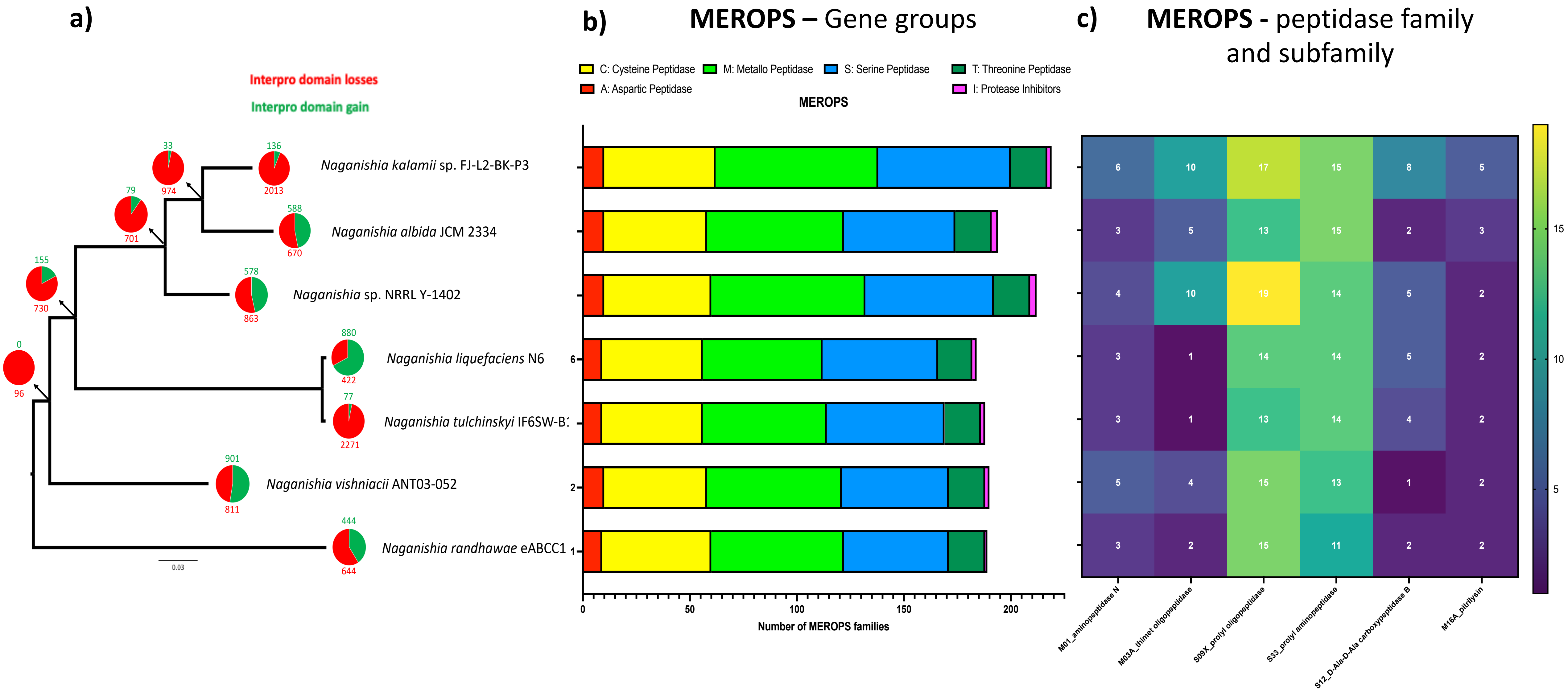

Supplemental Figure SF9.

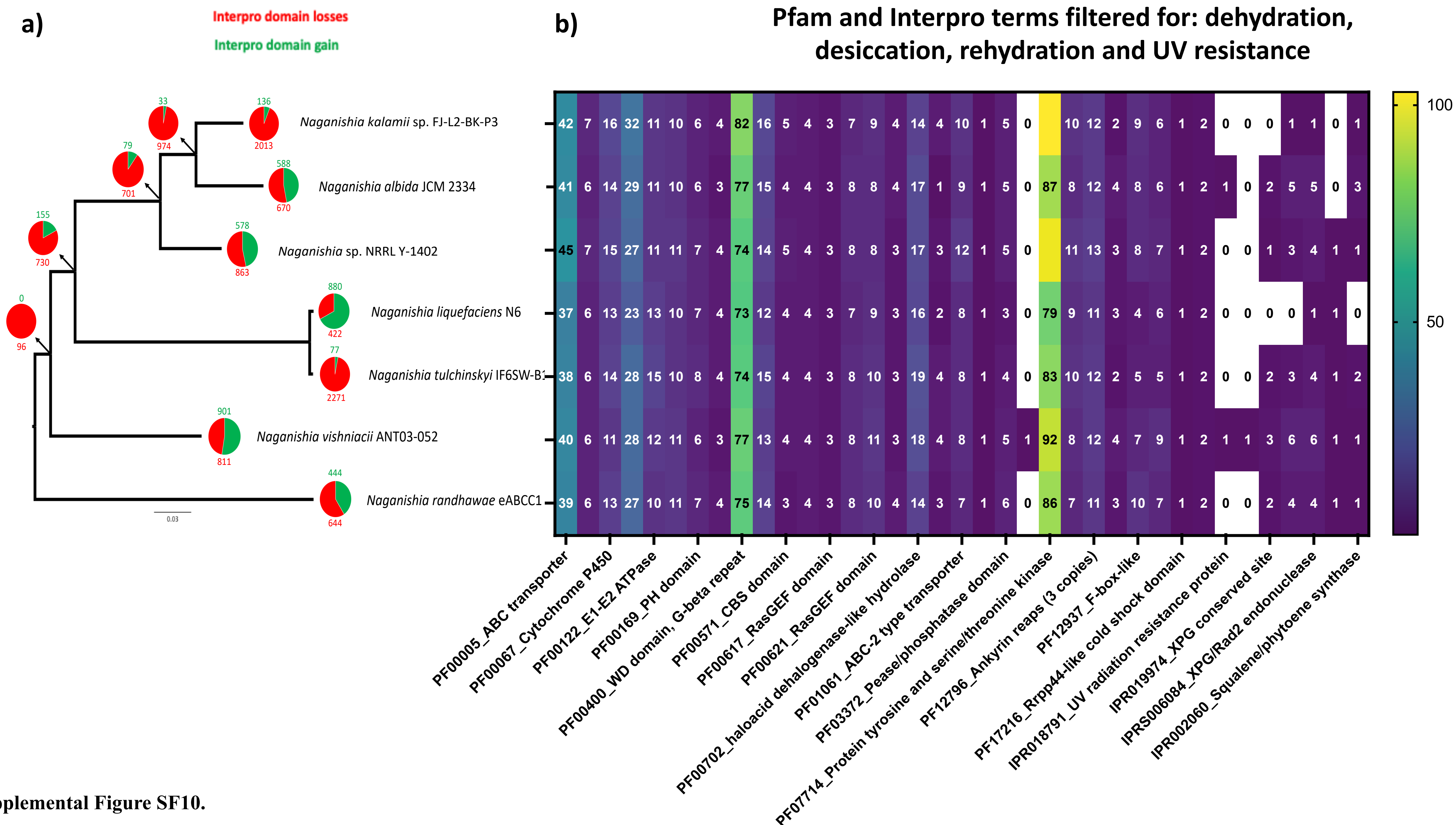

Supplemental Figure SF10.

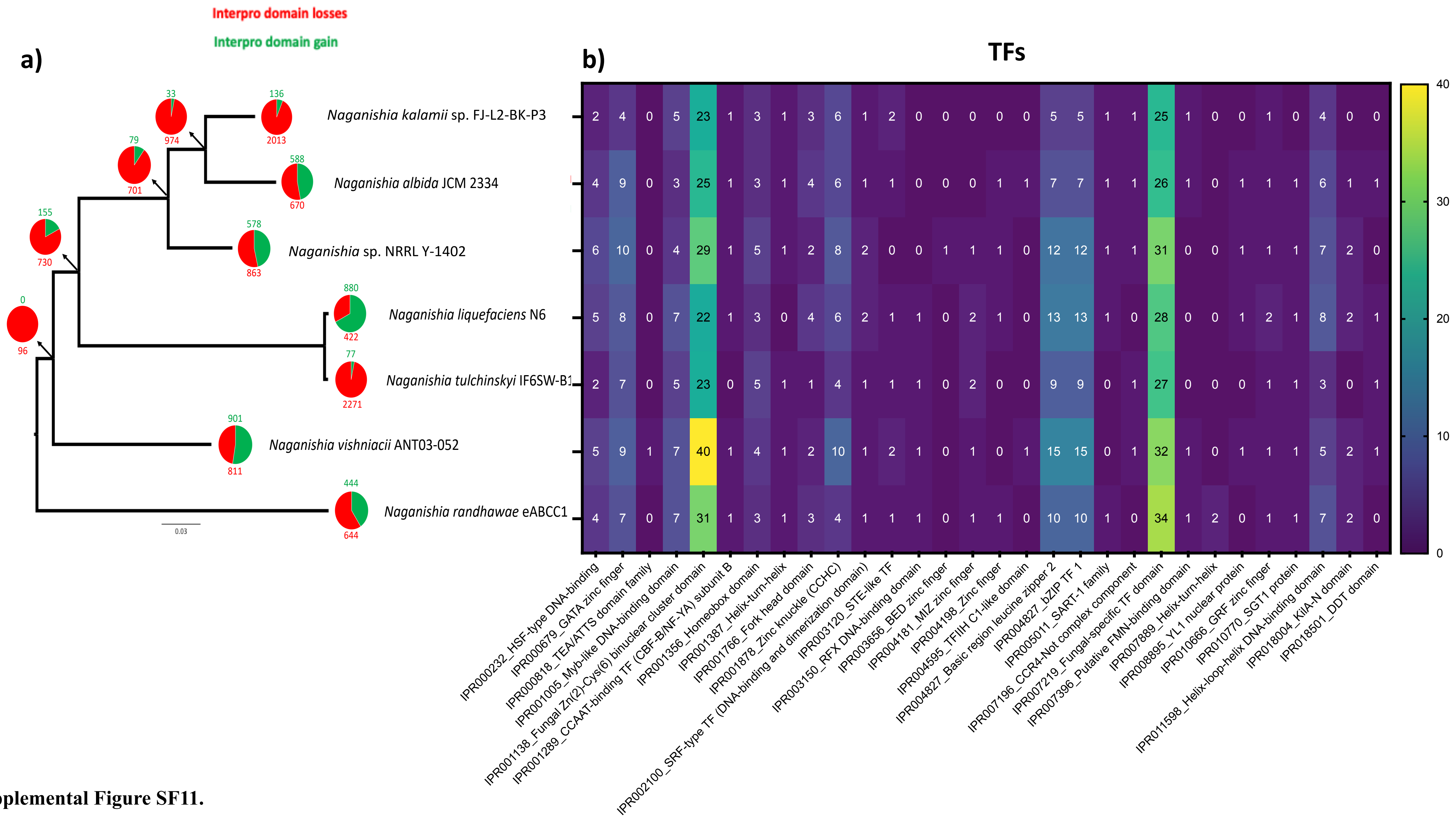

Supplemental Figure SF11.

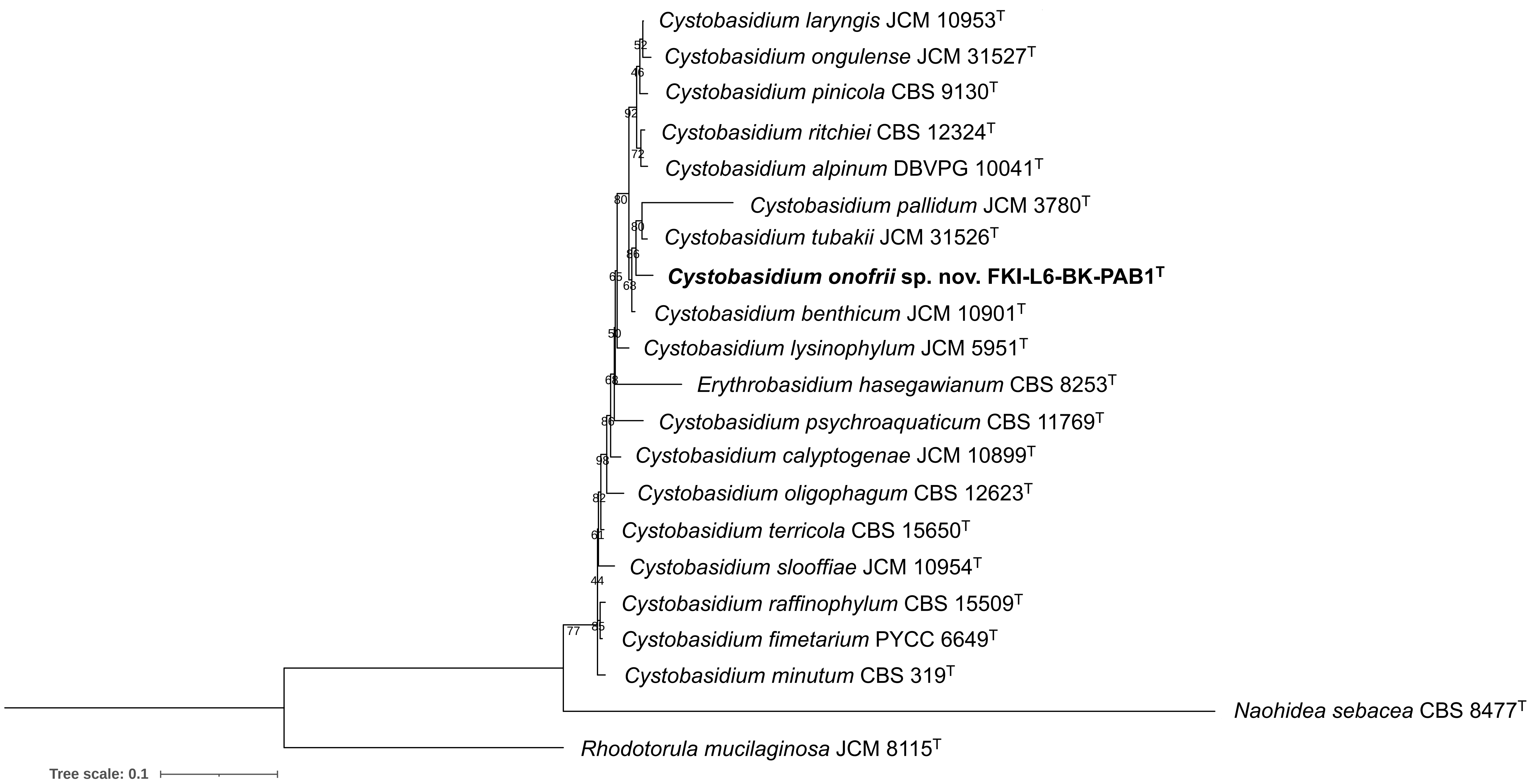

Supplemental Figure SF12. ITS

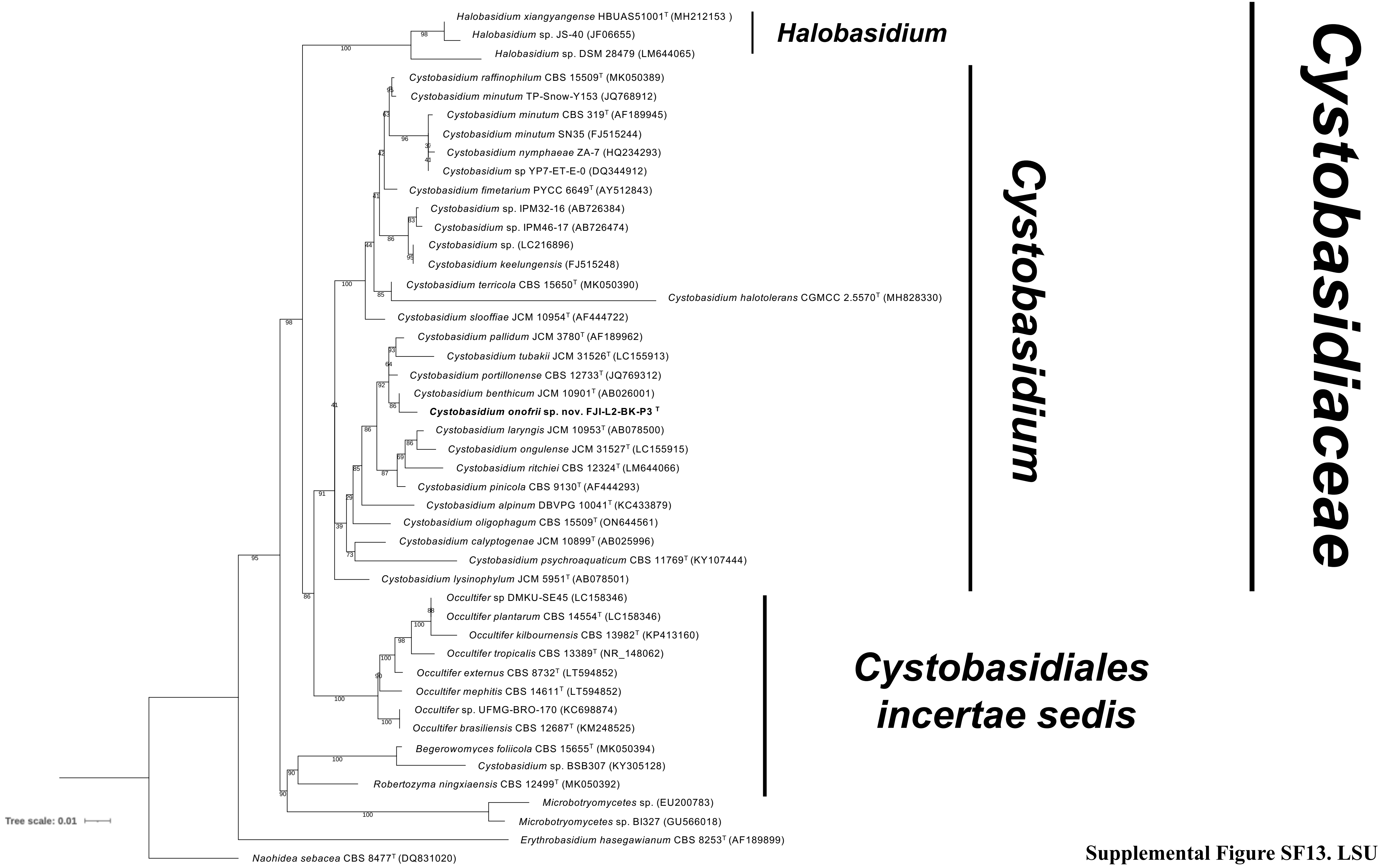

Supplemental Figure SF13. LSU

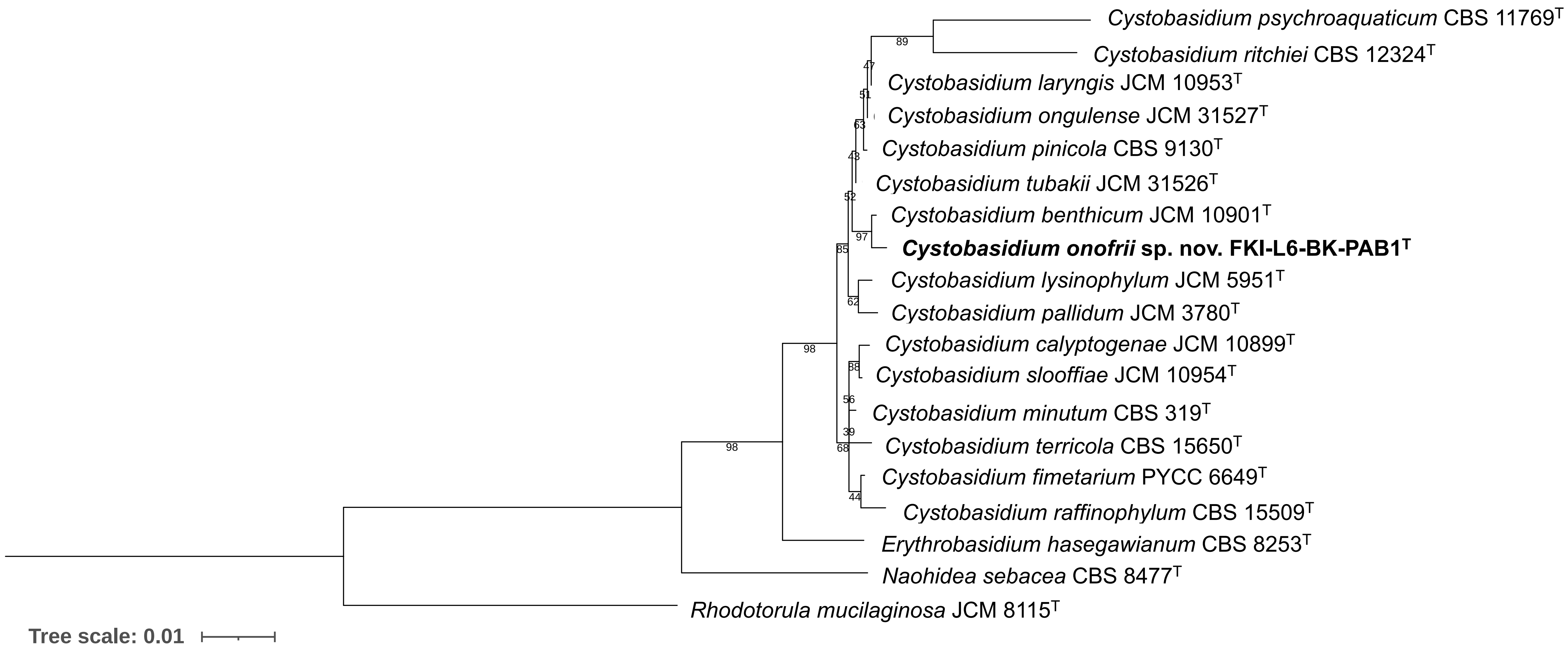

Supplemental Figure SF14. SSU

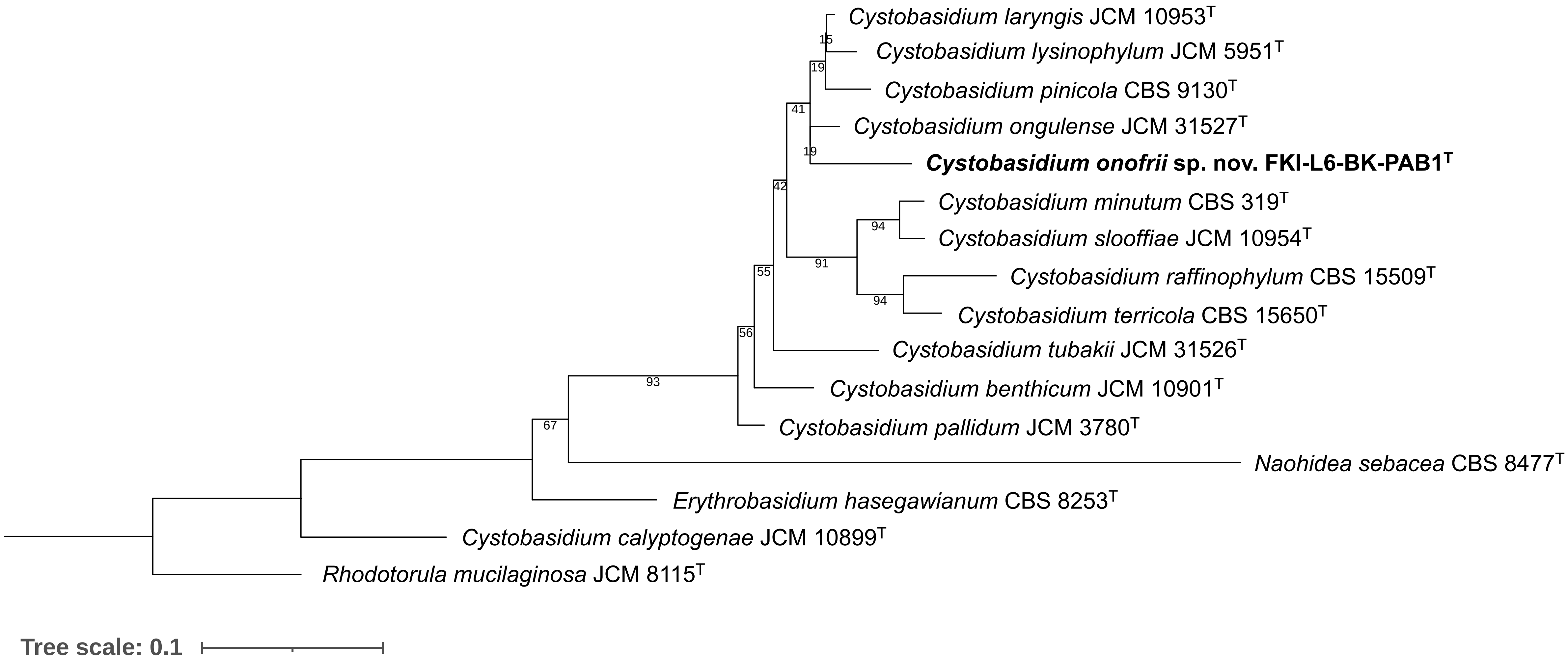

Supplemental Figure SF15. ***CYTB***

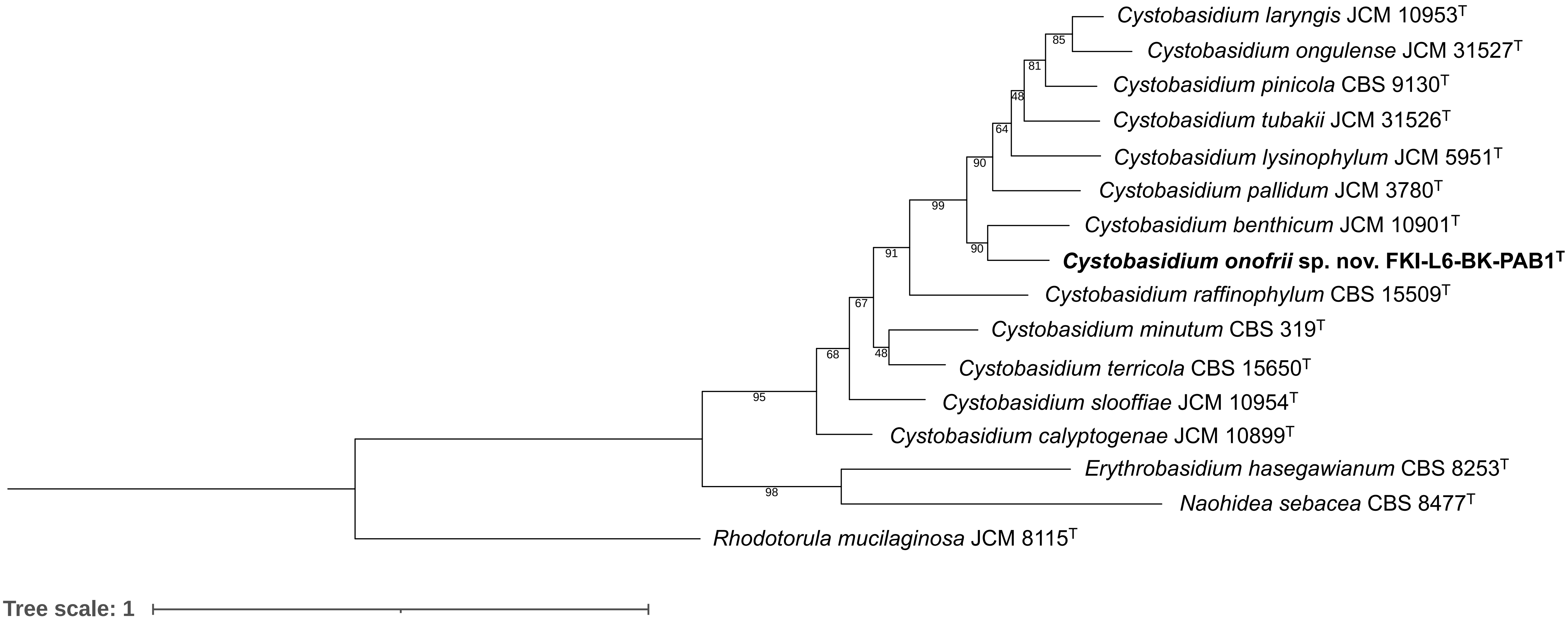

Supplemental Figure SF16. ***TEF1***

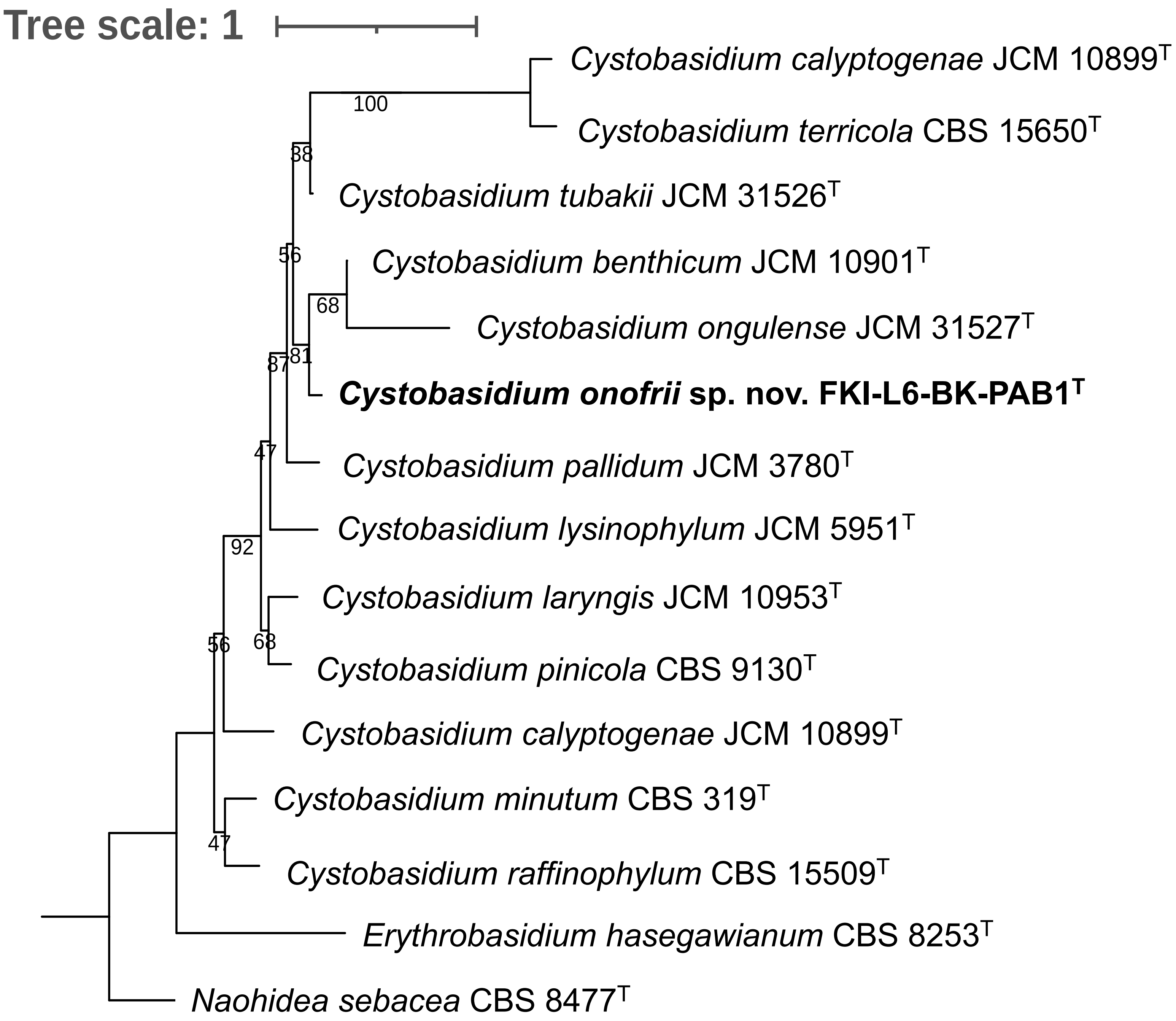

Supplemental Figure SF17. ***RPB1***

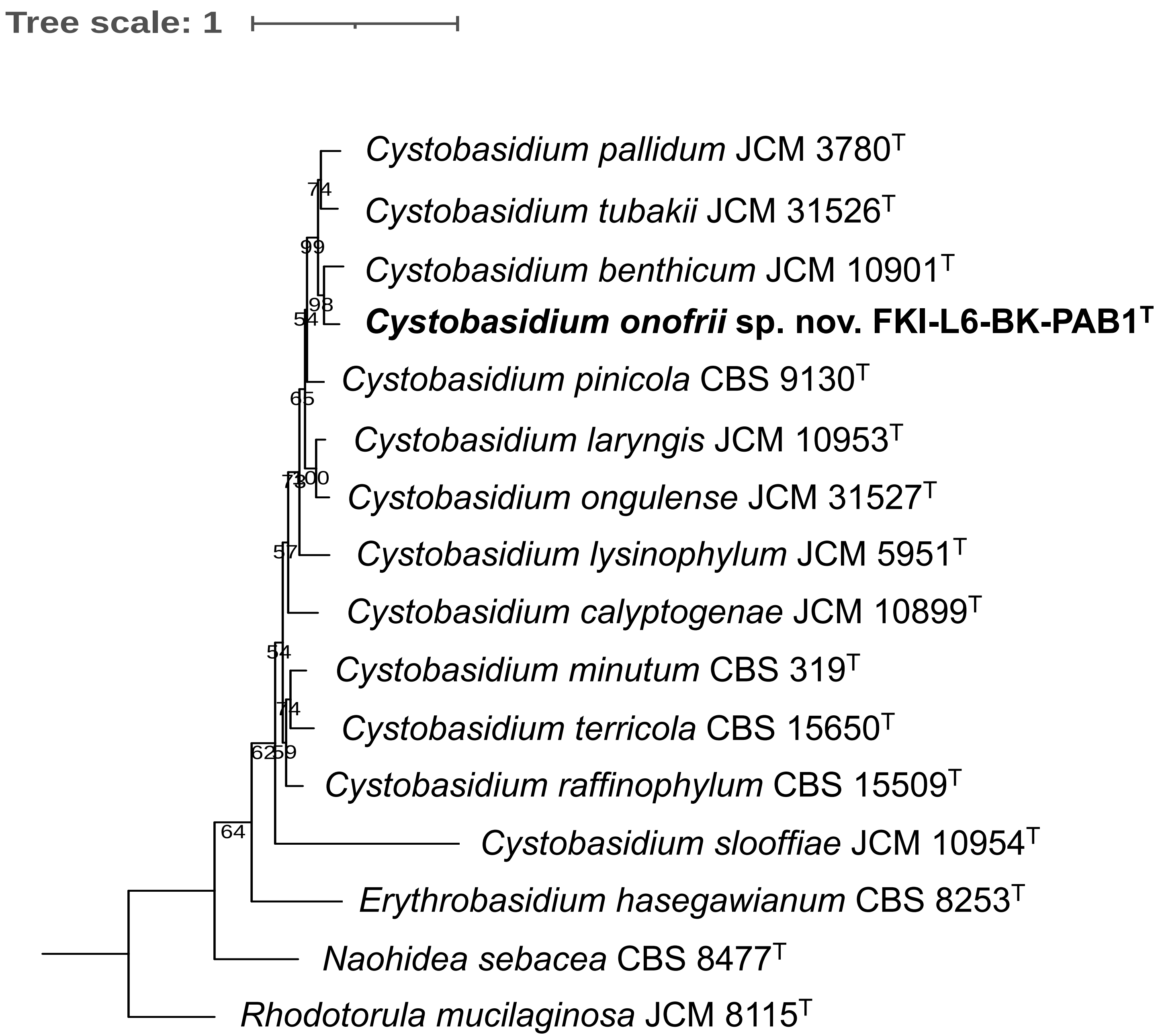

Supplemental Figure SF18. ***RPB2***

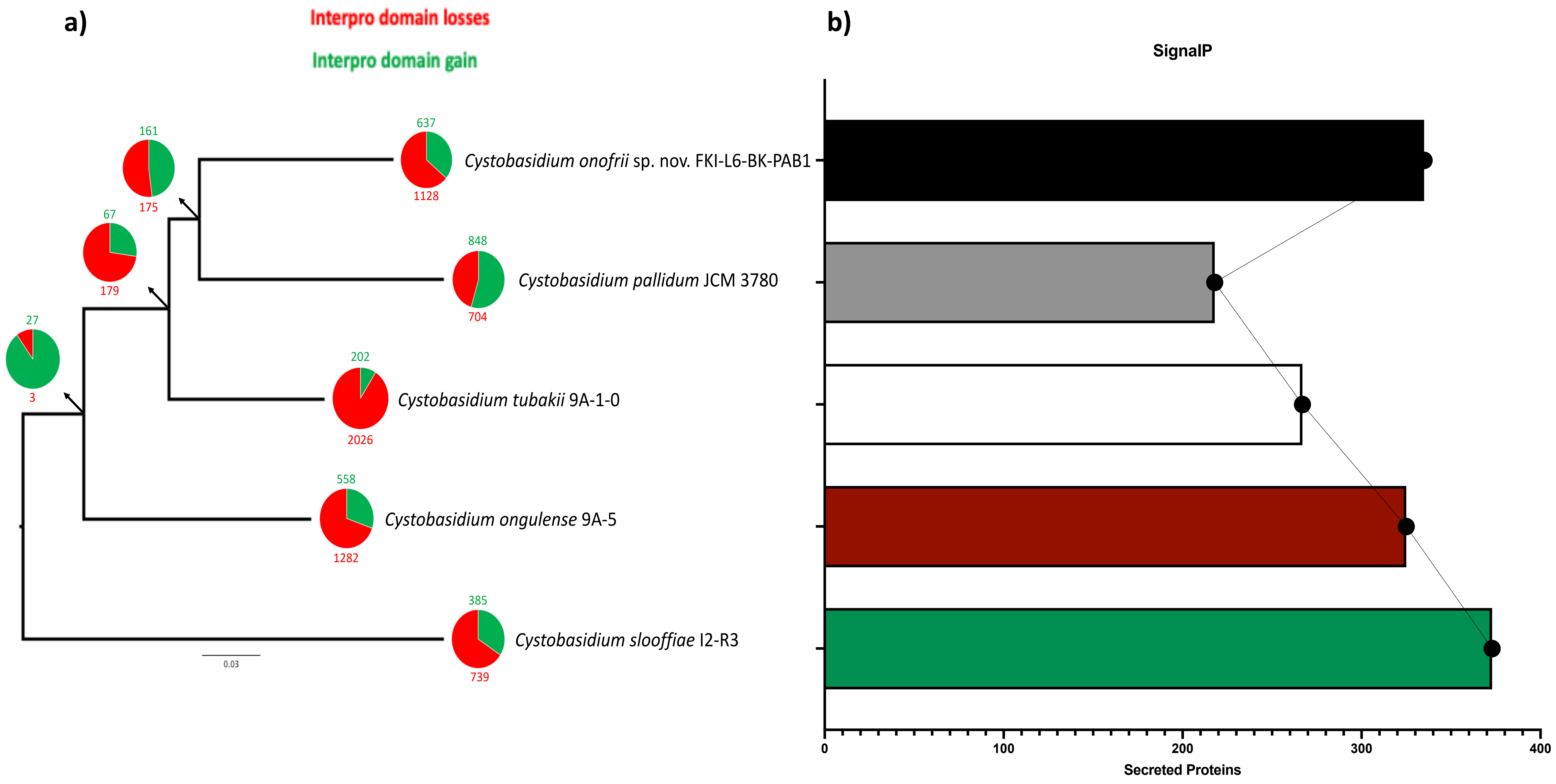

Supplemental Figure SF19.

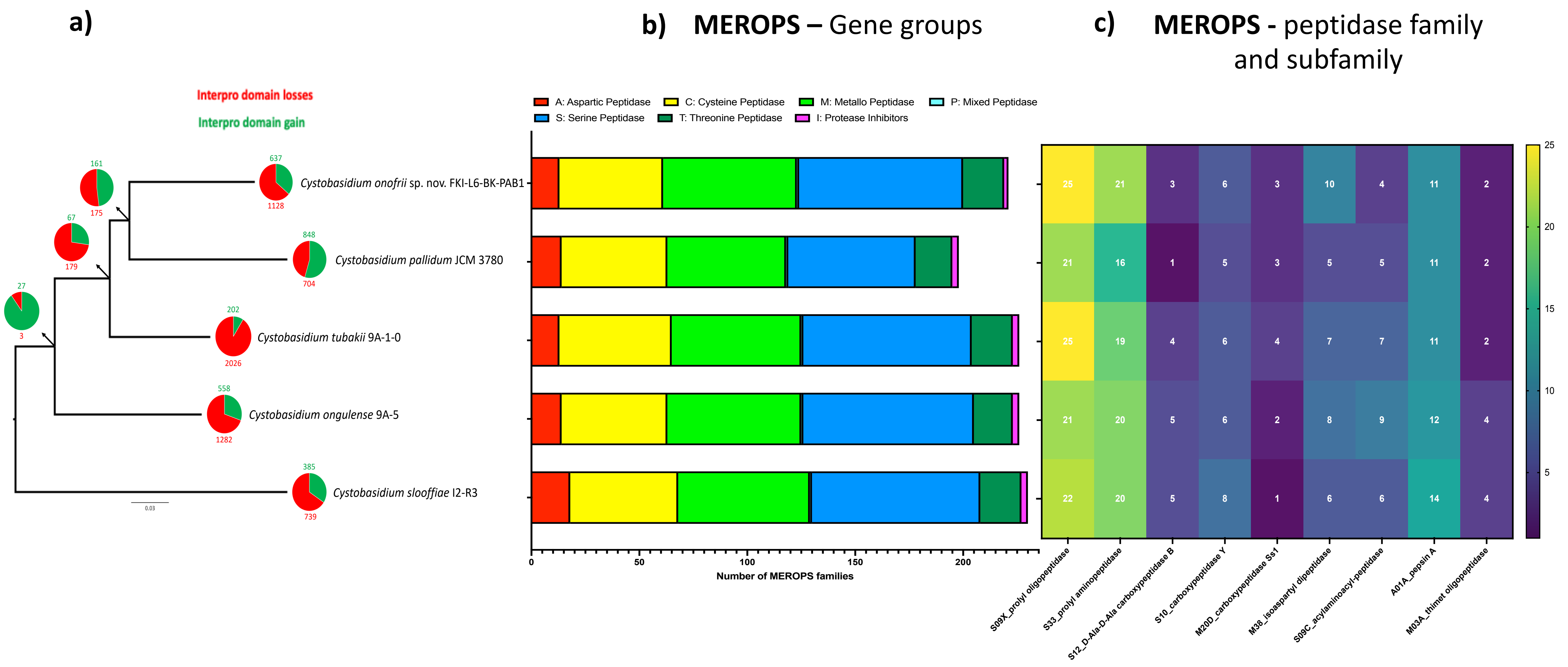

Supplemental Figure SF20.

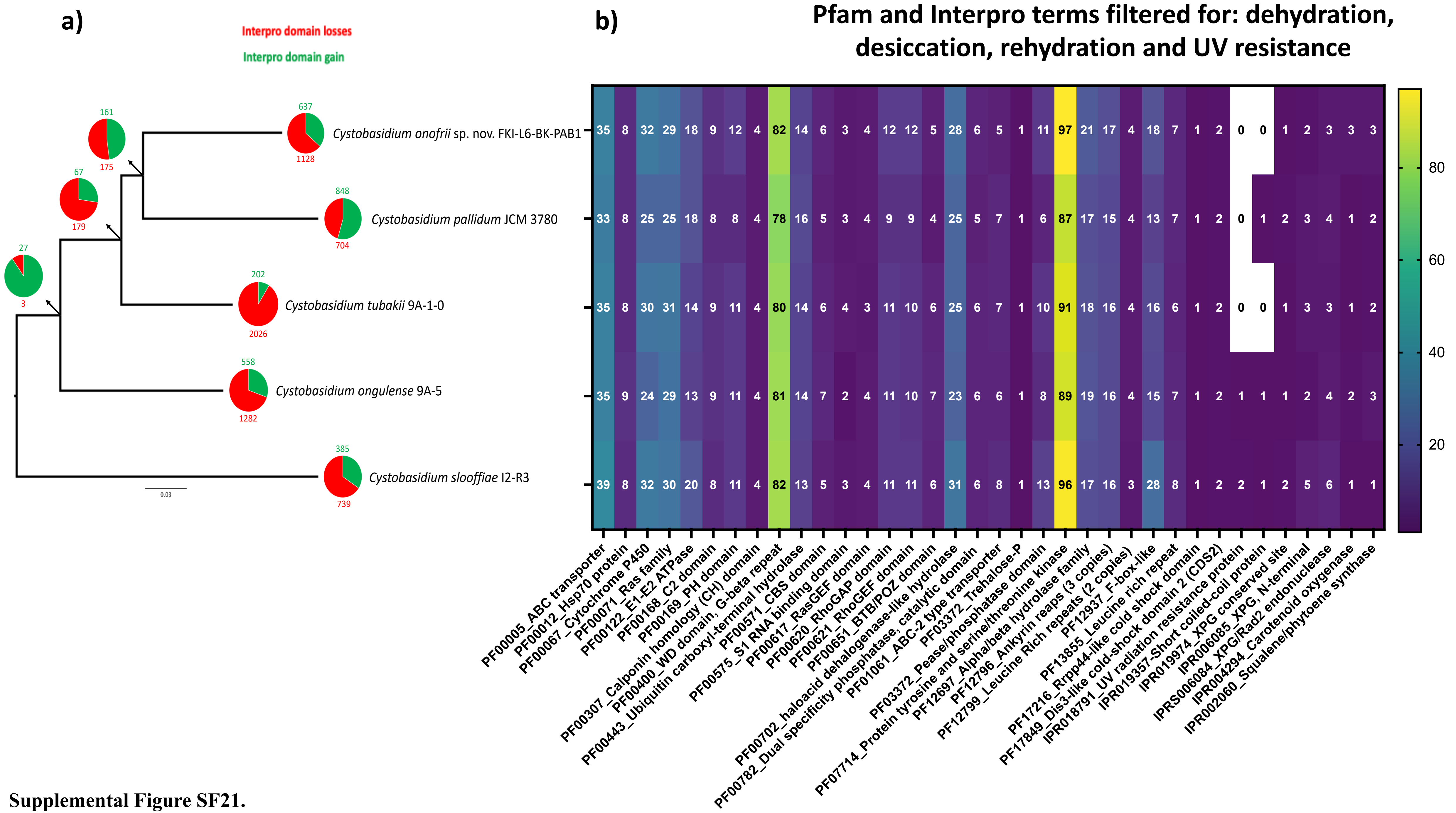

Supplemental Figure SF21.

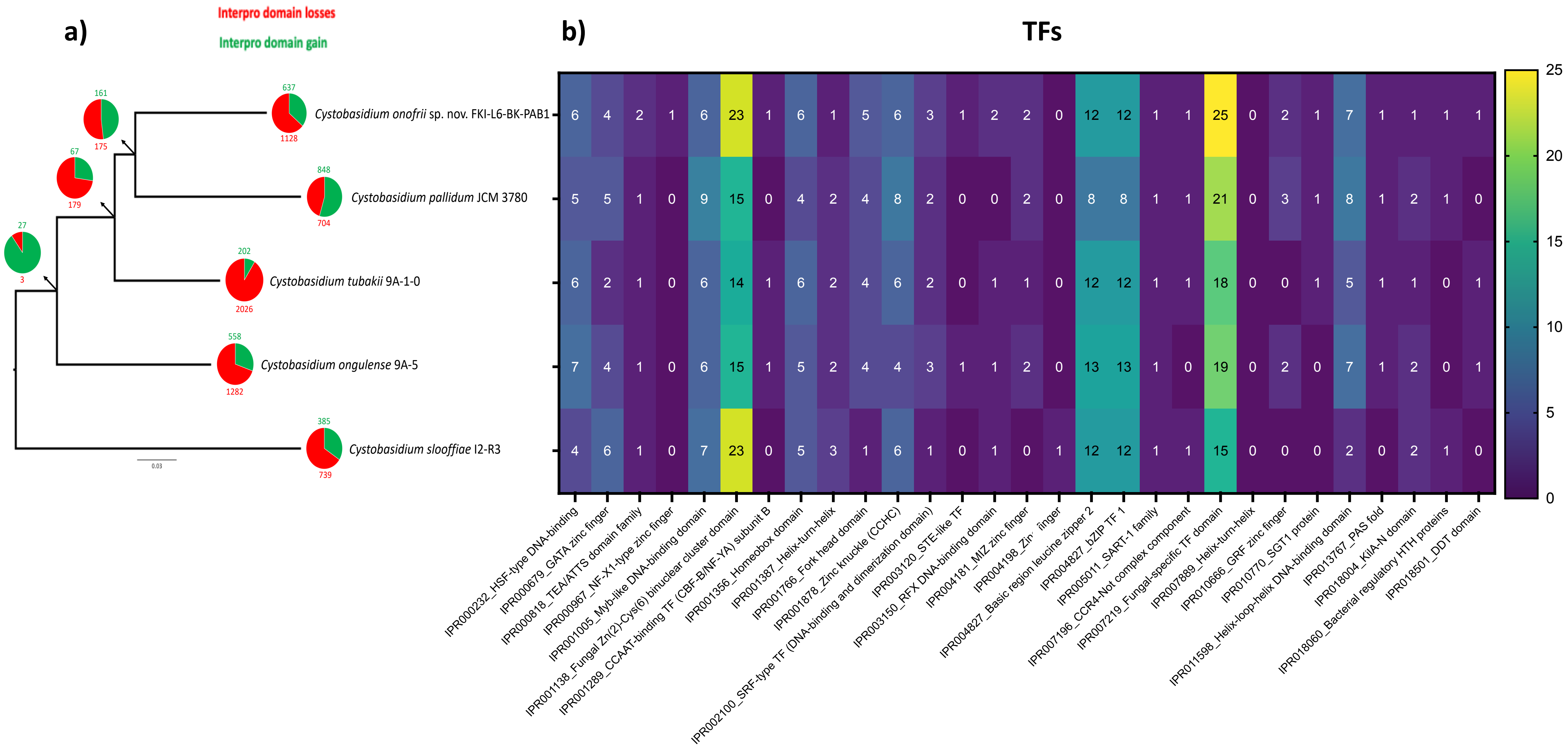

Supplemental Figure SF22.

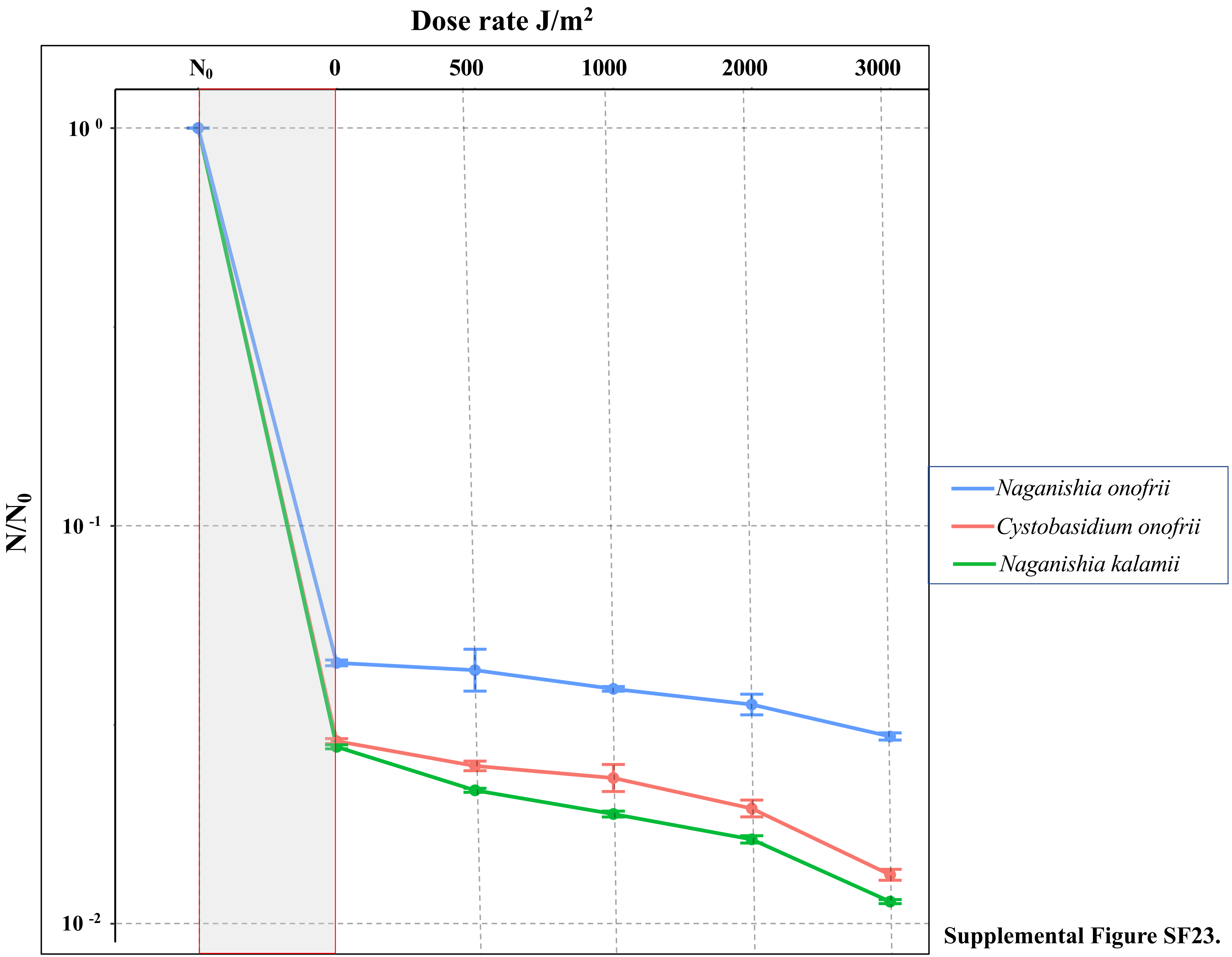

Supplemental Figure SF23.

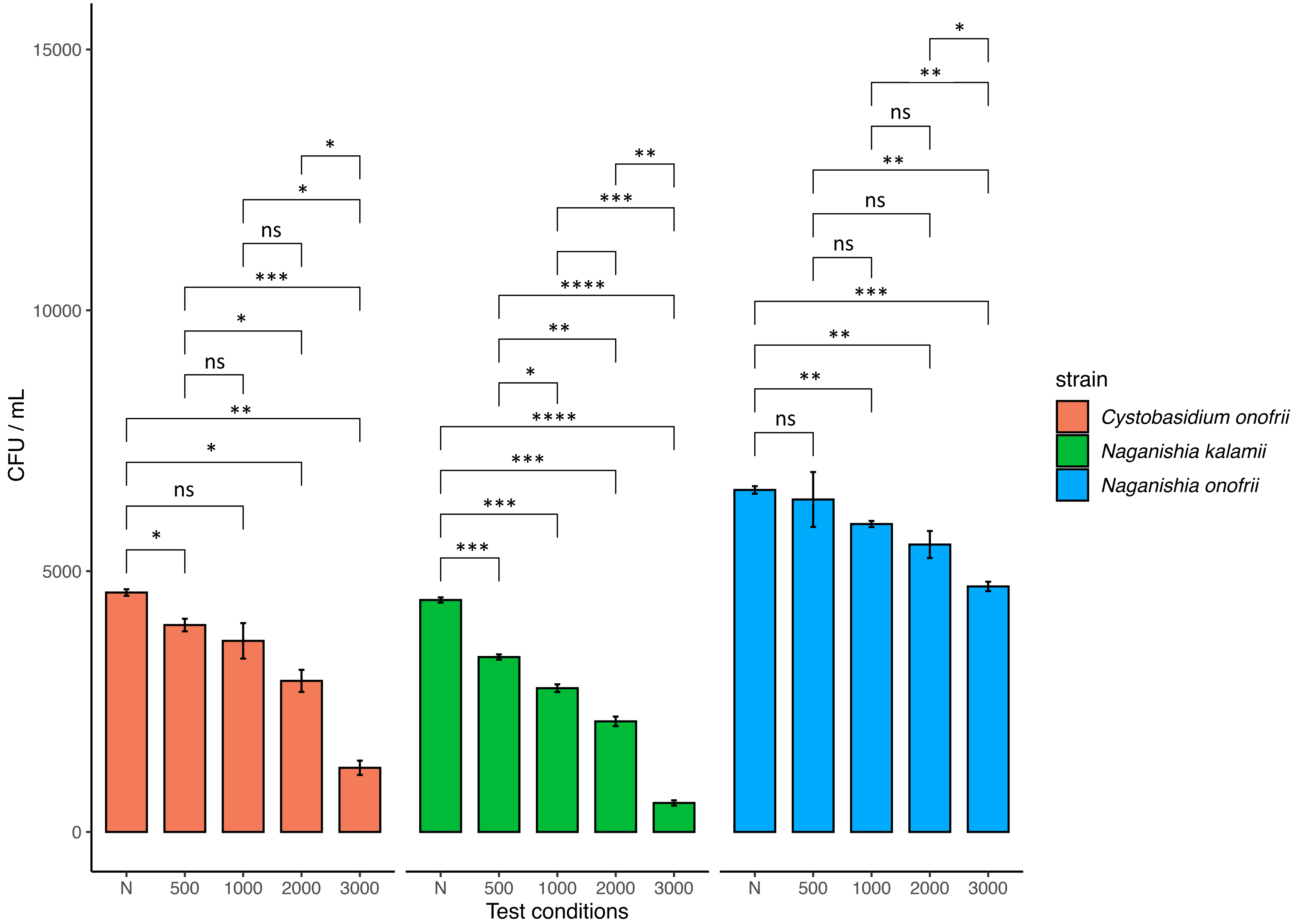

Supplemental Figure SF24.
